# Superior precision of clinical predictions after CD3-relativisation to align flow cytometry data

**DOI:** 10.1016/j.ebiom.2026.106427

**Published:** 2026-08-12

**Authors:** Gunther Glehr, Katharina Kronenberg, Fabiola Arella, Michael Kapinsky, David Segundo, María Iglesias-Escudero, Maria Xydia, Hendrik Veltman, Tobias Schmidt, Viola Hähnel, Víctor J. López-Madrona, Thomas Dienemann, Hans Jürgen Schlitt, Edward Kenneth Geissler, Gero Brockhoff, Silvia Gregori, Philipp Beckhove, Rainer Spang, Eva Martínez-Cáceres, Marcos López Hoyos, Paloma Riquelme, James Alexander Hutchinson

**Affiliations:** a Department of Surgery, University Hospital Regensburg, Regensburg, Germany; b Beckman Coulter Life Sciences GmbH, Krefeld, Germany; c Division of Immunology, Hospital Universitario Marqués de Valdecilla, IDIVAL, Santander, Cantabria, Spain; d Immunology Division, LCMN, Germans Trias i Pujol University Hospital and Research Institute, Campus Can Ruti, Badalona, Spain; e Division of Interventional Immunology, Leibniz Institute for Immunotherapy, Regensburg, Germany; f Bavarian Cancer Research Centre (BZKF), Regensburg, Germany; g Department of Transfusion Medicine, University Hospital Regensburg, Regensburg, Germany; h Inst Neurosci Syst, INSERM, INS, Aix Marseille Univ, Marseille, France; i Department of Gynecology and Obstetrics, University Medical Centre Regensburg, Germany; j San Raffaele Telethon Institute for Gene Therapy (SR-TIGET), IRCCS San Raffaele Scientific Institute, Milan, Italy; k Department of Internal Medicine III, University Hospital Regensburg, Regensburg, Germany; l Department of Statistical Bioinformatics, University of Regensburg, Regensburg, Germany

**Keywords:** Multi-centre clinical study, Biomarker reproducibility, Immunology, Flow cytometry, Instrument harmonisation

## Abstract

**Background:**

Flow cytometry captures subtle changes in immune cell distributions caused by disease, but its full potential in medical decision-making is presently limited by technical variability across instruments, sites and time. To accelerate development of generalisable diagnostic, prognostic or predictive clinical tests, we assembled a benchmark flow cytometry dataset over 20 months using 6 cytometers at 4 independent laboratories in Spain and Germany. Cohorts were amalgamated using a new alignment strategy, *CD3-relativisation*.

**Methods:**

Four hundred and eighty-two clinical flow cytometry samples from 381 healthy donors and a further 100 samples from post-surgical patients admitted to intensive care were stained with a 10-colour T cell marker panel. To align data from different cohorts, we introduced CD3-relativisation, a method for normalising fluorescence intensities per-channel against CD3 signals. The quality of data alignment was evaluated using optimal transport distances (OTD), clustering consistency and predictive performance.

**Findings:**

Our fully annotated data resource (Zenodo 17094078) revealed systematic biases in flow cytometry measurements across time, locations and cytometers. CD3-relativisation minimised these biases without sacrificing biological information. Cell clustering performance and sample-to-sample variability improved after relativisation. Consequently, we were able to predict CMV-IgG serostatus, age and sex with superior precision without relying upon external calibrators, measurement of paired samples, batch definitions or data sharing. Models established in healthy control populations were transferable to a cohort of critically unwell, post-surgical patients.

**Interpretation:**

Our CD3-relativised benchmark dataset establishes a robust standard for evaluating computational methods in clinical cytometry, especially their stability over time and generalisability between instruments, laboratories and clinically heterogeneous populations.

**Funding:**

This work was supported by the BMS-Foundation (FA-19-009), BZKF (BF/04/R/Hutch), EU-H2020 (Immutol_101080562, exTra_101119855, PAVE_861190), BMBF (01KD2206I) and DFG (403161218).

**Research in context**

**Evidence before this study**

Cells of the immune system constantly patrol through blood, lymphoid organs and peripheral tissues, searching for molecular signals associated with disease. The cell types and receptor systems involved in this surveillance are exquisitely sensitive and specific, allowing the immune system to discriminate between many different pathological insults, such as bacteria, viruses, fungi, parasites, cancers or sterile injuries. The sensing cells then trigger an appropriate immune response by stimulating the activation, relocation and proliferation of specialised effector cells. By unscrambling the complicated patterns of immune reactivity associated with particular diseases, we aim to make fast, reliable diagnoses by flow cytometry profiling of leucocyte subsets in peripheral blood. Flow cytometry is widely used in categorising haematological malignancies and monitoring infectious diseases, but its broader application is presently limited by inconsistencies in measurements between instruments, laboratories and time points. Existing strategies for correcting this variability include methodological harmonisation, calibration against external standards and computational normalisation methods. Each of these approaches has limitations in terms of cost, complexity and loss of biological information, meaning that progress towards generalisable, automated diagnostic models remains problematic.

**Added value of this study**

This study aims to accelerate the development of flow cytometry-based diagnostic tools by introducing CD3-relativisation, a simple data alignment strategy that uses CD3 signals as an internal reference feature for normalisation. We then applied CD3-relativisation to assemble an aligned benchmark dataset of 482 samples from 381 healthy donors collected over 20 months on 6 cytometers in 4 laboratories. We demonstrate that CD3-relativisation minimises systematic technical error without sacrificing biological signals, resulting in superior precision when predicting age, sex and CMV-IgG serostatus. Importantly, predictive models trained on healthy control populations also performed well in critically ill, post-surgical patients admitted to ICU.

**Implications of all the available evidence**

CD3-relativisation is a simple and scalable approach to aligning flow cytometry data across instruments, locations and time, making it particularly valuable for longitudinal and multi-centre clinical studies or routine diagnostics. Through application of CD3-relativisation, we created a well-aligned, suitably sized clinical flow cytometry dataset that captures important sources of technical variability in real-world measurements. This resource constitutes a robust standard for evaluating computational methods in cytometry, enabling benchmark comparisons and ensuring generalisability.

## Introduction

Subtle changes in the distribution of cell subsets in blood encode information about an individual's health.^1^ These features can be captured by flow cytometry, a powerful analytical method for measuring expression of multiple markers in millions of single cells from one sample. In clinical practice, flow cytometry is used to diagnose haematological malignancies and monitor viral infections; however, its full potential in clinical decision-making is not yet realised.^2^ More sophisticated applications, especially automating clinical diagnoses based upon flow cytometry results, are mainly limited by the difficulty of calibrating measurements between instruments and across time. State-of-the-art solutions rely upon standard reference materials, such as fluorescent beads or preserved cells, or measurement of paired samples.^3^ Unfortunately, these technical fixes are laborious, expensive and unreliable outside specialised diagnostic laboratories.^4^ To move forward, the field needs a robust method for aligning flow cytometry data without external calibrators, paired sample measurements or exchange of data between instruments.

Developing analytical tools for cross-platform flow cytometry analyses is limited by the lack of publicly accessible, suitably large and annotated datasets.^5^ To rectify this situation, we present a benchmark dataset comprising clinical flow cytometry measurements from 482 samples of 381 healthy human donors made over 20 months using 6 alternative instruments at 4 independent laboratories. Our motivation for collecting this data was to introduce a principle for aligning flow cytometry data, so-called *relativisation*, and then compare the performance of sample classification algorithms with or without alignment by relativisation. To this end, each sample is linked to many demographic and clinical parameters that may or may not be predictable. In diagnostic applications, samples must be evaluated singularly or in small batches as patients present to clinic; therefore, in this study, we processed fresh samples in a clinically relevant timeframe. To capture lab-to-lab variability, unmatched samples were analysed at independent sites in Germany and Spain. The main site at University Hospital Regensburg, Germany, measured paired samples on two instruments, a Navios™ and a CytoFLEX LX™. In collaboration with the Leibniz Institute for Immunotherapy (LIT) in Regensburg, we measured paired samples on our Navios™ and CytoFLEX LX™, as well as an LSRFortessa™ at LIT. The site in Santander, Spain, used a DxFLEX cytometer. The site in Badalona, Spain, allowed access to another LSRFortessa™ and a spectral cytometer, the Cytek Aurora™. All cytometers were set up by blinded operators without external calibrators, communicating results or exchanging samples. The entire dataset has been deposited at multiple stages of pre-processing in the Zenodo repository.^6^ Each data file is fully annotated and is accompanied by technical controls.^7^ Consequently, this dataset offers a comprehensive and versatile resource, capturing technical variation both over time and across international sites.

In flow cytometry, we often talk about an analytical workflow, a stepwise processing of data leading to interpretable results.^8^ Our standard workflow begins with raw datafiles in FCS format, which are manually recompensated or unmixed, quality-controlled by visual inspection and pre-gated on relevant populations.^9^ Next, the data are arcsinh transformed and then clustered with FlowSOM. Finally, hyperparameter-optimised random forests are used to predict sample-related qualities based upon clustered cell frequencies. Our data resource will be invaluable to others seeking to automate or benchmark various aspects of this workflow.

Systematic variability in flow cytometry measurements occurs as: (1) methodological variability owing to inconsistencies in sample preparation or day-to-day differences in cytometer performance; (2) trends in measurements made with one cytometer over time, sometimes called *drift*, owing to gradual changes in laser alignment, fluctuations in laser power or detector efficiency; and (3) discontinuous differences in measured values, known as *shift*, primarily owing to cytometer- or reagent-related batch effects. Variability may affect particular detection channels (e.g., optical filters, detectors, or their amplification settings), be confined to individual lasers (e.g., due to laser power or alignment changes), or be global, affecting all channels across one or more lasers. We previously described additive and multiplicative errors in untransformed fluorescence intensities, which can be channel-specific or affect all channels.^10^ To a large extent, experienced operators can subjectively adjust for such variations by modifying the gate positions or compensation strategy^9^^,^^11^; however, for automated analyses, correcting technical inconsistencies is still an active area of research.^12^, ^13^, ^14^, ^15^, ^16^

Qualitatively, flow cytometrists often compare univariate or bivariate density plots of fluorescence intensities. Quantitative comparison of flow cytometry samples requires measures of distance between cell marker distributions. To compare fluorescence intensities, others have used Earth Mover's distances,^17^, ^18^, ^19^ probability binning,^20^ chi-square, Kolmogorov-Smirnov^21^ and Mahalanobis distances,^22^ or comparison of clustering accuracy between paired samples.^23^ Here, we extend the Earth mover's distance to the Optimal Transport Distance (OTD), giving us a distance between samples for all markers simultaneously.^24^, ^25^, ^26^ We show that OTD is a useful metric for comparing samples, clusters or metaclusters.

Removing measurement errors is a critical step in flow cytometry data analysis. Currently, there are two main approaches to aligning data—namely, *calibration* against an external standard and computational *normalisation*. External calibration depends upon strict standard operating procedures (SOPs) including sample preparation, instrument adjustment according to reference material, pre-specified panels and precise compensation. EuroFlow,^27^ the Human Immuno Phenotyping Consortium approach (HIPC)^28^ or Harmonemia^29^ are prominent examples of this approach, each relying upon manually adjusting the cytometer settings to bring the median fluorescence intensity (MFI) of a reference material to a predefined target value. Alternatively, or after calibration, computational normalisation adjusts data after acquisition. Notably, CytoNorm uses information from samples paired across batches, whereas flowStats or Cytofln align “anchors” in the data.^30^, ^31^, ^32^ CytoNorm2.0, a recent extension of CytoNorm technically permits normalisation between batches without paired samples.^33^

We propose a third approach to aligning data, so-called *relativisation*, which involves normalisation to an internal reference and therefore does not depend upon calibration materials, paired samples or example data. We relativise channel-wise fluorescence intensities to the median CD3 expression measured in the corresponding channel. Because CD3 expression in T cells is typically consistent between donors,^11^ our approach corrects for technical differences in cytometer performance and between instruments. Furthermore, it converts otherwise unitless fluorescence intensities to a common scale that is comparable across all channels. In principle, these relativised units could be transformed to absolute units using a signal intensity calibrator measured in any single channel.

In this study, we evaluate relativisation as a strategy for harmonising flow cytometry data from diverse, real-world clinical sources, spanning both conventional and spectral platforms. We created a benchmark dataset to predict age, sex and CMV-IgG seropositivity because these features can be reliably determined from clinical notes and lab tests.^34^ Relativisation reduces systematic bias, aligns clustering results across cytometers and yields more consistent clinical predictions over time. Beyond establishing a robust analytical framework, we provide an open, comprehensive dataset designed to enable objective, reproducible benchmarking of computational methods for clinical flow cytometry. Together, these contributions advance the integration of high-dimensional immune profiling into reliable, multi-centre clinical decision-making.

## Methods

### Patients

Blood samples were collected from healthy donors or patients admitted to surgical ICU at UKR, HUMV and IGTP. The study was conducted in multiple phases: R1 and R2 in Regensburg (18 July, 2023-23 Jan, 2024, and 15 May, 2024-16 July, 2024), SAN in Santander (29 April, 2024-28 May, 2024), FORT in Regensburg (14 January, 2025-24 January, 2025), BAD in Badalona (18 March, 2025-19 March, 2025) and ICU (01 October, 2024-09 May, 2025). Standard biochemical, haematological and microbiological investigations were performed as routine diagnostics at UKR. CMV-IgG status was tested using the Architect CMV IgG reagent (Abbott GmbH). Complete descriptions of each cohort (Supplementary Note S1) and clinical results (Supplementary Note S2) are provided as Supplementary Material.

### Flow cytometry measurements

Blood was collected into EDTA-vacutainers by peripheral venepuncture and then delivered to the responsible lab at ambient temperature. Samples were stored at 4 °C for up to 4 h before processing. Whole blood samples were stained as previously described using the DURAClone IM T Cell Subsets Tube (Beckman Coulter, B53328, Table S1) and single-staining controls (Table S2). Briefly, we stained whole blood for 15 min, lysed erythrocytes with Versalyse Solution (Beckman Coulter) and then washed once with DPBS before fixation with IOTest 3 Solution (Beckman Coulter). Step-by-step procedures can be accessed through protocols.io.^35^ DURAClone IM T cell subsets compensation tubes were run every two weeks. Daily quality control (QC) checks were run on all cytometers using Flow-Check Pro Fluorospheres (Beckman Coulter, A63493) to ensure proper function.

Data were collected in Regensburg using a Navios™ cytometer running Navios™ Cytometry List Mode Acquisition Analysis Software, Version 1.3 (Beckman Coulter) or a CytoFLEX LX™ cytometer running CytExpert, Version 2.4.0.28 (Beckman Coulter) or a BD LSRFortessa X-20 running BD FACSDiva software v9.0. In Santander, data were collected using a DxFLEX cytometer running CytExpert for DxFLEX, Version 2.2.0.7. In Badalona, data were collected using both a Cytek Aurora™ 5 L spectral flow cytometer running SpectroFlo® software (Cytek Biosciences), Version 3.1.0, and a BD LSRFortessa 4 L flow cytometer running BD FACSDiva™Software (BD Biosciences), Version 6.2. Settings for each cytometer were established by independent experienced operators without exchange of reference samples, calibration materials or example data.

### Flow cytometry analyses

All computations were performed in R^36^ using the Bioconductor packages flowCore^37^ and flowWorkspace,^38^ alongside our own convenience tools, cytobench,^39^ cycompare^40^ and otcyto (python).^41^ We gathered FCS files from all instruments, and carried out pre-processing, clustering, classification and optimal transport analyses.

To maximise the value of our data resource, we adopted a conventional, stepwise approach that may be optimised by other users. Accordingly, we deposited all data in raw form and after each pre-processing step, with and without applying relativisation. Each FCS file contains three compensation matrices: (1) automatically calculated from bi-weekly compensation controls; (2) automatically calculated from CD3 single-stain controls; and (3) automatically calculated from CD3 single-stain controls, then manually refined in Kaluza by an experienced operator on a sample-by-sample basis.

Pre-processing followed four steps: (1) compensation or unmixing by an experienced operator; (2) manual gating to identify CD45^+^ CD3^+^ singlet T cells in panel samples and lymphocytes in single-stained controls; (3) arcsinh transformation of fluorescence intensities using manually selected cofactors (Table S3); and (4) random subsampling of 10^4^ CD3^+^ T cells to standardise sample sizes. Optionally, relativisation was applied as an alignment strategy before step 3. CytoNorm normalisation was applied after arcsinh transformation.

Data were then exported as FCS files for upload to Zenodo.^42^ These FCS files contain the uncompensated data and three compensations: 1) single-stain compensation for each sample, 2) compensation from DURAClone IM T Cell Subsets compensation tubes, 3) manually recompensated single-stain compensation for each sample. Manual gating for each sample is provided as FlowWorkspace^38^ gating sets in h5 format and should be applied to the manually recompensated data. An example gating strategy is provided (Figure S1). In addition, we provide manually recompensated, pre-gated T cell FCS files for all fully stained samples. Our FCS file nomenclature is explained in Tables S4 and S5.

For reproducibility, R1 and SAN donors were randomly assigned to training, validation, and test sets only once. This donor-level split was preserved for all downstream analyses and serves as the foundation for model development and evaluation. Ideally, future benchmarking efforts using our data resource should report results using these splits for direct comparability.

After pre-processing, we performed clustering using FlowSOM on training samples from each cytometer separately, constrained to 400 clusters and 98 metaclusters according to the number of expected biological subpopulations.^1^ These FlowSOM parameters placed our models well within the plateau of classification performances on our validation set (Figure S2). To ensure comparability across samples, we standardised the number of cells to 10,000 per sample. For samples with fewer than 10,000 cells, we replicated cells to reach this target. Replication was performed uniformly, minimising duplication of individual cells. Each final clustering was then applied to every sample from all cytometers without retraining.

We trained random forest models to predict CMV-IgG serostatus, age, and sex using FlowSOM metacluster proportions as input. For each outcome, models were trained with and without relativisation on three datasets: R1-Navios, R1-LX, and SAN-DxFLEX. Hyperparameters were tuned on validation data with an mlr3 based pipeline.^43^ Final models were applied across all cytometers without retraining. Performance was evaluated via AUROC on held-out test sets, the prospective R2 cohort, cross-cohort samples, the FORT, BAD and ICU cohorts.

### CytoNorm normalisation

We followed the approach of Quintelier,^33^ applying CytoNorm for fluorescence normalisation, both before and after relativisation. As reference data, we used first-presentation samples from the R1 cohort measured on the Navios and LX cytometers. For each device, we created an aggregate file composed of 10,000 cells from each of the 50 training samples, yielding a representative dataset of 500,000 cells per cytometer. All processing was performed on arcsinh-transformed data, either with or without prior relativisation. For the ICU cohort analysis, we used 60 randomised samples to build the aggregate file, then applied CytoNorm with 3 batches: Navios, LX or ICU-Navios. Before relativisation, we trained CytoNorm on the aggregated training set and applied it to individual samples. This design avoids paired controls and better reflects clinical workflows, where specimens arrive sequentially and each must yield a stable, independent prediction. However, cross-instrument fluorescence shifts were so pronounced that most metaclusters became instrument-specific, undermining alignment. After relativisation, we constructed target quantiles from the aggregate training samples and applied CytoNorm sample-by-sample, normalising each towards the training targets.

### Within and between cytometer comparisons

Comparison between cytometers was visualised on positive population MFIs per fluorochrome, optimal transport distances and by applying FlowSOM clustering on training samples from R1-Navios, R1-LX and SAN-DxFLEX. We then used the proportion of clusters and metaclusters of all samples to perform principal component analysis (PCA) and visualise the samples in PCA space.

Predicted probabilities were investigated using paired measurements of R1-Navios, R1-LX, R2-Navios and R2-LX using the intraclass correlation coefficient, ICC(2, 1).^44^ The ICC(2, 1) quantifies the consistency of the predicted probabilities between the two cytometers. The ICC(2, 1) is bound at 1, with 1 indicating perfect consistency between the two cytometers. This ICC was calculated on 1) the prospective R2 samples and 2) all or only the test samples of R1 if trained on SAN or R1, respectively.

### Optimal transport distances

Optimal Transport Distance (OTD) quantifies differences between two multivariate cell distributions, such as samples from different time points or cytometers. It reflects the cost required to theoretically transform one sample of cells into another, considering both the number and magnitude of cell shifts. By incorporating all markers simultaneously, OTD provides a holistic measure of sample dissimilarity, with higher values indicating greater divergence. We developed otcyto,^41^ a python package for convenient calculation of optimal transport distances from flow cytometry data. It is based on the python library geomloss^25^ and provides a simple interface to calculate OTD between samples of many cells. otcyto is available on GitHub and per default implements the de-biased Sinkhorn divergence.

In this work, we used OTD to quantify dissimilarities between samples measured on different cytometers over time with or without alignment. A representative sample served as the reference, created by concatenating all training samples from all cytometers and subsampling to a fixed size of 10,000 cells. For each sample, we randomly subsampled 10,000 cells and calculated its OTD relative to the master sample. This approach enabled standardised comparisons of samples across cytometers and time points. The debiased Sinkhorn divergence was used as the OTD metric, with a blur parameter of 0.75, a scaling factor of 0.7, and the squared Euclidean distance ($\frac{1}{2}{\left|x-y\right|}_{2}^{2}$) as the cost function.

A full description of our implementation of OTD is provided as Supplementary Note S3.

### Quantification of instrument agreement using optimal transport distances

To statistically compare instrument agreement across processing variants, we computed per-sample absolute OTD differences between paired Navios and LX measurements: |Δᵢ| = |OTD (sampleᵢ, Navios) − OTD (sampleᵢ, LX)|. OTD values are valid for comparisons within a processing variant but are not directly comparable across variants, because we used a different reference point: each variant produces its own transformation of the reference cells and the distance metric operates in different transformed data spaces. Paired instrument differences avoid this limitation, as both devices share the same reference within each variant. Differences in |Δ| between processing variants were tested using a two-sided Wilcoxon signed-rank test. To assess temporal stability, we fitted a linear mixed-effects model (lmerTest package^44^) with |Δ| as the outcome, processing variant and days since first measurement as fixed effects (including their interaction), and sample identity as a random effect. Consistent results were obtained when substituting study period (R1 vs R2) for continuous time.

### Relativisation: a method to align flow cytometers

**Fig. 1:**
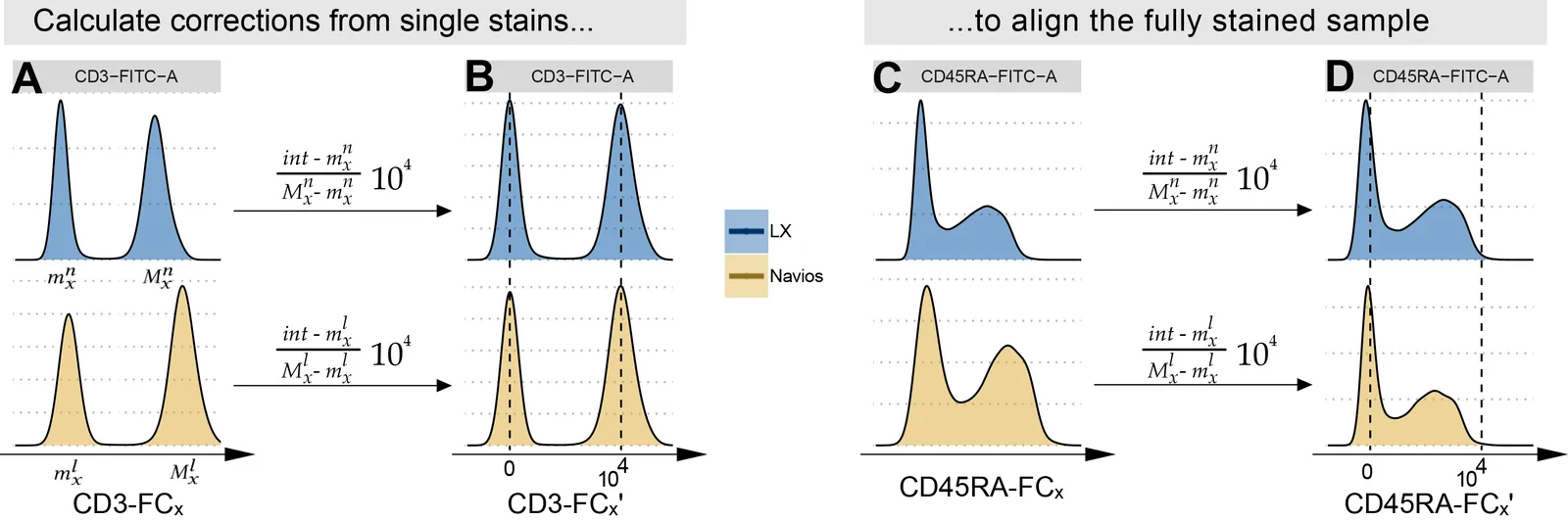
CD3-relativisation aligns fluorescence signals across cytometers. CD3-relativisation is a simple yet robust strategy to align fluorescence intensities across cytometers and over time. We illustrate this approach with the example of aligning CD45RA-FITC signals from a representative paired sample measured in parallel on Navios™ and CytoFLEX-LX™ cytometers. The same principle applies to each channel in a panel. Paired samples are shown here for illustration only: in practice, relativisation is applied independently per sample. All channels are shown in Figure S3. **(A)** We begin with CD3-FITC single-stained samples to estimate the median FITC signal of CD3^+^ and CD3^−^ lymphocytes on each cytometer. **(B)** These median values define a linear transformation that rescales CD3-FITC intensities: the median signal of CD3^+^ cells is set to 10^4^, and that of CD3^−^ cells to 0. **(C)** We then examine fully stained samples measured in parallel: before applying relativisation, CD45RA-FITC signals differ between cytometers. **(D)** Applying the transformation derived from CD3-FITC aligns CD45RA-FITC signals across devices. This correction uses CD3 as an internal reference and preserves biological differences, provided that measurements remain within the cytometers' linear ranges. Crucially, CD3-relativisation requires no external calibrators, paired samples, or shared data between instruments. Given consistent staining, it can be applied to all markers in a panel, yielding a common scale of relative CD3 expression units.

To solve the problem of generalising predictive models across cytometers, we introduce relativisation (Box 1; Fig. 1). CD3-Relativisation normalises fluorescence signals within each channel against CD3. CD3 was chosen because its surface expression on T cells is biologically stable across donors.^45^ For each of the 10 fluorochrome channels in our panel, a single-stained CD3 (UCHT1) control was prepared from the same material as the fully stained sample, then both were measured on the same cytometer using the same settings. Each CD3 single-stain control was compensated and gated on lymphocytes. CD3^−^ and CD3^+^ populations were identified by k-means clustering (k = 2). The median fluorescence intensities of these two populations define a per-channel linear rescaling that maps the CD3^−^ median to 0 and the CD3^+^ median to 10^4^. Applying this mapping to fully stained samples allows a channel-wise correction of both additive offsets and multiplicative gain differences that arise from variability in sample preparation or instrument performance.

Box 1 **CD3-Relativisation**

**Table:** 

**Goal:** To align fluorescence intensities across cytometers and time points by rescaling each channel to an internal CD3 reference, yielding instrument independent units without external calibrators or data sharing. **Input:** A compensated, fully stained sample (*X* ∈ ℝ^*n*×*p*^; *n* cells, *p* channels). For each channel *x*, a CD3 single-stain control from the same specimen measured with the same cytometer and settings. **Output:** A relativised matrix $\tilde{X}$ ∈ ℝ^*n*×*p*^ on an instrument independent scale. **Algorithm:** For each channel *x* ∈ {1,…,*p*}: 1. Gate the CD3 single-stain control on lymphocytes and separate CD3^−^ and CD3^+^ populations (by gating or clustering). 2. Compute medians of compensated intensities (*int*) in channel *x*: $m_{x}:=\text{median}\left({\mathrm{int}}_{x}|{\text{CD}3}^{-}\right)\text{,}$ $M_{x}≔\text{median}\left({\mathrm{int}}_{x}|{\text{CD}3}^{+}\right)\text{.}$ 3. Relativise every cell *c* in the fully stained sample: ${\tilde{X}}_{c,x}≔\frac{X_{c,x}-m_{x}}{M_{x}-m_{x}}10^{4}\text{.}$ Relativisation is applied before any transformation of scale.	**Properties:** • Corrects additive offsets (*m_x_*) and multiplicative gain (*M_x_* − *m_x_*) per channel, independently. • Reference values come from the same cytometer, time and sample; no calibrators or data sharing. • Units are comparable across channels and cytometers*. **Assumptions:** • Median CD3 surface expression is biologically stable across donors. • Measurements lie within the linear detection range of each cytome ter. • Same antibody clone (here UCHT1) per fluorochrome for all exper iments in a study. ∗Cross-channel comparability requires the same antibody clone in every fluo rochrome used at saturating concentrations; cross-cytometer com parability only requires consistency within each fluorochrome. See Fig. 1 for a visual walkthrough for a single fluorochrome and an ex ample of the same sample measured on two cytometers.

Under saturating antibody binding conditions, relativisation additionally converts unitless fluorescence intensities to a common scale across channels. By separately measuring CD3 in every channel, we can align signals for any panel of markers (Figure S3). Relativised data retain standard FCS structure and are compatible with common downstream analyses, including transformation (e.g., arcsinh or logicle), gating, clustering, classification and optimal transport-based comparisons. Relativisation was applied before arcsinh transformation in all analyses. Although demonstrated here using the DURAClone IM T Cell Subsets Tube, the method is applicable to any marker panel, provided that a reference population with stable expression is available. Relativisation was implemented in the open-source R package cytobench,^39^ available on GitHub.

### Ethics

The study was approved by the Ethics Committees of the University of Regensburg (UKR), Germany (22-2780-101, 23-3519-101), the Hospital Universitario Marqués de Valdecilla (HUMV), Santander, Spain (CS24-116; 2024_6) and the Germans Trias I Pujol Research Institute (IGTP), Badalona, Spain (PI-23-272). The German Register of Clinical Studies lists this study as DRKS00037928. The study was conducted in accordance with the Declaration of Helsinki (2024) and all other relevant national and international laws and guidelines. All donors provided written, fully informed consent to sample collection and study participation.

### Statistics

The number of samples was largely bound by donor availability and the logistical constraints of collecting fresh blood samples across four independent laboratories over 20 months. However, using power.roc.test from pROC and using the R1 test AUROCs as reference (0.95 CMV-IgG, 0.78 age, 0.65 sex), we estimated a power of >90% for CMV and age in our test set of 53 samples (R1), prospective 52 samples (R2) and 153 samples (SAN). For sex, only SAN had adequate power (91%); R1 and R2 were underpowered, consistent with the weak and inconsistent predictability of sex observed across all cohorts.

Temporal trends in fluorescence intensities were visualised using LOESS smoothing with 95% confidence intervals. Predictive performance of FlowSOM followed by random forest models (see Flow cytometry analyses) was evaluated by the area under the receiver operating characteristic curve (AUROC), with 95% confidence intervals estimated by the DeLong method. Classification thresholds were set at the point on the ROC curve with minimum distance to the top-left corner, determined on validation-set data. Cross-cytometer prediction consistency was quantified using the intraclass correlation coefficient, ICC(2,1) (two-way random effects, absolute agreement, single rater), applied to predicted class-membership probabilities from paired samples. All analyses were performed in R or Python.

### Role of funders

The funders had no role in study design, data collection, data analyses, interpretation, or writing of the manuscript.

## Results

### Creating a comprehensive clinical flow cytometry data resource

**Fig. 2:**
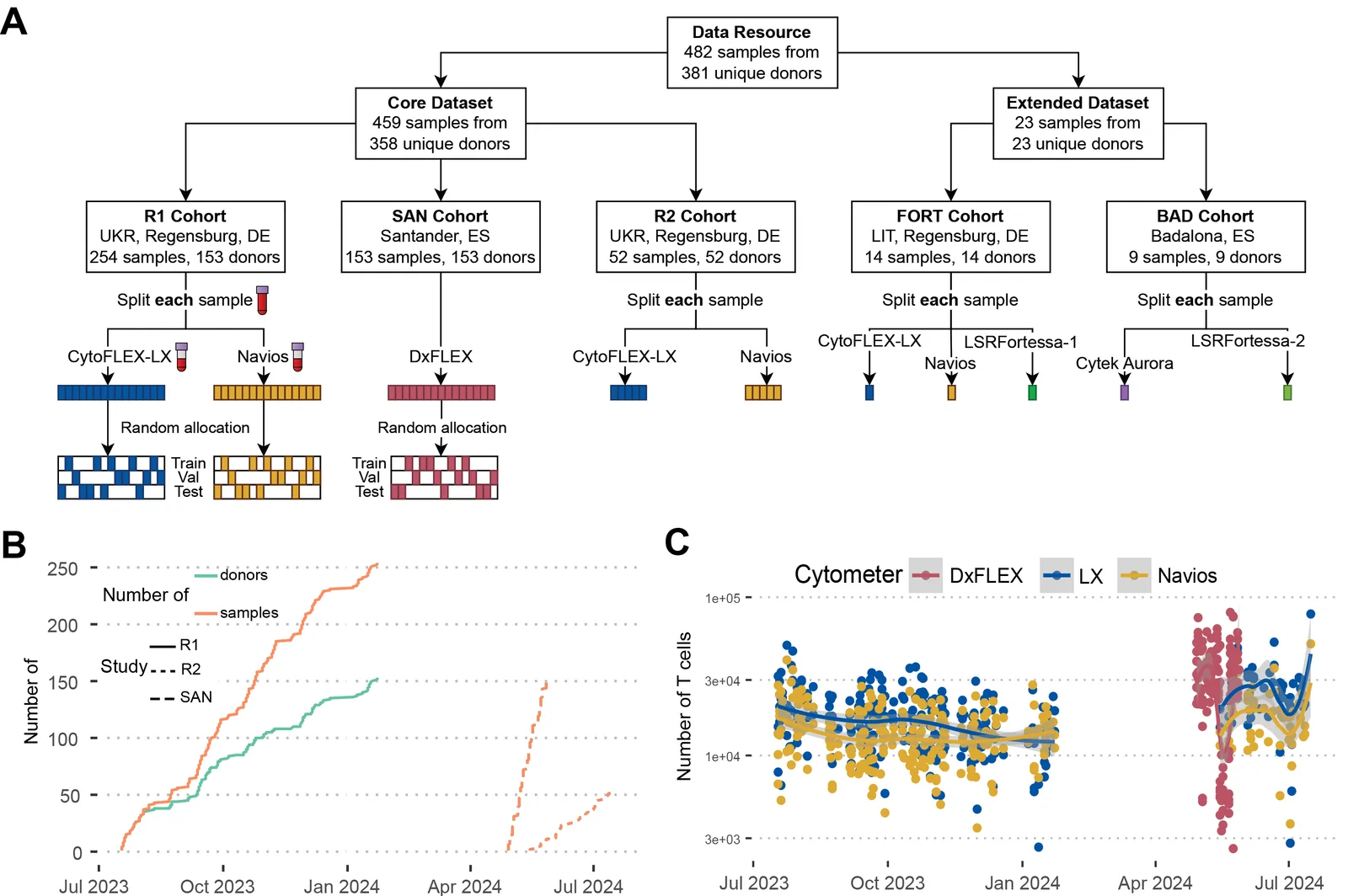
A clinical flow cytometry data resource. **(A)** A data resource comprising 482 samples from 381 healthy human donors was assembled to capture real-world variation in clinical flow cytometry measurements across instruments, laboratories and time. The core dataset contains samples from three cohorts: R1 and R2 were obtained at University Hospital Regensburg, Germany; SAN was collected at the Marqués de Valdecilla University Hospital, Spain. Samples from R1 and R2 were split after staining to make paired measurements using a Navios™ and CytoFLEX-LX™ cytometer. Samples from SAN were measured with a DxFLEX™ cytometer. The extended dataset includes samples from 2 additional cohorts: FORT samples were measured in parallel using Navios™, CytoFLEX-LX™ and LSRFortessa™ cytometers in collaboration with the Leibniz Institute for Immunotherapy, Germany; BAD samples were measured in parallel using a LSRFortessa™ and a Cytek Aurora™ spectral cytometer at the Institute for Health Science Research Germans Trias i Pujol (IGTP) in Spain. **(B)** R1 samples were collected before SAN and R2 samples; therefore, R2 represents a prospective test set. **(C)** T cell counts recorded for each of the 459 core dataset samples over the study period.

This section describes the structure of our data resource and the rationale for its collection (Fig. 2A). Our dataset incorporates a total of 482 samples from 381 unique healthy human blood donors. All samples were stained with the DURAClone IM T Cell Subsets Tube, a 10-parameter assay focussing on stages of T cell maturation (Table S1 and Figure S1). We divide our data resource into a core dataset, intended for computational model development and validation, and an extended dataset, which allows us to generalise our models across cytometry platforms.

Our core dataset comprises 459 samples from 358 unique donors, which are organised into three main cohorts (R1, R2 and SAN). R1 includes 254 samples from 153 unique donors, which was analysed in Regensburg. Clinical and demographic variables are recorded (Table S6). Within R1, 101 repeated samples were collected from 60 donors to test the biological stability of T cell subset distributions over time. The first sample taken from each R1 donor was randomised into training (50), validation (50) and test (53) sets (Table S7). R2 is a prospective dataset collected 4 months after R1 that includes 52 samples from unique donors, who are not represented in R1 (Fig. 2B, Table S8). R1 and R2 samples were split after staining and measured in parallel with a Navios™ (Navios) and CytoFLEX LX™ (LX) cytometer. SAN comprises 153 samples from unique donors that were measured with a DxFLEX™ (DxFLEX) cytometer in Santander (Table S8). As with R1, SAN samples were randomised into training (50), validation (50) and test (53) sets (Table S9).

Our extended dataset incorporates two cohorts, FORT and BAD. FORT comprises 14 samples from unique donors that were split into equal parts, then measured in parallel using 3 cytometers—namely, a Navios™ and CytoFLEX LX™ from Beckman Coulter, and an LSRFortessa™ (LSR) from Becton Dickinson. The BAD cohort incorporates samples from 9 unique donors that were split after staining and measured in parallel using an LSRFortessa™ and a Cytek Aurora™ (CA) spectral cytometer.

To reduce technical variability, all samples were collected, processed and measured according to standard operating procedures. The DURAClone IM T Cell Subsets Tube is a dried-down antibody mixture for staining whole blood that further minimises technical errors.^46^ Our methodological reproducibility is reflected by the consistency of captured T cell counts across our study (Fig. 2C). Crucially, the cytometer settings for each instrument in our study were established independently—explicitly, by independent operators without exchanging samples, calibrators or data.

Information about donors’ age, sex and CMV-IgG serostatus, as well as other demographic and clinical details, are recorded for each sample (Tables S7–S10). Our entire raw dataset, including routine QC tests of cytometers, is publicly available at Zenodo^42^ (nomenclature in Tables S4 and S5).

### Fluorescence intensities vary within and between cytometers

**Fig. 3:**
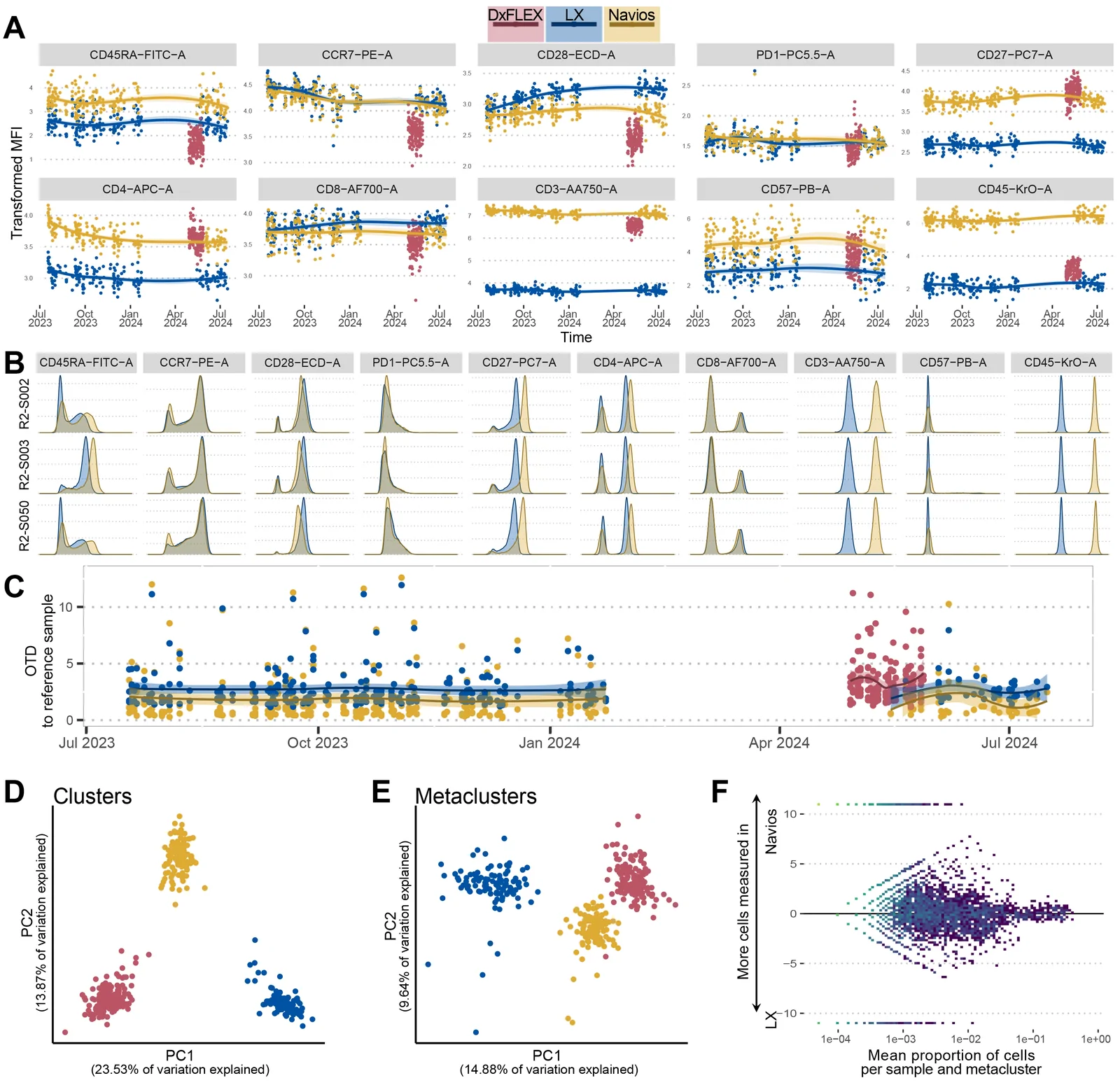
Variability in flow cytometry measurements within and between cytometers. **(A)** Longitudinal flow cytometry analysis of surface marker expression in core dataset samples. Each subplot shows arcsinh-transformed median fluorescence intensities for specific markers in gated T cell subsets over time. Solid lines and shaded areas depict LOESS-smoothed trends and their 95% confidence intervals. **(B)** Density plots of arcsinh-transformed fluorescence intensities from three representative R2 cohort samples that were measured with a Navios™ cytometer (yellow) or CytoFLEX-LX (blue) cytometer. **(C)** Summary of global marker-level differences using optimal transport distance (OTD) to an aggregate reference sample. **(D)** FlowSOM was trained with training samples from R1-Navios, R1-LX and SAN-DxFLEX. This clustering was then applied to all samples. Principal component analysis (PCA) was performed on the resulting cell proportions in all 400 clusters to visualise technical and biological variation between samples. **(E)** PCA of cell proportions in all 98 FlowSOM metaclusters after applying the same FlowSOM model. **(F)** Bland-Altman plot comparing the proportion of cells per metacluster in samples measured on both Navios and LX cytometers for the first sample from each R1 donor and R2. Colour corresponds to the number of sample–metacluster pairs at each position.

We observed consistent fluorescence intensity differences between cytometers and drifting of performance over 12 months (Fig. 3A; FORT, Figure S4A; BAD, Figure S5A). Within-cytometer variation of marker expression over time was evaluated across cytometers using MFI of manually gated cell subsets. LOESS smoothing^47^ was applied to estimate trends and confidence intervals, revealing time- and cytometer-dependent differences in signals. Comparable effects were seen in the MFIs of single-stained controls (Figure S6, Figure S7).

Visually comparing the fluorescence intensities of three examples from the R2 dataset illustrates shifts between data obtained using the Navios and LX (Fig. 3B). Similar differences between paired samples were observed in the FORT (Figure S4B) and BAD (Figure S5B) cohorts. We quantified differences across all markers using Optimal Transport Distances (OTD), a useful metric for the distribution of all measured markers over time, within and between cytometers (Fig. 3C). Specifically, OTD was calculated for all markers relative to a composite sample derived from randomly selected events from SAN and R1 training samples; hence, greater distances imply more dissimilarity from the reference sample.

Variability in R1, SAN and R2 measurements was visualised in a principal component plot, showing that FlowSOM^48^ clustering (Fig. 3D) and metaclustering (Fig. 3E) were dominated by differences between instruments. Similar differences between cytometers were found in the FORT (Figure S4C and D) and BAD cohorts (Figure S5C and D). In the R1 test set and R2 cohort, Bland-Altman^49^ plots show >25-fold deviations in metacluster abundances across samples measured on the Navios and LX (Fig. 3F). All relevant combinations of training and application of FlowSOM clusterings on sample subsets exhibited similar differences (Figure S8).

Together, these results demonstrate substantial variability in marker intensities and cell population estimates across cytometers and over time. Instrument-specific differences were evident in both raw fluorescence signals and derived metacluster abundances. These findings underscore the necessity for methods to correct temporal drift and inter-instrument variability to enable reliable integration of cytometry data.

### Relativisation improves sample alignment and clustering consistency

**Fig. 4:**
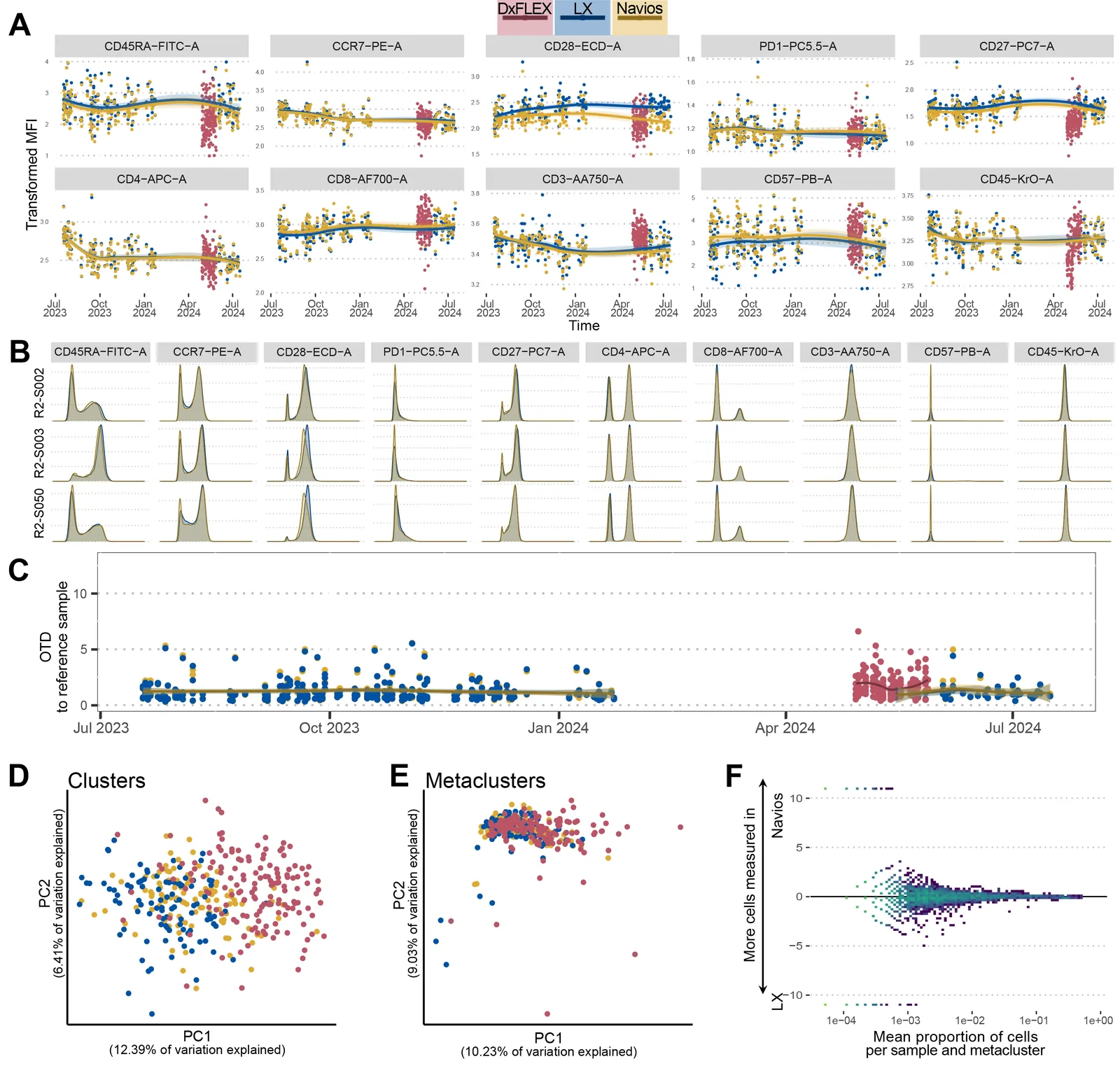
CD3-relativisation improves data alignment between cytometers. **(A)** Longitudinal analysis of surface marker expression in core dataset samples after CD3-relativisation. Compared to the unadjusted data in Fig. 3A, inter-cytometer differences and intra-cytometer variability over time are markedly reduced. **(B)** Density plots of CD3-relativised, arcsinh-transformed fluorescence signals from three representative R2 cohort samples measured with the Navios (yellow) or LX (blue) cytometer. In contrast to Fig. 3B, signals across all channels are closely aligned. **(C)** Global marker-level differences were quantified using optimal transport distance (OTD) to an aggregate reference sample. Comparing with Fig. 3C, CD3-relativisation yields more stable OTD profiles, reflecting improved consistency across cytometers and time points. **(D)** PCA of cell proportions across 400 FlowSOM clusters for all core samples, using the clustering trained on R1-Navios, R1-LX and SAN-DxFLEX samples. Compared to the unadjusted data in Fig. 3D, the separation between cytometers is substantially reduced, demonstrating improved overall alignment of the data. **(E)** PCA of proportions in 98 FlowSOM metaclusters using the same clustering model. In contrast to Fig. 3E, CD3-relativisation eliminates separation between cytometers. **(F)** Bland-Altman plot comparing metacluster proportions in paired R1 and R2 samples measured on Navios and LX cytometers. Compared to Fig. 3F, differences between cytometers are substantially reduced, demonstrating improved cross-cytometer agreement.

After relativisation, inter-cytometer differences (Fig. 3A and B) were practically eliminated in all channels (Fig. 4A and B). Intra-cytometer trends over time was reduced, where signals with minimal variability (e.g., CD3-AA750 and CD45-KrO) remained stable, and more variable signals (e.g., CD28-ECD) were successfully aligned across cytometers (Fig. 4B). Comparable improvements were observed in the FORT (Figure S9A and B) and BAD cohort (Figure S10A and B). Comparing optimal transport distance (OTD) between R1 and R2 samples before (Fig. 3C) and after (Fig. 4C) relativisation demonstrates its overall effectiveness in aligning data within cytometers over time, and between cytometers.

To quantify this improvement, we leveraged the paired design of R1 and R2 cohorts, where each sample was measured on both Navios and LX cytometers. For each sample, we computed the absolute difference in OTD between instruments, |Δ|, as a direct measure of instrument disagreement (Figure S11). Relativisation reduced the median |Δ| by 11.3-fold, from 0.755 (SD 0.39, baseline) to 0.067 (SD 0.087, relativised) (Wilcoxon signed-rank test, p < 0.0001). Therefore, instrument disagreement was consistently reduced across samples.

To further investigate whether cytometers behave differently over time, we fitted a linear mixed-effects model with |Δᵢ| as the outcome, alignment (pre- or post-relativisation) and time (days since first measurement) as fixed effects, and sample identity as a random effect. We confirmed that relativisation reduces instrument disagreement (F = 332, p < 0.001). Neither time (F = 2.75, p = 0.098) nor its interaction with processing (F = 0.84, p = 0.359) was significant, indicating that the improvement by relativisation is stable. Consistent results were obtained when modelling study period (R1 vs R2) instead of time (relativisation: F = 527, p < 0.001; study: F = 2.93, p = 0.088; interaction: F = 0.473, p = 0.49).

We assessed longitudinal drift using 60 donors sampled repeatedly over weeks to months, yielding 168 within-donor, within-instrument sample pairs. A linear mixed-effects model with donor as a random effect confirmed systematic temporal drift in raw measurements: within-donor OTD differences increased significantly with days between measurements (p = 0.033). CD3-relativisation lowered mean pairwise OTD from 0.30 (SD 0.36) to 0.20 (SD 0.19) and produced a strong and significant reduction in within-donor variability (p < 0.001). This benefit was consistent across the full range of sampling intervals (processing × time interaction p = 0.42), meaning that relativised measurements were more similar to other measurements from the same donor regardless of how much time had elapsed between them. Consequently, CD3-relativisation improves temporal comparability in longitudinal studies, yielding more consistent within-donor measurements across sampling windows of weeks to months.

Relativisation allows FlowSOM clustering to more accurately assign similar cells from different samples to the same clusters. This improvement is evident from PCA plots of FlowSOM clusters (Fig. 4D) and metaclusters (Fig. 4E). In particular, R1 and R2 paired samples measured with the LX and Navios were indistinguishable (Figure S12). Similar reductions in sample variance were observed in the FORT and BAD cohorts after relativisation (Figure S9C and S10C).

Comparing Bland-Altman plots of metacluster abundances before (Fig. 3F) and after (Fig. 4F) relativisation shows a marked reduction in deviations between cytometers. Critically, the problem of consistently different, relatively high abundance metaclusters observed in unaligned data was resolved by relativisation. High relative deviations between instruments in low abundance metaclusters remained after relativisation, but this is expected owing to error in sampling from a small number of cells. The improved consistency of metaclusters was evident in all combinations of training and application of FlowSOM clusterings (Figure S12 and S13). Hence, relativisation offers a simple yet effective approach for normalising fluorescence measurements across cytometers and over time.

### T cell distributions predict clinical parameters within cytometers

Our motivation for introducing relativisation was to align clinical flow cytometry measurements across independent laboratories, thereby enabling the direct application of sample-classification models developed at a few sites by many others without exchange of samples or data, and without common reference samples or external calibrators. We do not claim that relativisation is the best possible solution for alignment, only that it is a convenient and readily scalable strategy. In the remainder of this paper, we investigate whether relativisation is an effective method for aligning flow cytometry data when building generalisable predictive models to classify patients.

Our data resource was designed with the intention of supporting the development of generalisable sample-classification methods. A key part of this design was capturing unambiguous donor-associated features that can be predicted from flow cytometry measurements. True sample labels for CMV IgG serostatus, age and biological sex can be accurately determined from clinical records. This is critical because any sample misclassification by models for predicting these features must be errors of the model, not sample mislabelling. In this way, our relativised dataset constitutes a benchmark for testing and fairly comparing sample-classification algorithms using quasi-routine measurements.

**Fig. 5:**
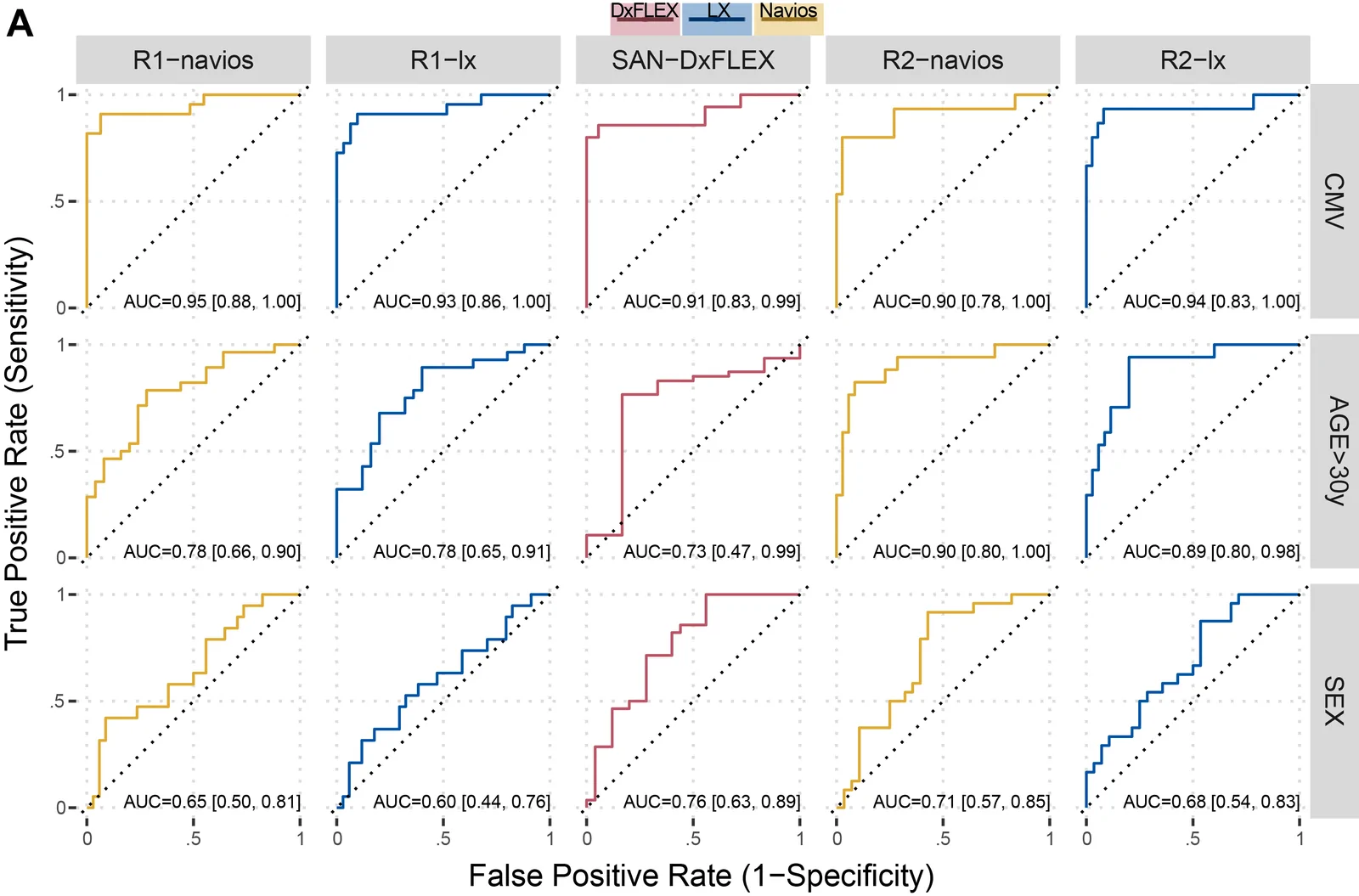
Within cytometer classification performances and cross-cytometer consistency in outcome predictions. ROC curves for classification of CMV-IgG serostatus, age >30 y and sex, based on first-presentation samples from each donor. Random forest models were trained on FlowSOM cluster or metacluster proportions (selected by hyperparameter optimisation) using training samples from the R1 or SAN cohorts separately per cytometer. Models were evaluated on the corresponding held-out test sets. For R2, models trained on R1 data were applied to samples measured on the same cytometer. AUROC values with DeLong confidence intervals are shown.

Adopting a conventional within-cytometer approach, we first clustered with FlowSOM and then trained hyperparameter-optimised^43^ random forests^50^ using cell proportions (Fig. 5). This way, we establish baseline performances for 3 separate models that were created with training-set data from the R1-Navios, R1-LX or SAN cohorts - that is, samples from cohorts R1/R1/SAN measured on the Navios/LX/DxFLEX, respectively. Hyperparameters were validated using validation-set data and final performance was assessed with test-set data from the same instrument. In addition, the prospective performances of our R1-Navios and R1-LX models were tested using R2-Navios and R2-LX data, respectively. These results show that CMV-IgG serostatus, age and sex are predictable from T cell subset distributions when models are trained and evaluated on the same cytometer.

Changes in only a few metaclusters were relevant for predicting CMV-Ig status, most notably CMV seropositivity was associated with expansion of CD57^high^ effector T cells (Figure S14). Other T cell subsets were important for predicting age and sex (Figure S15). Plotting importance values against metacluster sizes revealed that highly informative subpopulations contained 10–100 cells on average (Figure S15). This fits with our expectation that immunological features of latent CMV infection, immunological ageing or biological sex are subtle. Notably, very small metaclusters with high sampling error are not selected by random forest models because they are not consistently informative. Predictions of CMV-IgG^+^ returned AUROCs of 0.90–0.95, so can be taken as a high standard that should, at least, be equalled by future methods. AUROCs for predicting age ranged from 0.73 to 0.90, meaning that predictive models could measurably improve on this moderate performance. Finally, sex was only marginally predictable, with AUROCs ranging from 0.60 to 0.76; therefore, any future improvement in predictive performance might be considered successful. The inclusion of more or less tractable problems in our data resource should help to dissect the strengths and weaknesses of competing approaches.

### Cross-cytometer outcome predictions are systematically biased

Having demonstrated that CMV-IgG serostatus, age and sex are predictable when models are trained and evaluated on the same cytometer, we next asked whether these models transfer to data acquired on different instruments, at different sites and at different time points. Cross-application of models trained on one cytometer to samples acquired on other cytometers led to surprising results (all results in Table S11). Despite marked differences in data alignment between cytometers, CMV-IgG serostatus was highly and consistently predictable across instruments, achieving AUROC values of 0.90–0.95 (Figure S16). Age was also predictable across instruments, with AUROCs between 0.68 and 0.91 (Figure S17); however, performance varied due to differences in age distributions between R1, SAN, and R2 cohorts (Tables S7–S10). Predictions of sex were not consistently significant according to DeLong confidence intervals (Figure S18).

**Fig. 6:**
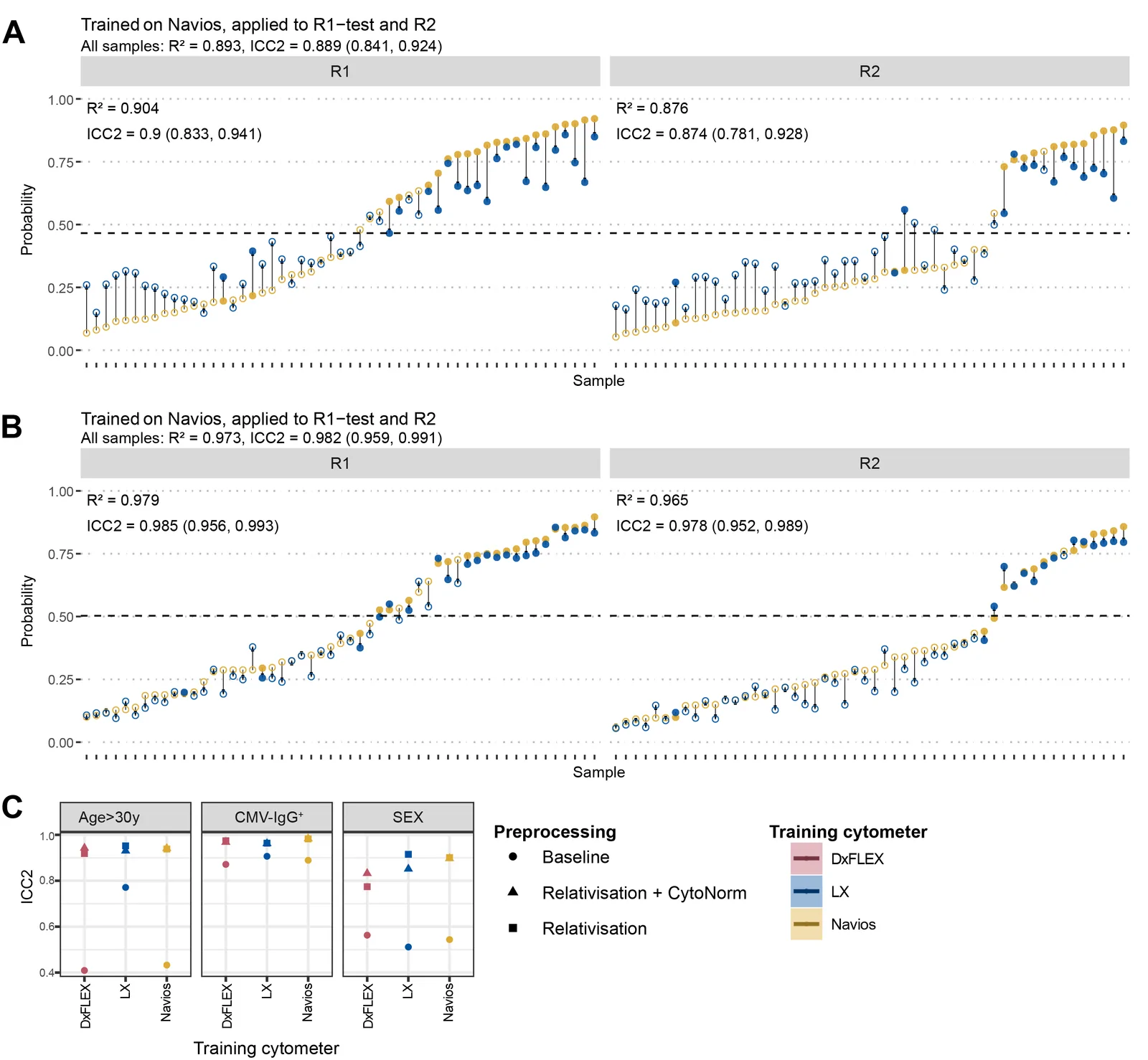
CD3-relativisation improves consistency of predictions across cytometers. **(A–B)** Predicted probabilities of CMV-IgG serostatus from a model trained on R1-Navios data, applied to the test set from R1-Navios and R1-LX, and to R2-Navios and R2-LX samples. Either (A) unrelativised or (B) relativised data were used. Each point represents a sample, with colour denoting measurements with a Navios (yellow) or LX (blue) cytometer. Filled circles indicate CMV-IgG^+^ samples, whereas open circles indicate CMV-IgG^−^ samples. The dashed line indicates the classification threshold based on the ROC curve (minimum distance to the top-left corner) from validation set R1-Navios data. Relativisation markedly improved prediction consistency across cytometers. **(C)** Agreement between Navios and LX cytometer predictions, quantified by intraclass correlation coefficients (ICC2; two-way random effects, absolute agreement, single rater). Each panel shows ICC2 values for models trained to predict CMV-IgG serostatus, age >30 y, or sex on data from either R1-Navios, R1-LX or SAN-DxFLEX (x-axis and colour). Models were evaluated on paired Navios and LX measurements of samples not used for training. Shapes indicate the preprocessing strategy: Baseline, CD3-relativisation or CD3-relativisation followed by CytoNorm. CD3-relativisation consistently improves cross-cytometer agreement across all tasks, while additional CytoNorm normalisation does not provide further benefit.

Importantly, although overall performance was unexpectedly reliable in predicting CMV-IgG serostatus, applying models across cytometers revealed systematic biases (Fig. 6A; Figures S19–S21). We observed that “intra-instrument” predictions were more confident than “inter-instrument” predictions. This is illustrated in Fig. 6A, where a model trained with R1-Navios data was applied to R1-Navios, R1-LX, R2-Navios and R2-LX. Predictions within the training instrument (yellow points, Navios) were typically more extreme than those made across instruments (blue points, LX), owing to reduced confidence and systematic bias when transferring models between platforms. We quantified prediction consistency with the intra-class correlation coefficient (ICC2)^44^ across all models and outcomes. For CMV-IgG^+^ status, ICC2 values for R1 test and R2 samples were 0.889 (95% CI: 0.841–0.924) using the R1-Navios model, 0.907 (0.761–0.954) with the R1-LX model, and 0.871 (0.169–0.958) with the SAN-DxFLEX model (Figure S19). Predictions for age and sex were markedly less consistent (Figure S20 and S21). Comparable ICC2 values emerged in the FORT and BAD cohorts, where matched samples were measured on LSR, Navios, LX, and CA cytometers (Figures S22 and S23). When models trained on R1-Navios and R1-LX were applied to samples acquired on LSR or CA cytometers, consistency ranged from high agreement for CMV-IgG serostatus (ICC2 = 0.9) to low agreement for age and sex (ICC2 = 0–0.09, Figure S23).

These results further underscore the value of our datasets by highlighting the challenges of cross-platform generalisation. Our data serve as a resource for assessing the robustness and transferability of predictive models across uncalibrated cytometry platforms. Specifically, using paired samples and ICC2, we established an interpretable framework for evaluating model generalisability.

### Relativisation restores predictive consistency across cytometers

Next, we asked whether the improved consistency of FlowSOM clustering after relativisation also improved the quality of clinical predictions made with hyperparameter-optimised random forest models. As for the unaligned datasets, separate models were generated with relativised data from R1-Navios, R1-LX, or SAN-DxFLEX. Predictive performances for CMV-IgG^+^ status, age and sex were then assessed within and between cytometers.

After relativisation, predictions of CMV-IgG^+^ remained highly accurate, with AUROCs ranging from 0.90 to 0.93 (Figure S16). Predictions of age using relativised data also showed robust performance with AUROCs between 0.75 and 0.93 (Figure S17). Predictions of sex remained weak and not consistently significant as indicated by DeLong confidence intervals (Figure S18). Hence, relativisation preserved the strong predictive performance for CMV-IgG and age while sex remained difficult to predict.

Critically, comparing class membership probabilities before (Fig. 6A) and after (Fig. 6B) relativisation revealed far greater consistency in predictions across instruments when the data were aligned. Quantifying agreement between paired samples from R1 and R2, each measured on the Navios and LX, revealed consistent improvement in ICC2 after relativisation: we obtained highly consistent predictions for CMV-IgG serostatus (ICC2≥0.964) and age (ICC2≥0.919) and also to some extent for sex (ICC2≥0.774) (Fig. 6C, Figures S24–S26). In the FORT cohort, consistency likewise improved across matched samples measured on LSR, Navios, and LX (ICC2≥0.919, Figure S27). Moreover, when CMV-IgG models trained on R1-Navios and R1-LX data were applied to samples measured on the conventional LSR and spectral CA, prediction agreement increased to excellent levels (ICC2 > 0.99, Figure S28).

Together, these results demonstrate that relativisation not only improves data alignment and FlowSOM clustering consistency, but also substantially enhances the reliability and cross-platform generalisability of clinical predictions.

### Relativisation outperforms CytoNorm

A recent publication introduced CytoNorm2.0, which enables alignment of cytometry data without requiring technical replicates or control samples.^33^ In essence, CytoNorm2.0 aggregates all samples within separate batches into single samples, then aligns samples by quantile normalisation within FlowSOM metaclusters. To evaluate its performance in aligning fully independent datasets, we applied CytoNorm2.0 to training samples from the R1 cohort, treating R1-Navios and R1-LX as unpaired samples and the cytometers as batches. This approach failed to properly align the data because the initial differences between instruments were too extreme (Figure S29). Meaningful alignment was observed only when the number of metaclusters was reduced to the artificially low value of three. However, this reduced the method to a near-trivial quantile normalisation, as over 93% of cells were assigned to a single metacluster. Increasing the number of metaclusters beyond four led to complete separation of cells in different metaclusters by cytometer, violating a key requirement of CytoNorm that metaclustering is minimally affected by technical variation between batches (compare Fig. 3D and E with Figure S30).

Recognising that CytoNorm cannot replace relativisation in aligning very divergent datasets, we next asked whether it could refine alignment after relativisation. Because relativisation already minimises metacluster distortion (Fig. 4E), CytoNorm's assumptions are better met in this context. Therefore, we applied CytoNorm2.0 to relativised training samples from R1 and used it to normalise all samples in R1, R2, and SAN. Visually, alignment neither improved nor deteriorated (Figure S31). The consistency of predicted class membership probabilities showed mixed results: when trained on SAN-DxFLEX, agreement improved for age (ICC2: 0.943 vs. 0.919) and sex (0.832 vs. 0.774), but when trained on R1-LX, performance declined (age: 0.930 vs. 0.952; sex: 0.852 vs. 0.915) (Fig. 6C; Figures S32–S34).

### Relativisation improves predictive consistency across patient cohorts

Technical robustness is necessary for generalising diagnostic methods; however, predictive models can only be generalised if they are also biologically robust. To confirm that models for CMV-IgG status, sex and age can be successfully applied to grossly dissimilar patient populations, we collected a further 100 samples from patients admitted to ICU within 24 h of major surgery. These patients present a greater challenge than the healthy donors used for model training because acute surgical stress profoundly alters peripheral blood T cell distributions.^51^

**Fig. 7:**
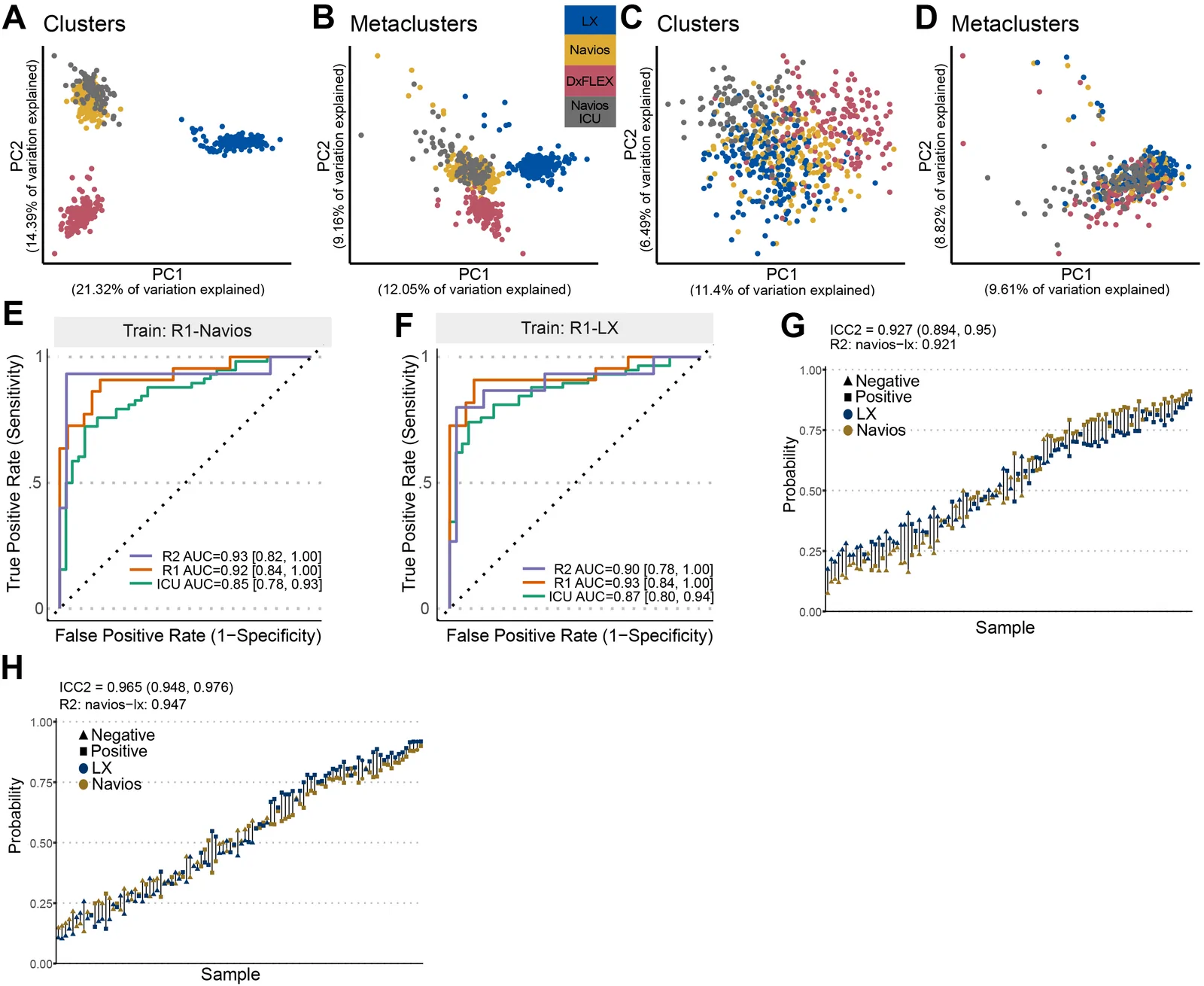
Relativisation improves predictive performance and consistency across dissimilar patient cohorts. **(A–B)** PCA plots of R1, R2, SAN and ICU samples based upon FlowSOM (A) cluster or (B) metacluster frequencies using arcsinh-transformed unrelativised data. Each point represents a sample, colour-coded by cytometer. **(C–D)** PCA plots of R1, R2, SAN and ICU samples based upon FlowSOM (C) cluster or (D) metacluster frequencies using arcsinh-transformed relativised data. **(E)** Prediction of CMV-IgG serostatus in R1-Navios-test, R2 or surgical ICU sets using a classifier trained on R1-Navios-train/validation data. **(F)** Prediction of CMV-IgG serostatus in R1-Navios-test, R2 or surgical ICU sets using a classifier trained on R1-LX-train/validation data. **(G–H)** Predictions of CMV-IgG serostatus when trained on R1-Navios-train/validation samples before (G) or after (H) relativisation.

We first assessed data alignment. Prior to relativisation, PCA of FlowSOM cluster and metacluster frequencies showed clear separation of ICU samples by instrument (Fig. 7A and B). Relativisation largely eliminated these differences (Fig. 7C and D), whereas CytoNorm2.0 alone did not reliably correct inter-cytometer shifts (Figure S35).

We then applied the CMV-IgG serostatus classifiers trained on R1-Navios or R1-LX data (which were already applied to the R1-Navios test set and R2-Navios set) to the ICU cohort (Fig. 7E and F). ICU cohort demographics are shown in Table S12. Both models performed similarly well across all three test sets. Importantly, model agreement improved markedly after relativisation (ICC2 = 0.965) compared to before (ICC2 = 0.927) (Fig. 7G and H). Results for age and sex are provided as Supplementary Materials (Figure S36 and S37). These findings demonstrate that relativisation is a suitable method for aligning flow cytometry data across clinically divergent cohorts and that it substantially stabilises downstream predictive performance across instruments and very different study populations.

## Discussion

If we are to advance flow cytometry-based clinical decision-making from an operator-dependent process to a scalable clinical technology, we must fundamentally rethink how analysis pipelines are constructed and evaluated. Ultimately, our goal is to make reliable sample-level predictions. Such predictions must be accurate and interpretable, and they must hold for the next patient, processed in the next hospital, on the next instrument, by the next technician, and within a clinically actionable timeframe.

To support this development, we assembled a data resource comprising samples collected across four laboratories using six instruments over a 20-month period, organised into retrospective exploration and prospective validation cohorts. In contrast to existing benchmark datasets from the FlowCAP challenges,^52^^,^^53^ PhenoGraph,^54^ Cytofln^32^ or others,^23^^,^^55^^,^^56^ our dataset incorporates quasi-routine measurements of fresh-blood samples over an extended period, across different countries and as technical replicates on multiple cytometers. This design enables rigorous assessment of a pipeline's ability to generalise beyond the conditions under which it was developed.

Our study demonstrates that making clinical predictions from flow cytometry analysis of peripheral blood leucocytes is feasible, even across heterogeneous datasets and clinically diverse patient cohorts. CMV-IgG^+^ status was predicted with high accuracy across cohorts and cytometers, reflecting its strong immunological imprint; hence, it serves as a reliable benchmark for minimum acceptable predictive performance of any algorithm. Our performance in prospective prediction of age was moderate (AUROC = 0.90 [0.80–1.00]), whereas our ability to predict sex of donors is a current limitation (AUROC = 0.71 [0.57–0.85]), even after relativisation. Based on these results, we cannot say whether information about age or sex is only weakly encoded in T cell distributions,^57^ whether our T cell panel lacked critical markers of donor age-related changes in T cells, or whether our modelling methods failed to capture information present in our data.

Variability in clinical flow cytometry measurements is usually controlled with bench-level calibration methods, such as BC Flow Set Pro or BD CS&T beads.^58^ However, even using these strategies, manual gating adjustments or post-hoc computation are usually necessary to ensure inter-lab comparability.^59^ Our findings demonstrate that relativisation is a practical and effective alternative: By aligning measurements against an internal reference feature, relativisation requires no external calibration, preserves biological signals and enables robust analyses.

Hence, we propose CD3-based relativisation as an easily implemented step in the design and analysis of flow cytometry studies, especially in longitudinal or multi-centre studies, or when integrating data from different instruments. While we acknowledge that properly maintaining instruments and daily instrument performance checks are best practice, our simple procedure enables reliable alignment without the need for shared reference samples or elaborate calibration efforts. With this approach, we showed that data from international labs, instruments from different manufacturers and detector technologies can be well-enough aligned to generate a single, harmonised dataset from multiple sources.

Compared to established normalisation algorithms such as CytoNorm and FlowStats, our method offers a distinct advantage in both simplicity and adaptability. While FlowStats is designed specifically for flow cytometry, it depends on paired samples or calibration beads, limiting its use in the next, unseen sample of unknown origin.^60^ CytoNorm was originally introduced as a way to correct batch errors using common control samples from each batch,^31^ but was recently extended as CytoNorm 2.0 to support alignment without dedicated controls.^33^ Pushed beyond its intended purpose in our study, CytoNorm2.0 underperformed owing to high variability between instruments and sites. Applying CytoNorm2.0 after relativisation yielded no benefit over relativisation alone.

Relativisation and its associated analysis pipeline still include steps that must be refined in further studies. Despite its advantages, relativisation depends on the presence of a stable internal reference feature: here, we used the amount of CD3 on the surface of T cells. The choice of CD3 was based upon its relative stability in our study population, as well as the convenience of using a well-characterised antibody (UCHT1) that was commercially available in all required conjugates. Although this requirement is far less restrictive than needing paired samples or calibration materials, it may pose limitations in experiments where such populations are absent by design. In such cases, we could imagine using other stable markers or composite “housekeeper” scores from multiple markers.

In our current analysis pipeline, the parameters for the arcsinh transformation are selected manually, but this could be automated on a per-sample basis in future.^61^ Similarly, although FlowSOM clustering followed by random forest classification has proved versatile and effective, numerous alternative approaches exist that merit exploration.^52^^,^^62^, ^63^, ^64^, ^65^, ^66^, ^67^ The data resource presented here provides an objective, real-world benchmark for systematically evaluating clustering and classification methods in flow cytometry.

CD3-relativisation is important because it allows us to compare data that were not measured in precisely the same way: it circumvents the need for sophisticated technical controls that are most easily implemented by dedicated central labs. Hence, CD3-relativisation facilitates out-scaling and generalisation of predictive models, which are crucial for point-of-care applications of flow cytometry. In future, we envisage placing flow cytometers into outpatient clinics or wards, so that flow cytometry could contribute to real-time clinical decision-making; for instance, in the rapid evaluation of patients presenting to the Emergency department or ICU with surgical complications or traumatic injuries.^68^^,^^69^

Our benchmark data resource sets robust standards for multi-centre flow cytometry analysis. By eliminating the need for paired controls, our approach unlocks scalable, automated pipelines for both high-throughput biomarker discovery and clinical applications. We envisage its use in multi-centre, longitudinal immunomonitoring trials and diagnostic workflows—settings where reproducibility, generalisability, technical simplicity and speed are essential.

## Contributors

GG and JAH designed and performed the project and wrote the manuscript. KK, PR, and TS performed experiments and analysed data. GG, JAH, KK, and PR directly accessed and verified the underlying data reported in the manuscript. All authors had access to all the data in the study. FA, DS, MIE, MX, and HV performed experiments. MK and GB gave expert cytometry advice. VH and TD provided clinical data. VJLM and RS gave expert computational and statistical advice. HJS, EKG, SG, PB, EMC, and MLH provided clinical samples and infrastructure. All authors reviewed the article and consented to its publication.

## Data sharing statement

The authors declare that all data supporting the findings of this study are available within the paper, its Supplementary Information Files (10.5281/zenodo.17120077) and downloadable files deposited at Zenodo (Main resource 10.5281/zenodo.17094078, ICU resource 10.5281/zenodo.17700440).^42^

The authors declare that all computer code supporting the findings of this study is available as Supplementary Information Files and downloadable files deposited at https://github.com/ggrlab/2025-Relativisation.

## Declaration of interests

MK is an employee of Beckman Coulter Life Sciences GmbH, a company that manufactures laboratory instruments and reagents. All other authors have no competing interests to declare.
