# Supplementary material for: Superior precision of clinical predictions after CD3-relativisation to align flow cytometry data
Source: eBioMedicine. 2026 Aug 12;131:106427. doi: 10.1016/j.ebiom.2026.106427 (PMC13488037; doi:10.1016/j.ebiom.2026.106427)
Supplement: Supplementary file 1 [file mmc1.pdf]

–SUPPLEMENTARY INFORMATION–

Superior Precision of Clinical Predictions after CD3-relativisation to Align Flow Cytometry Data

Gunther Glehr[1], Katharina Kronenberg[1], Fabiola Arella[1], Michael Kapinsky[1,2], David Segundo[3], María Iglesias-Escudero[4], Maria Xydia[5,6], Hendrik Veltman[4], Tobias Schmidt[1], Viola Hähnel[7], Víctor J. López-Madrona[8], Thomas Dienemann[1], Hans J. Schlitt[1], Edward K. Geissler[1], Gero Brockhoff[9], Silvia Gregori[10], Philipp Beckhove[5,11], Rainer Spang[12], Eva Martínez Cáceres[4], Marcos Lopez Hoyos[3], Paloma Riquelme[1], and James A. Hutchinson[1,6,*]

[1]Department of Surgery, University Hospital Regensburg, Regensburg, Germany
[2]Beckman Coulter Life Sciences GmbH, Krefeld, Germany
[3]Division of Immunology, Hospital Universitario Marqués de Valdecilla, IDIVAL, Santander, Cantabria, Spain
[4]Immunology Division, LCMN, Germans Trias i Pujol University Hospital and Research Institute, Campus Can Ruti, Badalona, Spain
[5]Division Interventional Immunology, Leibniz Institute for Immunotherapy, Regensburg, Germany
[6]Bavarian Cancer Research Center (BZKF), Regensburg, Germany
[7]Department of Transfusion Medicine, University Hospital Regensburg, Regensburg, Germany
[8]Institute for Microbiology and Hygiene, University Hospital Regensburg, Regensburg, Germany
[9]Aix Marseille Univ, INSERM, INS, Inst Neurosci Syst, Marseille, France
[10]Department of Gynecology and Obstetrics, University Medical Center Regensburg, Germany
[11]San Raffaele Telethon Institute for Gene Therapy (SR-TIGET), IRCCS San Raffaele Scientific Institute, Milan, Italy
[12]Department of Statistical Bioinformatics, University of Regensburg, Regensburg, Germany
[*]Corresponding author: James A. Hutchinson, james.hutchinson@ukr.de

[*]James A. Hutchinson, james.hutchinson@ukr.de

Supplementary Notes

Supplementary Note 1: Cohort description S4

Supplementary Note 2: Clinical investigations S5

Supplementary Note 3: Optimal transport distances S6

Supplementary References S7

Supplementary Figures

1 Manual gating strategy for DURAClone IM T Cell Subsets tube S9
2 Predictive performance at different numbers of clusters S10
3 CD3 relativisation visualised for all channels S11
4 Unadjusted samples measured on Navios, CytoFLEX and BD LSRFortessa. S12
5 Within- and between-device variability comparing flow and spectral cytometry. . . S13
6 Median fluorescence intensities of single stained samples over time S15
7 Median fluorescence intensities of single stained samples over time in the FORT cohort. S17
8 Bland-Altman plots for paired samples on Navios and LX across differently trained FlowSOMs S19
9 CD3 relativised data from samples measured on Navios, CytoFLEX and BD LSRFortessa. S21
10 Within- and between- device variability comparing flow and spectral cytometry after CD3 relativisation S23
11 Quantification of instrument agreement using paired optimal transport distances. . S25
12 PCA plots of cluster and metacluster proportions after relativisation S26
13 Bland-Altman plots for paired Navios and LX samples across FlowSOM models after relativisation S28
14 Phenotypic characterization of important CMV serostatus metaclusters 98 and 85 . S30
15 Feature importance vs. average cluster size S31
16 ROC curves for CMV-IgG seropositivity classification across devices after relativisation S32
17 ROC curves for age across different devices post relativisation S33
18 ROC curves for sex across different devices post relativisation S34
19 Predicted probabilities of CMV seropositivity across cytometers S35
20 Predicted probabilities of age above 30 based on metacluster cell proportions S37
21 Predicted probabilities of sex based on metacluster cell proportions S39
22 Predicted probabilities of age, sex and CMV-IgG seropositivity in the FORT cohort . S41
23 Predicted probabilities of age, sex and CMV-IgG seropositivity in the BAD cohort . S43
24 Predicted probabilities of CMV-IgG seropositivity after relativisation S45
25 Predicted probabilities of age above 30 after relativisation S47
26 Predicted probabilities of sex after relativisation S49

27 Predicted probabilities of age, sex and CMV-IgG serostatus on FORT cohort after relativisation. S51
28 Predicted probabilities of age, sex and CMV-IgG seropositivity in the BAD cohort after relativisation. S53
29 Performance of CytoNorm v2.0 under strong batch effects. S55
30 PCA plots coloured by R1-Navios, R1-LX, R2-Navios, R2-LX S57
31 Cytonorm after CD3 relativisation does not improve alignment over relativisation alone. S59
32 Predicted probabilities of CMV-IgG seropositivity after relativisation and CytoNorm S61
33 Predicted probabilities of age above 30 after relativisation and CytoNorm S63
34 Predicted probabilities of sex after relativisation and CytoNorm S65
35 Performance of CytoNorm v2.0 under strong batch effects with the ICU cohort. . . S67
36 AUROC curves for age, sex and CMV-IgG seropositivity in the ICU cohort S69
37 Predicted probabilities of age, sex and CMV-IgG seropositivity in the ICU cohort . . S70

Supplementary Tables

1 DuraClone T cell subsets Tube S71
2 Single stain tube concentrations. S72
3 Cofactors before and after rescaling for all cytometers. Cofactors were determined on panel samples by a blinded, experienced cytometrist. S73
4 Definitions of fields in file naming structure. S74
5 Sample stainings and their definitions. S74
6 Label descriptions of the data. S75
7 Demographic information for R1 training, validation and test sets. S76
8 Demographic information for SAN, R2 and R1 (training, validation and test). No haemogram was performed for SAN samples. S78
9 Demographic information for SAN split into training, validation and test sets. S79
10 Demographic information for FORT and spectral (BAD) cohort cohort. S79
11 Performances of all tested combinations of classifiers and test sets. S80
12 Demographic information for 95 unique patients of the ICU cohort. 5 patients were presented twice resulting into 100 total samples measured. For 2 samples no serum was conserved, therefore CMV-IgG serostatus (CMV_AB) is missing. S81

Supplementary Note 1: Cohort description

The R1 dataset consists of 254 samples from 153 donors, some of whom contributed up to six times. Samples were collected between 18 July 2023 and 23 January 2024, and randomly assigned to training (n = 50), validation (n = 50), and test (n = 53) sets. Clinical variables did not differ significantly across these subsets (Supplementary Table 2). This dataset serves two primary roles: (1) split into training, validation and test to train models to be applied to the prospective R2 or external SAN datasets, or (2) as an external test set when models are trained exclusively on SAN samples. Since all R1 samples were measured on both the Navios™ and CytoFLEX LX™ cytometers, the dataset is also well suited for evaluating cross-cytometer performance under tightly controlled conditions.

The R2 dataset comprises 52 samples from 52 donors, measured between 15-MAY-2024 and 16-JUL-2024. This dataset is used exclusively as prospective test data and is never included in training. Measured on the same cytometers as the R1 dataset, R2 enables evaluation of model generalization to new data acquired six months later.

The SAN dataset includes 153 samples from 153 donors, measured between 29-APR-2024 and 28-MAY-2024 on a DxFLEX cytometer. Its primary role is to assess the real-world performance of models trained on R1, by introducing variation in location, time, and instrument. Alternatively, SAN has been split into training (n = 50), validation (n = 50), and test (n = 53) subsets to reverse the analytical setting: Train models on SAN and test them on R1 and R2.

The FORT dataset includes 14 samples from 14 donors, measured between 14-JAN-2025 and 24-JAN-2025. The dataset is used prospectively to compare analyses between Navios™, CytoFLEX LX™, and LSRFortessa™cytometers. Several donors were previously sampled in the R1 or R2 cohorts. This dataset supports prospective comparison across these instruments under controlled conditions.

The BAD dataset includes 9 samples measured from 9 donors, measured between 18-MAR-2025 and 19-MAR-2025. The dataset is used prospectively to compare analyses between conventional (LSRFortessa™) and spectral (Cytek Aurora™) cytometers.

The ICU dataset consists of 100 samples from 95 donors, some of whom contributed up to two times. Samples were collected between 01-OCT-2024 and 09-MAY-2025 from post-surgery patients in the intensive care unit (ICU) of the University Hospital Regensburg. This cohort was measured on both the Navios™ cytometer, making it suitable for evaluating model generalization to a different clinical setting and patient population, as well as for assessing CytoNorm performance under strong batch effects between the Navios™ and CytoFLEX LX™ cytometers.

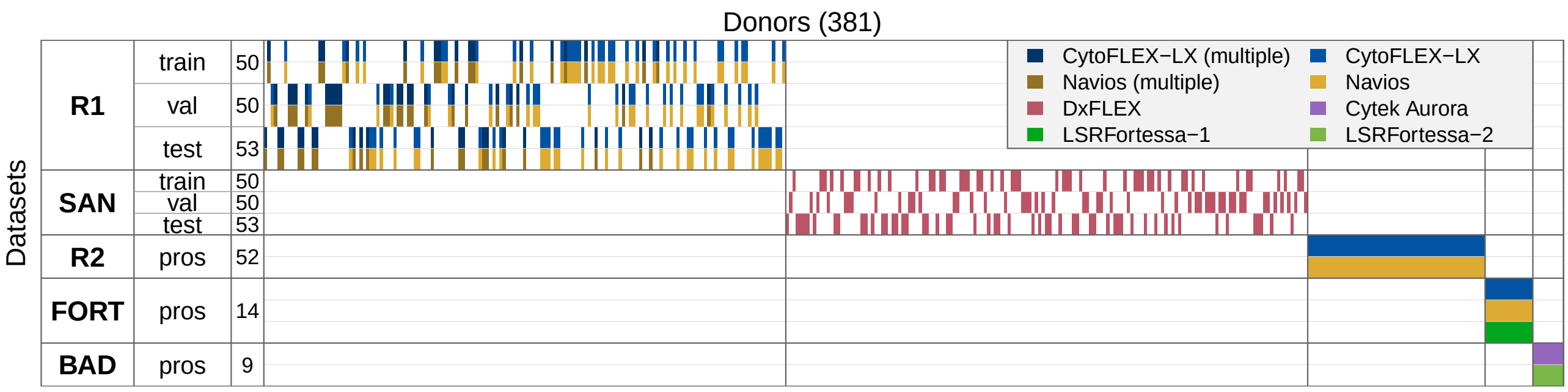

Note 1, Figure 1: *Heatmap of data resource donor coverage across studies (R1, SAN, R2, FORT, BAD) and their respective data splits.*

Each column represents an individual donor and each row corresponds to a study-specific subset (training, validation, test, or prospective) and multiple cytometers. Cell colors indicate the cytometer used for measurement. With the exception of SAN, all samples were stained once, then split and measured on at least two cytometers. Donors who provided independent blood draws across multiple studies are shown only at their first appearance and marked in a darker shade. Note that those are true biological replicates rather than repeated measurements of a stored sample. Numbers alongside each subset denote the number of donors per dataset split, illustrating cohort sizes and their distribution across studies. Within R1 and SAN, samples were randomly assigned to training, validation, and test sets.

Supplementary Note 2: Clinical investigations

A comprehensive haemogram and infectious disease screening were conducted to routinely evaluate various hematological and virological parameters for all samples measured in Regensburg. The parameters and their abbreviations are detailed in Supplementary Table 6, while the summary statistics of the R1, R2, and SAN cohorts are provided in Supplementary Table 7-9. We report ABO blood group, Rhesus (D) status, Kell status and irrelgular blood group antibody screen results. Hematological parameters included total leucocyte count (c/nl), erythrocyte count (c/pl), haemoglobin concentration (g/dl), haematocrit (%), mean corpuscular volume (fl), mean corpuscular haemoglobin (pg), mean corpuscular haemoglobin concentration (g/dl), thrombocyte count (c/nl), and red cell distribution width (both standard deviation in fl and coefficient of variation in %). Additionally, thrombocyte distribution width (fl), mean thrombocyte volume (fl), and the distribution of various white blood cell types—neutrophils, lymphocytes, monocytes, eosinophils, basophils, immature granulocytes, and erythroblasts—were quantified both as percentages and absolute counts (c/nl or per 100 WBC/RBC).

Apheresate samples were analyzed to determine counts for leucocytes, erythrocytes, reticulocytes, thrombocytes (by optical and impedance methods), and associated parameters such as haemoglobin concentration, haematocrit, mean corpuscular volume, mean corpuscular haemoglobin, and mean corpuscular haemoglobin concentration.

Virological screening was performed using PCR assays for Hepatitis B Virus (HBV), Hepatitis C Virus (HCV), Hepatitis E Virus (HEV), Human Immunodeficiency Virus (HIV-1/2), and West Nile Virus (WNV). Serological testing included anti-A, anti-B, anti-Treponema pallidum, Hepatitis B surface antigen, anti-HBV core antigen, anti-HCV, anti-HIV-1/2, p24 antigen, and anti-Cytomegalovirus (CMV) IgG.

Supplementary Note 3: Optimal transport distances from a Mathematical Point of View

Here we provide the mathematical foundations for the Optimal Transport Distance (OTD) metric described in the Methods section.

We now explain OTD in the context of flow cytometry, following the structure of Feydy[1]. We introduce OT as a one-dimensional sorting, extend to multi-dimensional sorting, describe the Wasserstein-2 (-p) distance, transport plans, the Sinkhorn algorithm, and finally the (de-biased) Sinkhorn divergence which the python package `geomloss` provides. We ultimately use OTD simply as a measure of dissimilarity between two samples of n and m cells with p markers ($X \in \mathbb{R}^{n \times p}$, $Y \in \mathbb{R}^{m \times p}$) leading to a single number $\mathrm{OT}(X, Y) \in \mathbb{R}_{\geq 0}$.

Consider two measured flow cytometry samples $X \in \mathbb{R}^{n \times p}$ and $Y \in \mathbb{R}^{m \times p}$ of n and m cells/events with p measured parameters per cell. Let α and β be weighted cell clouds which are effectively discrete measures:

$$\alpha = \frac{1}{n} \sum_{i=1}^{n} \mathbf{a}_i \, \delta_{\mathbf{x}_i}, \tag{1}$$

$$\beta = \frac{1}{m} \sum_{j=1}^{m} \mathbf{b}_j \, \delta_{\mathbf{y}_j}, \tag{2}$$

where $\delta_{\mathbf{x}_i}$ is a Dirac distribution fully concentrated at a single cell $\mathbf{x}_i$ or $\mathbf{y}_j \in \mathbb{R}^p$ and cell-weights $\mathbf{a} \in \mathbb{R}^n_{\geq 0}$, $weightb \in \mathbb{R}^m_{\geq 0}$. The weights are commonly normalised such that $\sum_i \mathbf{a}_i = \sum_j \mathbf{b}_j = 1$.

In the simplified case of equal cell numbers with one dimension ($m = n$, $p = 1$), we can define the optimal transport distance between two cell clouds α, β as reordering the cells in Y to match the order of cells in X. Assume reordered x_i such that

$$x_1 \leq x_2 \leq \cdots \leq x_n, \tag{3}$$

then we find the "optimal" permutation $\sigma : \{1, \ldots, n\} \longrightarrow \{1, \ldots, n\}$ such that

$$y_{\sigma(1)} \leq y_{\sigma(2)} \leq \cdots \leq y_{\sigma(n)}. \tag{4}$$

If we use weighted cell clouds, $\mathbf{a}_i = \mathbf{b}_i = 1/n$ for all i, the transport distance according to the Wasserstein-2 loss using the "optimal" permutation becomes

$$\mathrm{OT}(\alpha, \beta) \coloneqq \frac{1}{2n} \sum_{i=1}^{n} \left(x_i - y_{\sigma(i)}\right)^2, \tag{5}$$

which can now be extended to higher dimensions[2] ($p > 1$) with

$$\mathrm{OT}(\alpha, \beta) = \min_{\sigma} \frac{1}{2n} \sum_{i=1}^{n} \left\| \mathbf{x}_i - \mathbf{y}_{\sigma(i)} \right\|^2. \tag{6}$$

Further generalizing to any pair of discrete positive measures[3] α, β for n and m cells with p markers leads to the optimal transport distance defined as

$$\mathrm{OT}_C(\alpha, \beta) \coloneqq \min_{\boldsymbol{\pi}_{ij}} \sum_{i=1}^{n} \sum_{j=1}^{m} \boldsymbol{\pi}_{ij} \, \mathbf{C}_{ij} \tag{7}$$

subject to

$$\forall\, i, j:\ \boldsymbol{\pi}_{ij} \geq 0, \qquad \sum_{j=1}^{m} \boldsymbol{\pi}_{ij} = a_i, \qquad \sum_{i=1}^{n} \boldsymbol{\pi}_{ij} = b_j, \tag{8}$$

given a cost function, e.g. a pre-defined cost matrix or the squared Euclidean distance:

$$C : \mathbb{R}^p \times \mathbb{R}^p \longrightarrow \mathbb{R}_{\geq 0}, \qquad (\mathbf{x}, \mathbf{y}) \mapsto \|\mathbf{x} - \mathbf{y}\|^2, \qquad \mathbf{C}_{ij} \coloneqq C(\mathbf{x}_i, \mathbf{y}_j) = \|\mathbf{x}_i - \mathbf{y}_j\|^2, \tag{9}$$

and the aim to find the optimal transport plan $\boldsymbol{\pi}_{ij} \in \mathbb{R}_{\geq 0}^{n \times m}$. The element $\boldsymbol{\pi}_{ij}$ can be interpreted as the effort/cost necessary to transport cell i at position $\mathbf{x}_i$ to cell j at position $\mathbf{y}_j$. The transport plan is optimal when the costs to transfer all cells in X to Y are minimal. The rows of $\boldsymbol{\pi}$ sum to $\mathbf{a}_{row}$ and the columns sum to $\mathbf{b}_{column}$.

Practically, this optimal transport distance is often infeasible to calculate due to the combinatorial nature of the problem. We can use entropic regularization to obtain an approximate solution:

$$\mathrm{OT}_C^{\varepsilon}(\alpha, \beta) \coloneqq \min_{\boldsymbol{\pi}} \left\{ \sum_{i=1}^{n} \sum_{j=1}^{m} \boldsymbol{\pi}_{ij}\, \mathbf{C}_{ij} - \varepsilon\, H(\boldsymbol{\pi}) \right\} \tag{10}$$

with

$$H(\boldsymbol{\pi}) \coloneqq - \sum_{i=1}^{n} \sum_{j=1}^{m} \boldsymbol{\pi}_{ij} \left(\log\left(\boldsymbol{\pi}_{ij}\right) - 1 \right) \tag{11}$$

and $H(\boldsymbol{\pi}) \coloneqq -\infty$ if any $\boldsymbol{\pi}_{ij} \leq 0$. This can now be solved robustly and efficiently using the Sinkhorn algorithm[4].

Unfortunately, the added entropic bias leads to $\mathrm{OT}_C^{\varepsilon}(\alpha, \alpha) \neq 0$, which then allows minimized, degenerate cases of $\mathrm{OT}_C^{\varepsilon}(\alpha, \beta) < \mathrm{OT}_C^{\varepsilon}(\beta, \beta) \neq 0$. To counteract this, we can use the de-biased Sinkhorn divergence[5]:

$$S_C^{\varepsilon}(\alpha, \beta) \coloneqq \mathrm{OT}_C^{\varepsilon}(\alpha, \beta) - \frac{1}{2} \left[\mathrm{OT}_C^{\varepsilon}(\alpha, \alpha) + \mathrm{OT}_C^{\varepsilon}(\beta, \beta) \right]. \tag{12}$$

This divergence satisfies $S_C^{\varepsilon}(\alpha, \beta) \geq 0$ and $S_C^{\varepsilon}(\alpha, \alpha) = 0$. The parameter ε controls the trade-off between higher accuracy of the optimal transport distance approximation ($\varepsilon \longrightarrow 0$) and computational efficiency ($\varepsilon \longrightarrow \infty$).

References

1.Feydy, J. Geometric Data Analysis, beyond Convolutions. *Applied Mathematics* (2020).

2.Monge, G. Mémoire Sur La Théorie Des Déblais et Des Remblais. *Mem. Math. Phys. Acad. Royale Sci.,* 666–704 (1781).

3.Kantorovich, L. V. *On the Translocation of Masses* in *Dokl. Akad. Nauk. USSR (NS)* **37** (1942), 199–201.

4.Cuturi, M. *Sinkhorn Distances: Lightspeed Computation of Optimal Transport* in *Proceedings of the 27th International Conference on Neural Information Processing Systems - Volume 2* **2** (Curran Associates Inc., Red Hook, NY, USA, 2013), 2292–2300.

5.Ramdas, A., Trillos, N. G. & Cuturi, M. On Wasserstein Two-Sample Testing and Related Families of Nonparametric Tests. *Entropy* **19,** 47. ISSN: 1099-4300 (2017).

Supplementary Figure 1

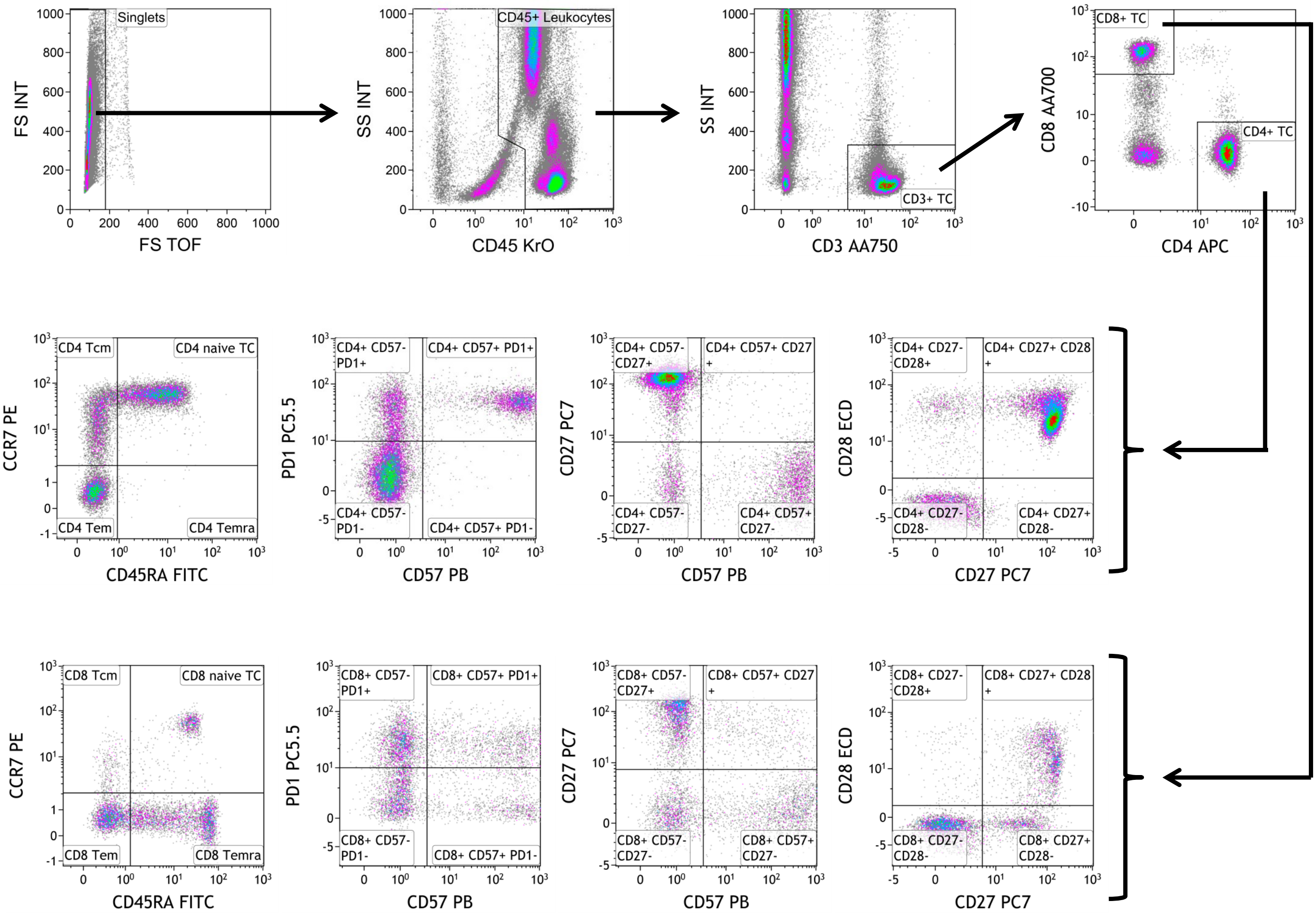

Supplementary Figure 1 *Manual gating strategy for DURAClone IM T Cell Subsets tube*

Flow cytometry data was analysed using the following gating strategy: after doublet exclusion, singlets were gated for CD45+ leukocytes. CD3+ T cell were gated within leukocyte population. CD4+ and CD8+ T cell subsets were gated within CD3+ cells. Memory T cells subsets were gated in CD4+ and CD8+ T cells population using CD45RA and CCR7. Furthermore, CD57/PD1, CD57/CD27 and CD27/CD28 subpopulations were determined in CD4+ and CD8+ T cell subsets.

Supplementary Figure 2

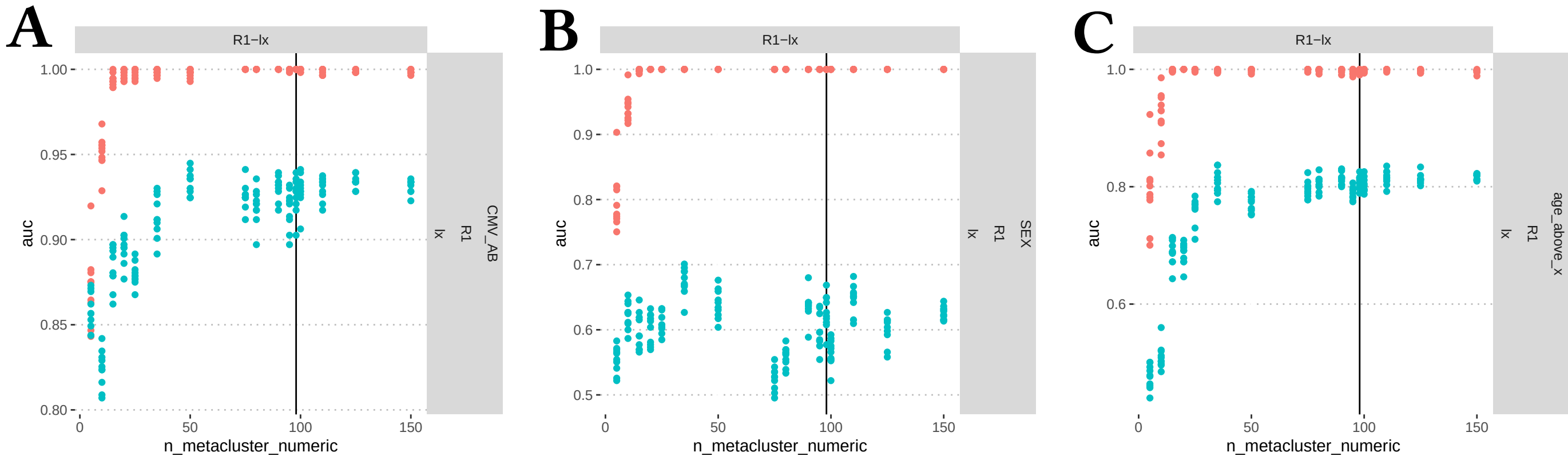

Supplementary Figure 2 *Predictive performance at different numbers of clusters*

Random forest models trained on R1 first presentation training samples on the CytoFLEX LX after relativisation. After training, performance on training (red) and validation (blue) sets of R1 first presentation samples was evaluated for varying numbers of clusters with ten random subsamples of 10,000 cells per sample. The y-axis denotes the area under the ROC curve. (A) Performance for CMV serostatus, (B) performances for sex and (C) performances for age group. The vertical line indicates the further used number of metaclusters for modelling (98).

Supplementary Figure 3

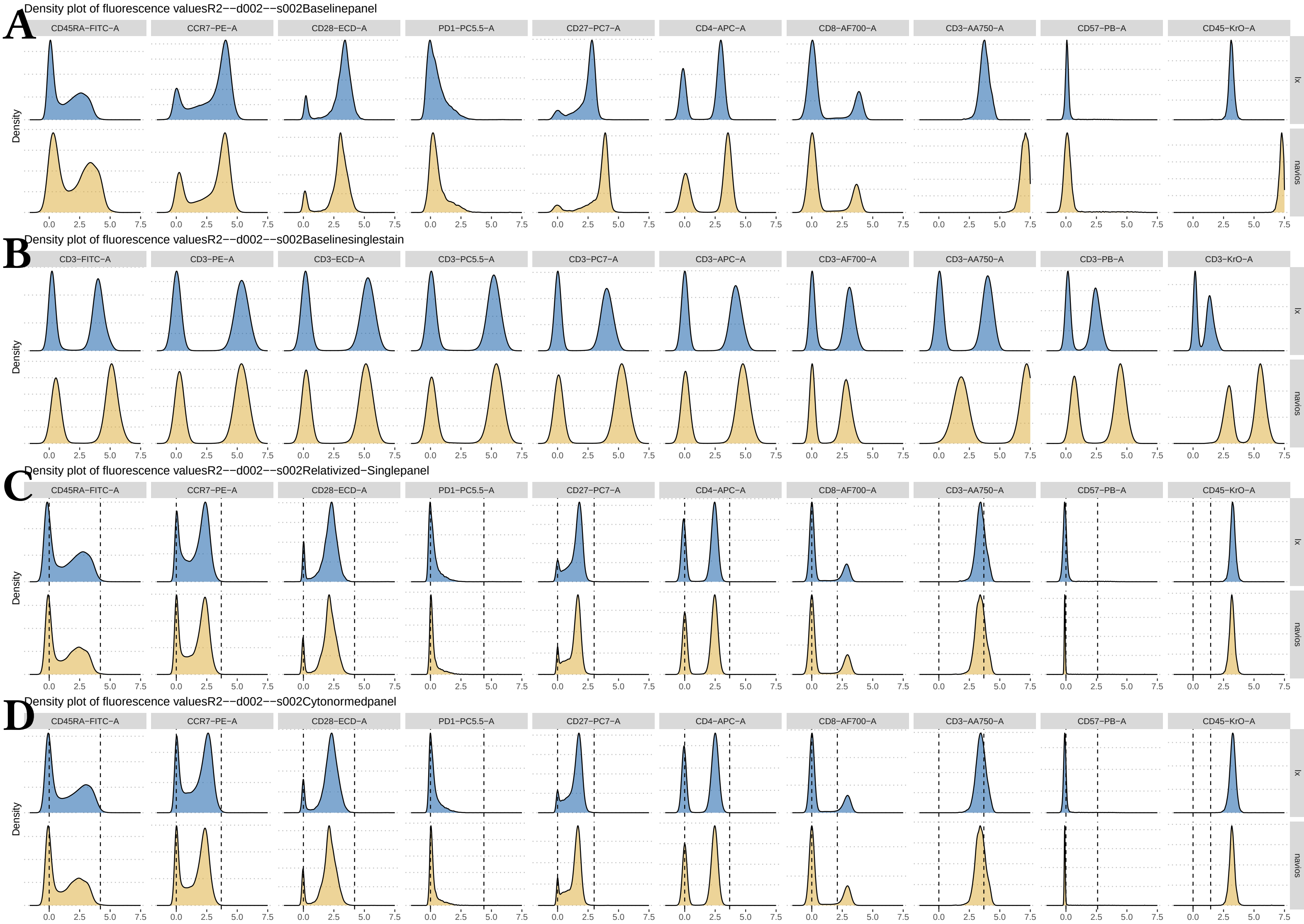

Supplementary Figure 3 *CD3 relativisation visualised for all channels*

Figure 4 illustrated CD3 relativisation using only the FITC-A channel for sample s002 from the R2 cohort. Here, we extend this visualization to include all channels for the same sample. In all plots, the x-axis shows arcsinh-transformed fluorescence intensity, the y-axis indicates density, and color distinguishes the measurement device. **(A)** Panel sample after arcsinh transformation. **(B)** CD3-Fluorochrome$_X$ single stained sample after arcsinh transformation. **(C)** Panel sample after CD3 relativisation, arcsinh transformed. Vertical lines represent 0 and $arcsinh(\frac{10000}{\text{cofactor}_{FCx}})$. **(D)** CD3-Fluorochrome$_X$ single stained sample after CD3 relativisation, arcsinh transformed. Vertical lines represent 0 and $arcsinh(\frac{10000}{\text{cofactor}_{FCx}})$. The positive populations of the single stained samples are naturally centered on the 'positive' vertical line after relativisation.

Supplementary Figure 4

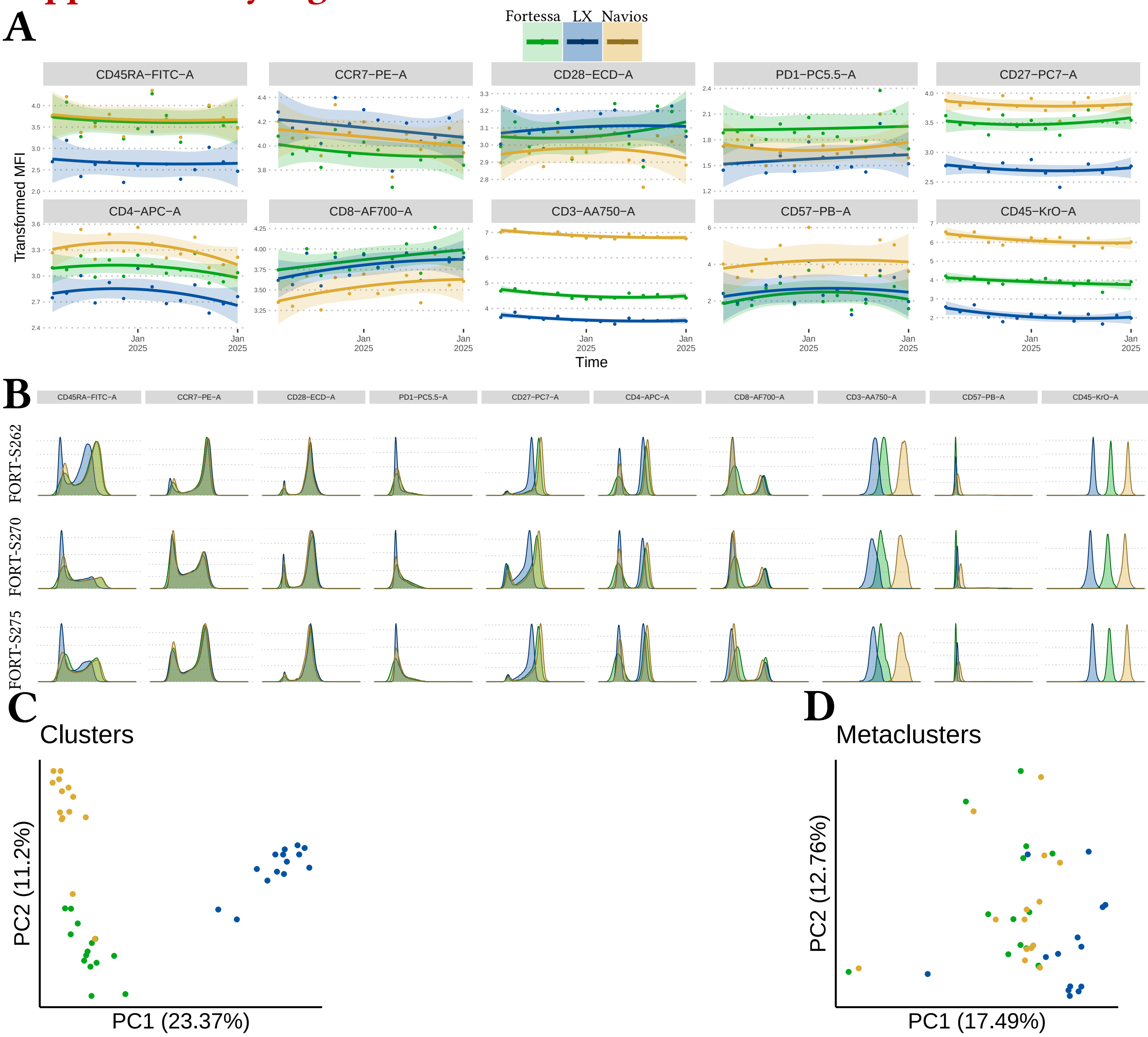

Supplementary Figure 4 *Unadjusted samples measured on Navios, CytoFLEX and BD LSRFortessa.*

Panels mirror those previously shown in Figure 2 for samples measured on three cytometers; Navios, CytoFLEX and BD LSRFortessa. **(A)** Longitudinal analysis of surface marker expression on manually gated positive immune cell subsets. Each subplot shows the arcsinh-transformed mean fluorescence intensity (MFI) of a specific marker over time. Points represent individual samples, colored by the cytometer used. Solid lines and shaded areas represent LOESS-smoothed trends and their confidence intervals. **(B)** Density plots of arcsinh-transformed marker intensities for three representative samples from the FORT cohort. Each subplot shows marker distributions for a single sample measured on different cytometers. **(C)** PCA of number of cells in each FlowSOM cluster on 14 samples measured on all three cytometers. Each point represents a sample colored by the measurement cytometer where clear clustering into cytometers is visible. **(D)** PCA of number of cells in each FlowSOM metacluster. Each point represents a sample colored by the measurement device where clear clustering is visible.

Supplementary Figure 5

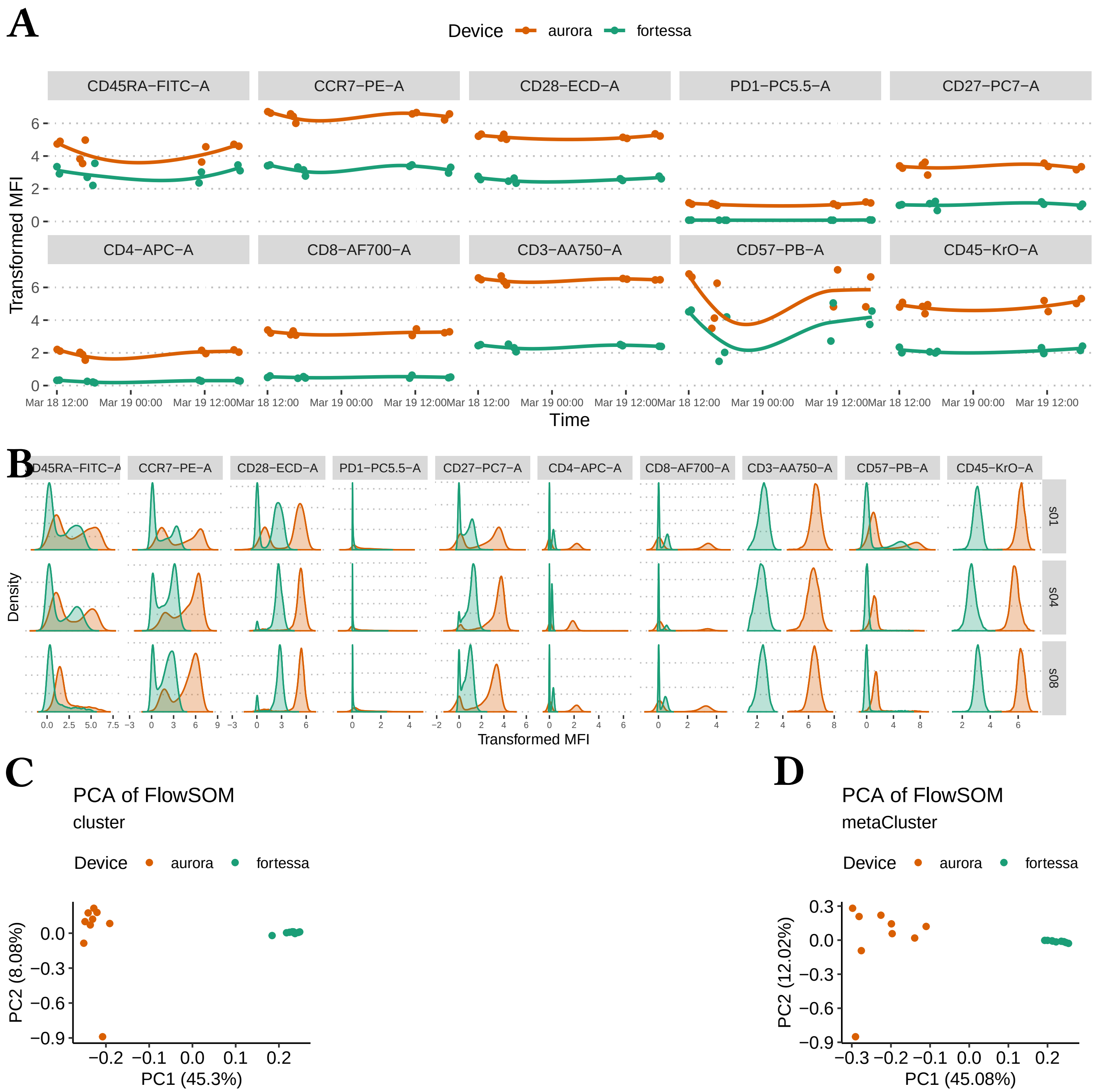

Supplementary Figure 5 *Within- and between-device variability comparing flow and spectral cytometry.*

Supplementary Figure 5 *Within- and between-device variability comparing flow and spectral cytometry.* Comparison of compensated values for the Badalona (BAD) cohort. Each sample was measured both on a classical flow cytometer (LSRFortessa) and a spectral cytometer (Cytek Aurora). The samples were then gated on lymphocytes. **(A)** Longitudinal cytometry analysis of surface marker expression on immune cell populations. Each subplot displays the mean fluorescence intensity of a specific marker over time, calculated on different manually gated cell subsets. The y-axis represents the fluorescence intensity after arcsinh transformation. Each point represents a single sample, colored by the device used for measurement. Fortessa is a classical cytometer, while Aurora is a spectral cytometer. **(B)** Density plots for three samples. Each subplot displays the density of the arcsinh transformed fluorescence intensity of a specific marker. The x-axis represents the fluorescence intensity, and the y-axis represents the density. Color represents the measurement device. **(C)** PCA of number of cells in each cluster of first presentations of all cohorts after FlowSOM built on all 9 samples. Each point represents a sample colored by the measurement device where clear clustering in the two devices is visible. **(D)** PCA of number of cells in each metacluster of first presentations of all cohorts after FlowSOM built on all 9 samples. Each point represents a sample colored by the measurement device where clear clustering in the two devices is visible.

Supplementary Figure 6

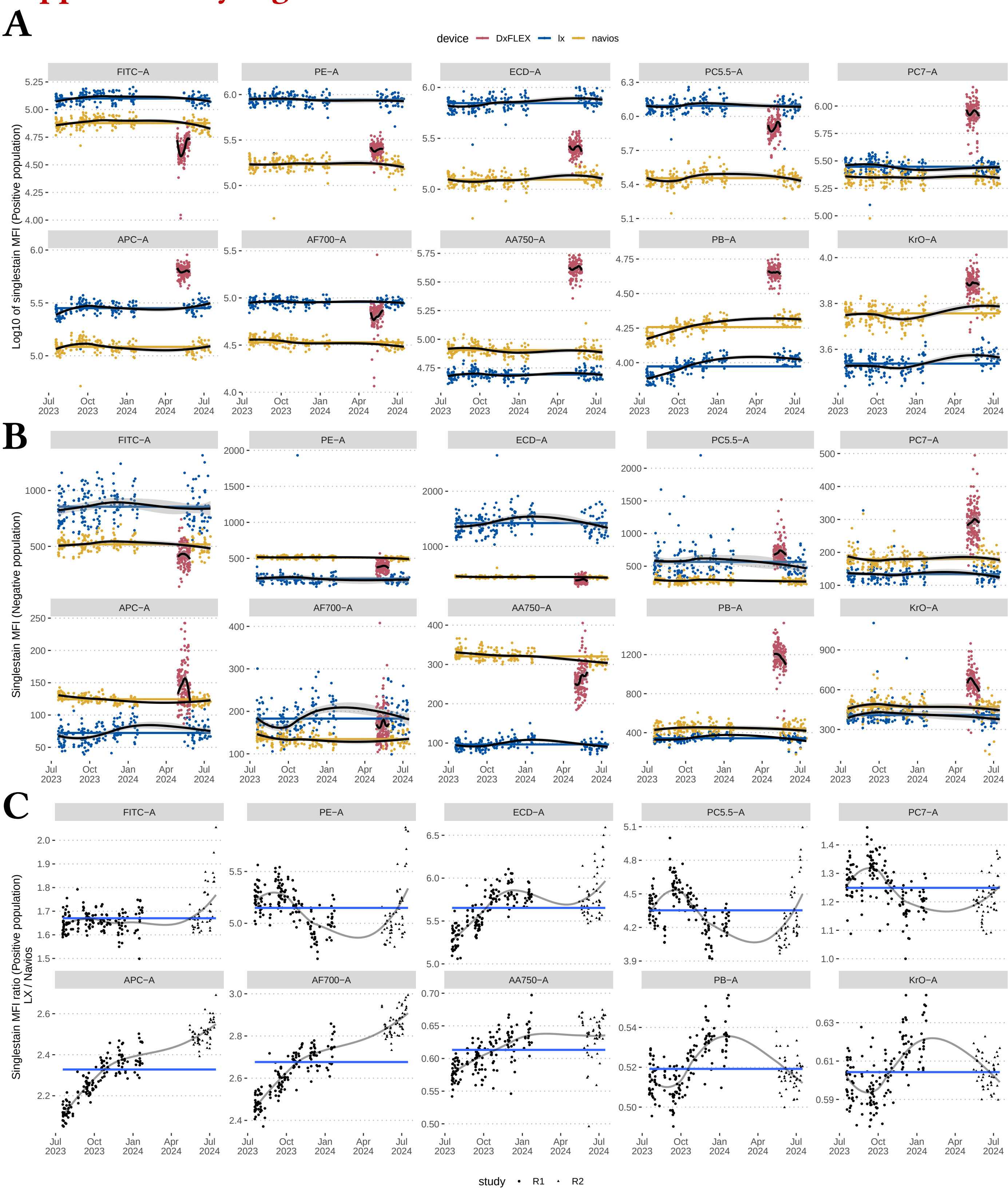

Supplementary Figure 6 *Median fluorescence intensities of single stained samples over time*

Supplementary Figure 6 *Median fluorescence intensities of single stained samples over time*

Longitudinal flow cytometry analysis of surface marker expression on single-stained samples from R1, R2 and SAN cohort. Compare to Figure 2A where the fluorescence intensity on the *panel sample* was shown. Each subplot shows the mean fluorescence intensity of CD3 stained with the respective fluorochrome of the positive **(A)** and negative **(B)** populations within gated lymphocytes. Each point represents a single sample, colored by the device used for measurement. Solid colored lines show the average fluorescence of the cytometer across time. Solid black lines and shaded areas are LOESS estimates and confidence intervals within cytometers. **(C)** Ratio of the median fluorescence intensity of the positive single stained population from (A) dividing LX by Navios per sample across markers. The point shape represents the study cohort R1 and R2. Solid gray line is the LOESS estimate, solid horizontal blue line is the overall mean. This shows that the LX and Navios cytometer drift differently over time.

Supplementary Figure 7

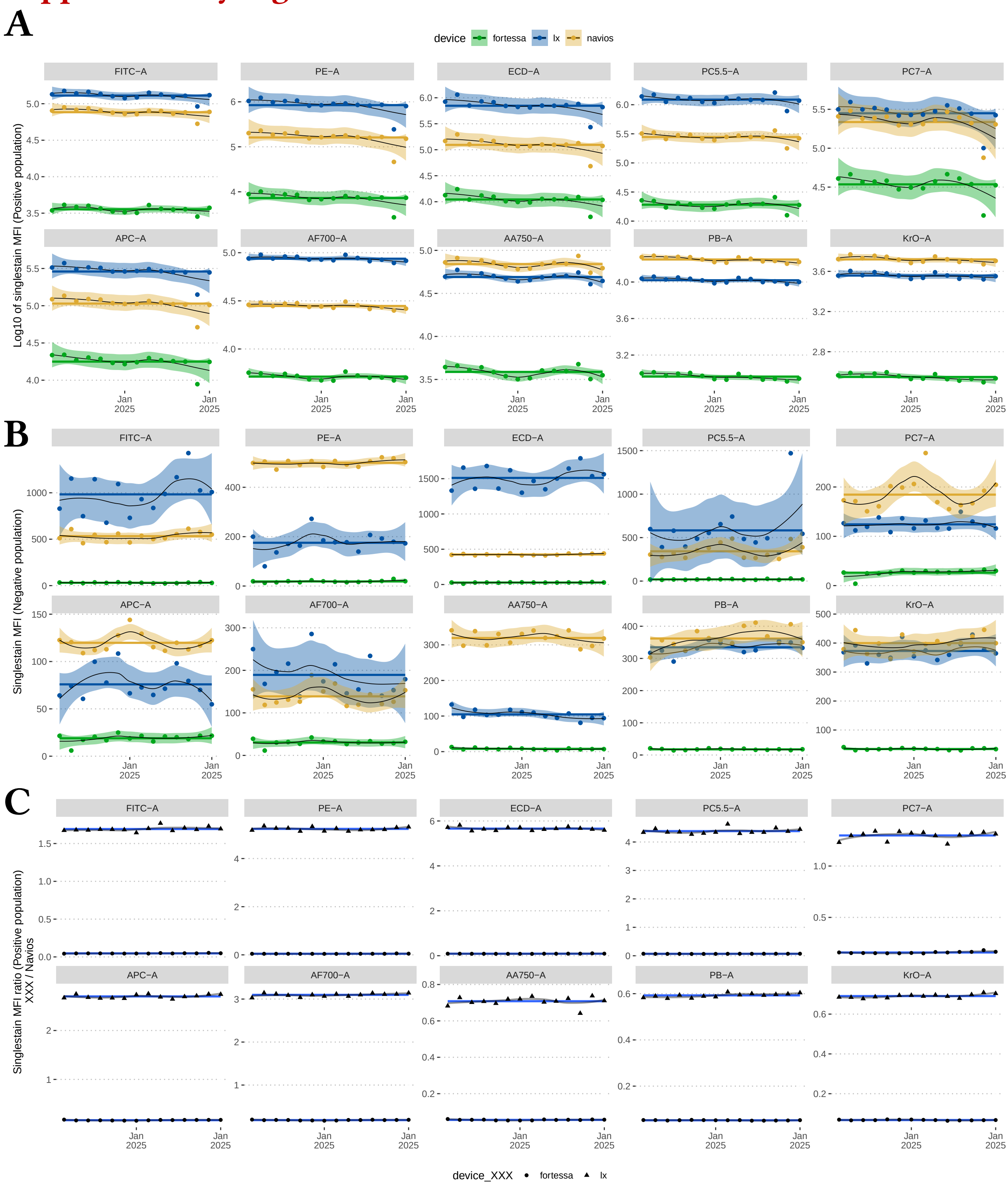

Supplementary Figure 7 *Median fluorescence intensities of single stained samples over time in the FORT cohort.*

Supplementary Figure 7 *Median fluorescence intensities of single stained samples over time in the FORT cohort.*

Longitudinal flow cytometry analysis of surface marker expression on single-stained samples from the FORT cohort where samples were measured on Navios, CytoFLEX and BD LSRFortessa. Each subplot shows the mean fluorescence intensity of CD3 stained with the respective fluorochrome of the positive **(A)** and negative **(B)** populations within gated lymphocytes. Each point represents a single sample, colored by the device used for measurement. Solid colored lines show the average fluorescence of the cytometer across time. Solid black lines and shaded areas are LOESS estimates and confidence intervals within cytometers. **(C)** shows the ratio of the median fluorescence intensity of the positive single stained population from (A) per sample across markers. The point shape represents the the ratio of (LX/Navios) and (Fortessa/Navios). Solid gray line is the LOESS estimate, solid horizontal blue line is the overall mean.

Supplementary Figure 8

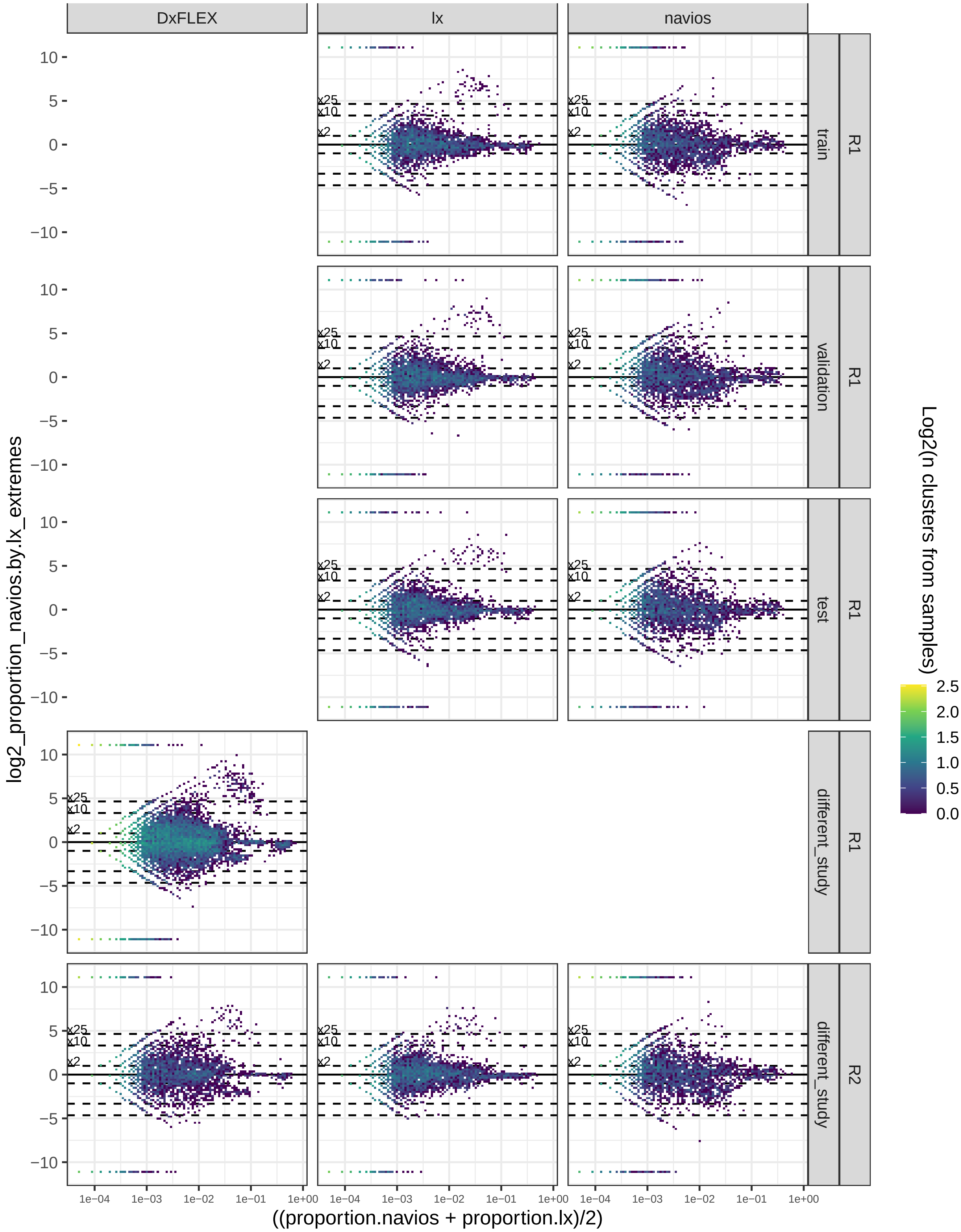

Supplementary Figure 8 *Bland-Altman plots for paired samples on Navios and LX across differently trained FlowSOMs*

Supplementary Figure 8 *Bland-Altman plots for paired samples on Navios and LX across differently trained FlowSOMs*

Bland-Altman plots of metacluster cell proportions reveal over 25-fold differences between Navios and LX across all first-presentation R1 and R2 samples measured on both cytometers. Each column represents one of three FlowSOM models, each trained on 400 clusters grouped into 98 metaclusters using training samples from SAN-DxFLEX, R1-LX, or R1-Navios. Clusterings were then applied to all samples measured on either the Navios and LX. Specifically, the SAN-DxFLEX clustering was applied to R2 and all R1 first-presentation samples. Dashed lines indicate 2-, 10-, and 25-fold differences, while color intensity reflects the frequency of sample-metacluster combinations showing each fold change. For instance, the top-right plot shows a model trained on R1-Navios and applied to R1 training samples. Plot layout and axis limits are consistent across all panels and match those in Supplementary Figure 13. Points at the y-axis extremes correspond to metaclusters with zero cells in one of the paired samples and were manually positioned for visualization. These findings underscore the limited cross-device comparability of metacluster quantification.

Supplementary Figure 9

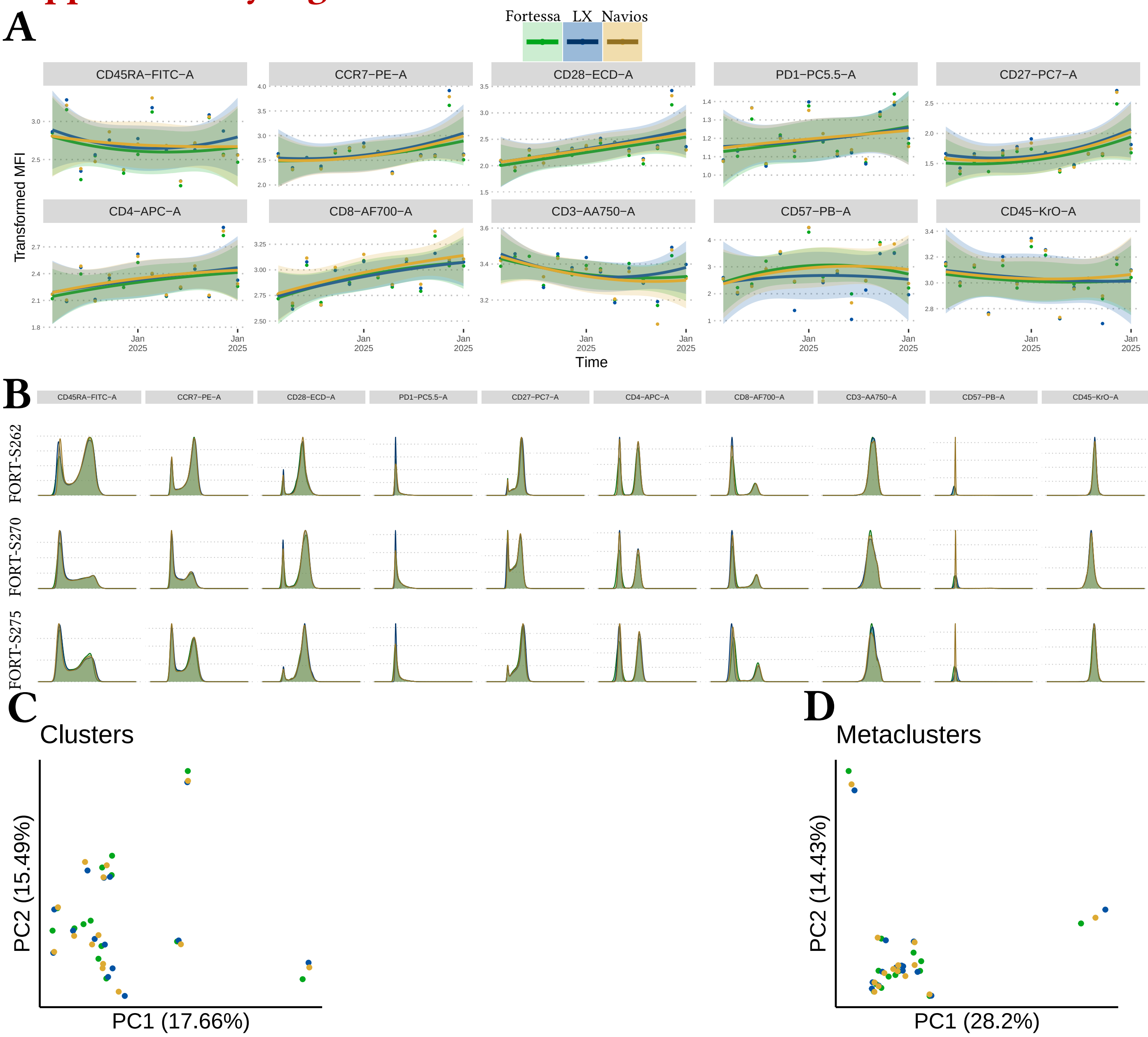

Supplementary Figure 9 *CD3 relativised data from samples measured on Navios, CytoFLEX and BD LSRFortessa.*

Supplementary Figure 9 *CD3 relativised data from samples measured on Navios, CytoFLEX and BD LSRFortessa.*

Panels mirror those shown in Supplementary Figure 4, with samples measured on Navios, LX and LSR. Each sample has been CD3-relativised as described in Figure 4. Each fluorochrome was corrected independently, based on its own single-stained CD3+ control, allowing cytometer-independent comparison without the need for matched or external reference samples. **(A)** Longitudinal analysis of surface marker expression on manually gated positive immune cell subsets. Each subplot shows the arcsinh-transformed mean fluorescence intensity (MFI) of a specific marker over time. Points represent individual samples, colored by the cytometer used. Solid lines and shaded areas represent LOESS-smoothed trends and their confidence intervals. **(B)** Density plots of arcsinh-transformed marker intensities for three representative samples from the FORT cohort. Each subplot shows marker distributions for a single sample measured on different cytometers. Relativisation reduces marker-specific intensity shifts. **(C)** PCA of number of cells in each FlowSOM cluster on 14 samples measured on all three cytometers. Each point represents a sample colored by the measurement cytometer where no clustering into cytometers remained. **(D)** PCA of number of cells in each FlowSOM metacluster. Each point represents a sample colored by the measurement device where no clustering into cytometers remained.

Supplementary Figure 10

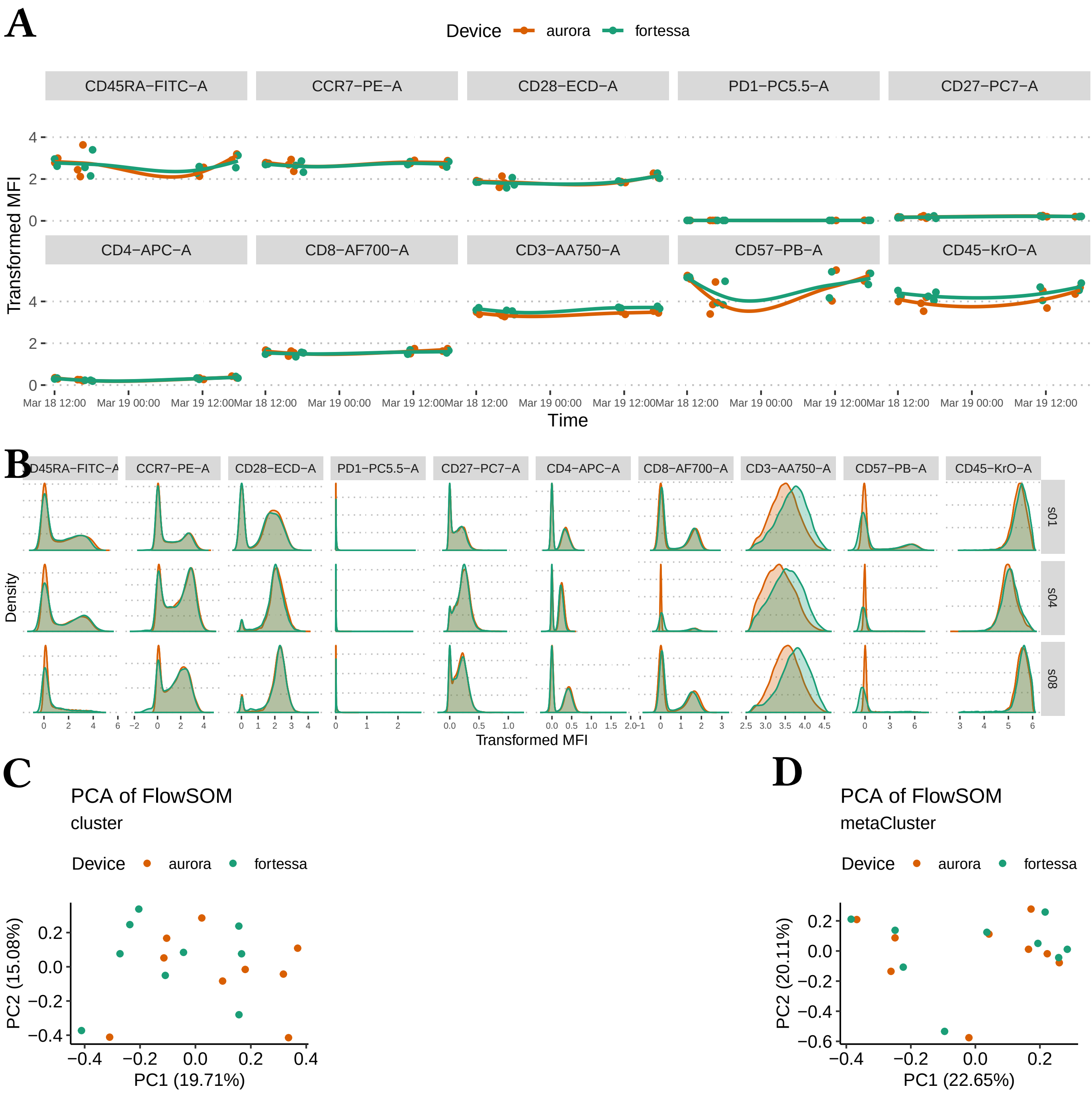

Supplementary Figure 10 *Within- and between- device variability comparing flow and spectral cytometry after CD3 relativisation*

Supplementary Figure 10 *Within- and between- device variability comparing flow and spectral cytometry after CD3 relativisation*

Comparison of compensated and relativised values for the Badalona (BAD) cohort. Each sample was measured both on a classical flow cytometer (LSRFortessa) and a spectral cytometer (Cytek Aurora). The samples were then gated on lymphocytes. **(A)** Longitudinal cytometry analysis of surface marker expression on immune cell populations. Each subplot displays the mean fluorescence intensity of a specific marker over time, calculated on different manually gated cell subsets. The y-axis represents the fluorescence intensity after arcsinh transformation. Each point represents a single sample, colored by the device used for measurement. Fortessa is a classical cytometer, while Aurora is a spectral cytometer. **(B)** Density plots for three samples. Each subplot displays the density of the arcsinh transformed fluorescence intensity of a specific marker. The x-axis represents the fluorescence intensity, and the y-axis represents the density. Color represents the measurement device. **(C)** PCA of number of cells in each cluster of first presentations of all cohorts after FlowSOM built on all 9 samples. Each point represents a sample colored by the measurement device where small batch effects but no clustering in devices is visible. **(D)** PCA of number of cells in each metacluster of first presentations of all cohorts after FlowSOM built on all 9 samples. Each point represents a sample colored by the measurement device where no batch effects are visible. **Comparing to Supplementary Figure 5, relativisation enables direct numerical comparability between cytometers.**

Supplementary Figure 11

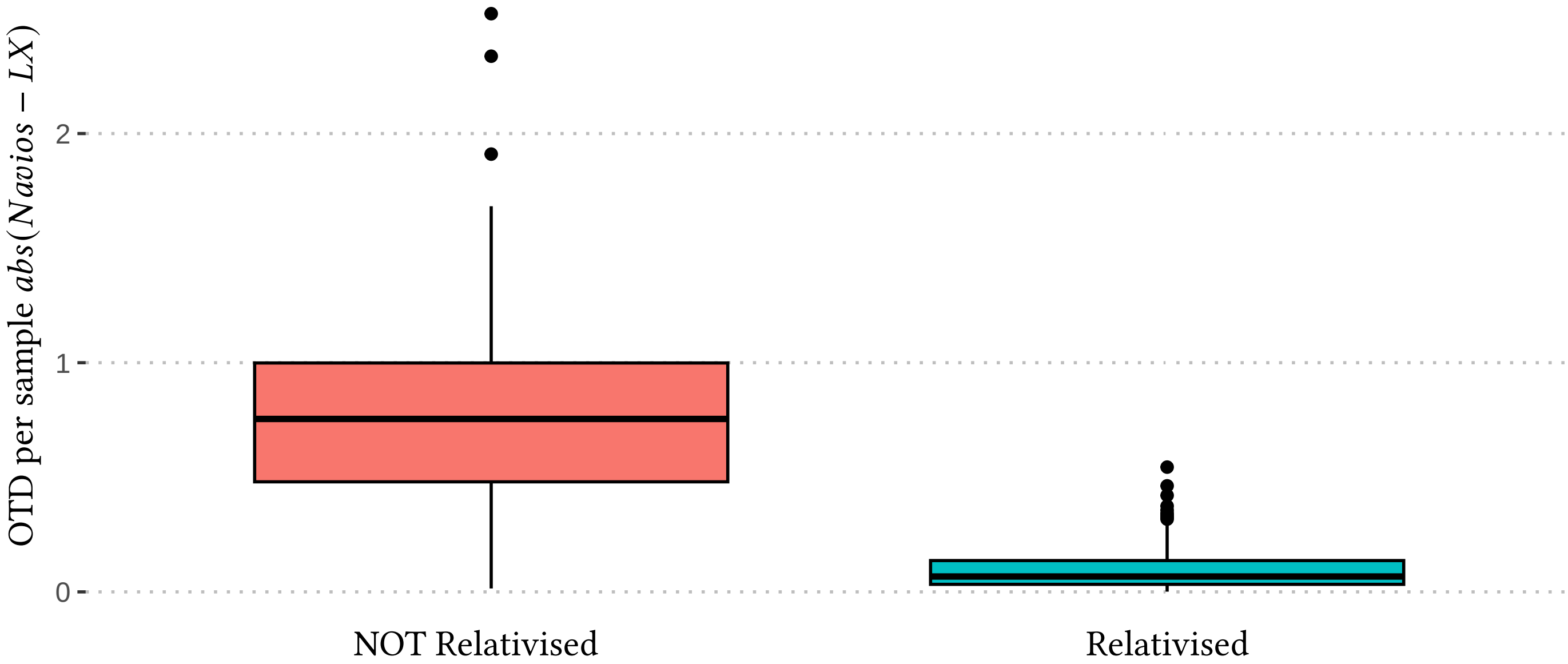

Supplementary Figure 11 *Quantification of instrument agreement using paired optimal transport distances.*

Optimal transport distances (OTD) quantify the dissimilarity between a sample and an aggregate reference in the full multi-marker space (see Methods). Here, we leveraged the paired design of the R1 and R2 cohorts, where each sample was measured on both Navios and LX cytometers. For each sample i, we computed the absolute OTD difference between instruments, $|\Delta_i| = |\text{OTD}(\text{sample}_i, \text{Navios}) - \text{OTD}(\text{sample}_i, \text{LX})|$, as a direct measure of instrument disagreement. CD3-relativisation reduced the median $|\Delta|$ approximately 10-fold, from 0.755 to 0.067 (Wilcoxon signed-rank test, $p < 0.001$; SD: 0.390 to 0.087; IQR: 0.519 to 0.104).

Supplementary Figure 12

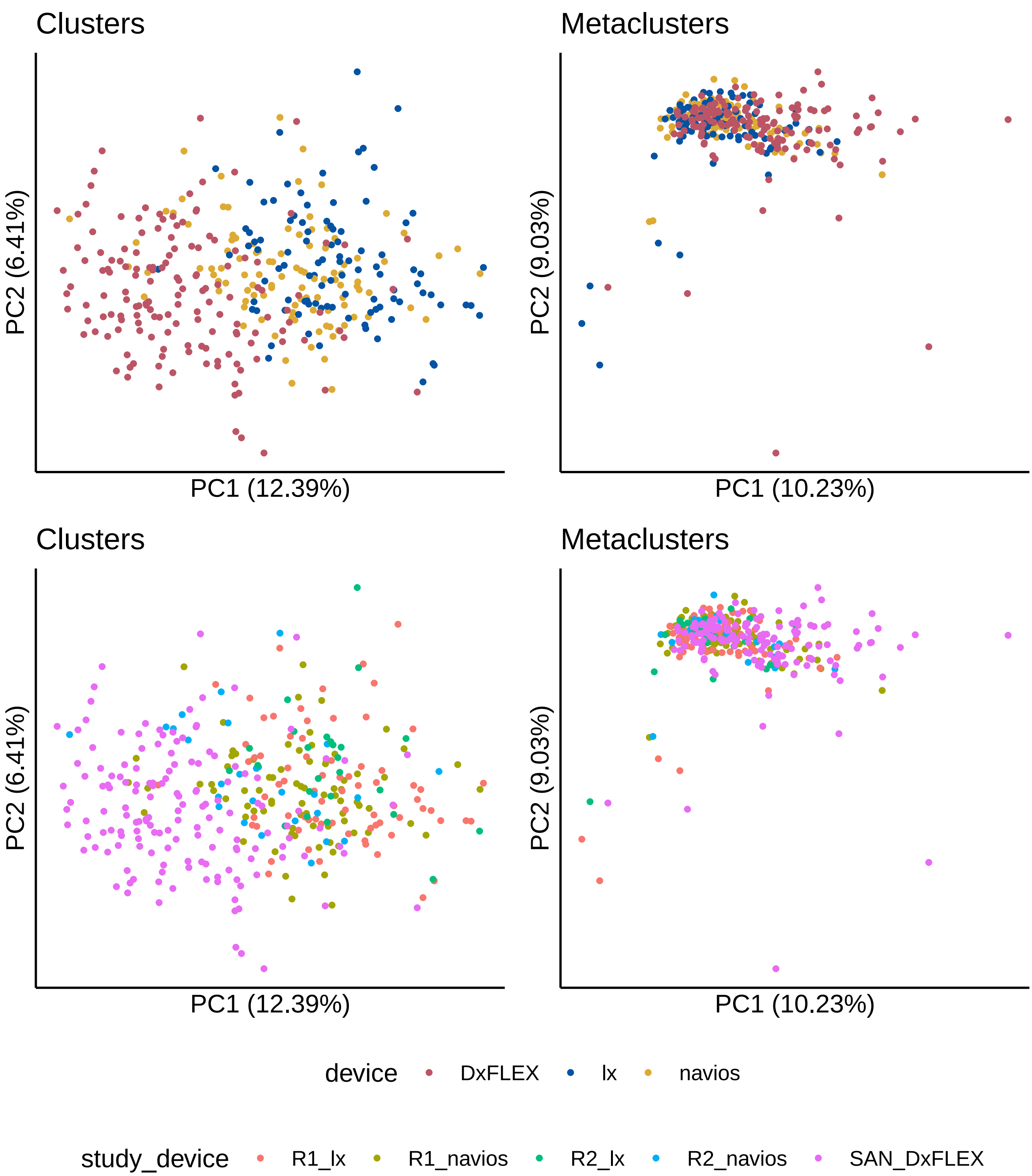

Supplementary Figure 12 *PCA plots of cluster and metacluster proportions after relativisation*

Supplementary Figure 12 *PCA plots of cluster and metacluster proportions after relativisation*
Principal component analysis (PCA) of cell proportions per cluster (left) and metacluster (right) following relativisation. Plots show the first two principal components, with samples colored by cytometer (top panels) or by both cytometer and study (bottom panels). Cytometer-specific clustering patterns are no longer apparent, indicating effective alignment. Samples from R1 and R2 are not visibly separated, suggesting successful alignment of immune cell population distributions across time points.

Supplementary Figure 13

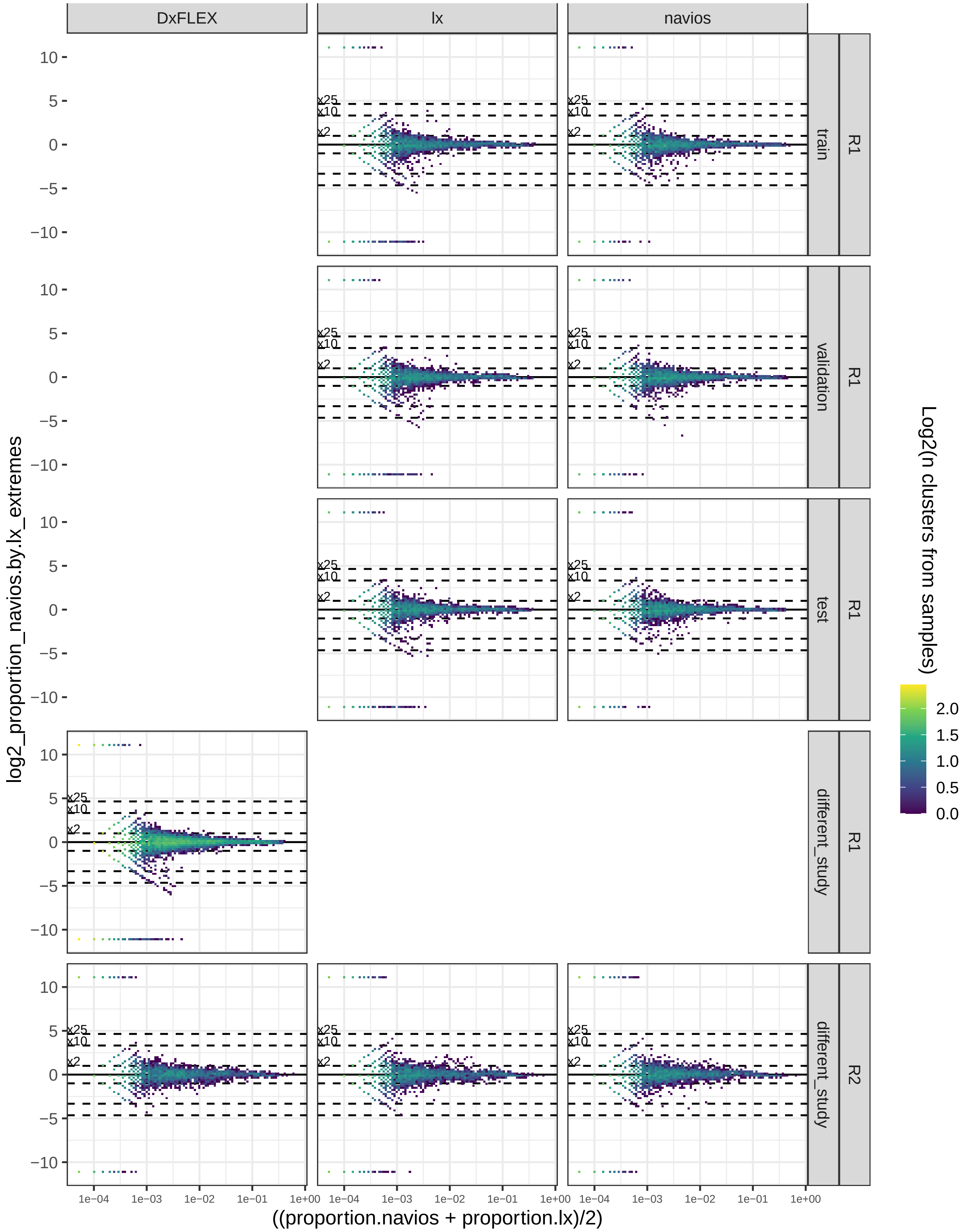

Supplementary Figure 13 *Bland-Altman plots for paired Navios and LX samples across FlowSOM models after relativisation*

Supplementary Figure 13 *Bland-Altman plots for paired Navios and LX samples across FlowSOM models after relativisation*

After relativisation, Bland-Altman plots of metacluster cell proportions reveal considerably reduced differences between Navios and LX across all first-presentation R1 and R2 samples measured on both cytometers. As in Supplementary Figure 8, each column represents one of three FlowSOM models, each trained on 400 clusters grouped into 98 metaclusters using training samples from SAN-DxFLEX, R1-LX, or R1-Navios. Clusterings were then applied to all samples measured on either the Navios and LX after relativisation. Specifically, the SAN-DxFLEX clustering was applied to R2 and all R1 first-presentation samples. Dashed lines indicate 2-, 10-, and 25-fold differences, while color intensity reflects the frequency of sample-metacluster combinations showing each fold change. For instance, the top-right plot shows a model trained on R1-Navios and applied to R1 training samples. Plot layout and axis limits are consistent across all panels and match those in Supplementary Figure 8. Points at the y-axis extremes correspond to metaclusters with zero cells in one of the paired samples and were manually positioned for visualization. **These results demonstrate that relativisation substantially improves inter-device agreement in metacluster quantification, leading to more consistent and comparable clustering across cytometers.**

Supplementary Figure 14

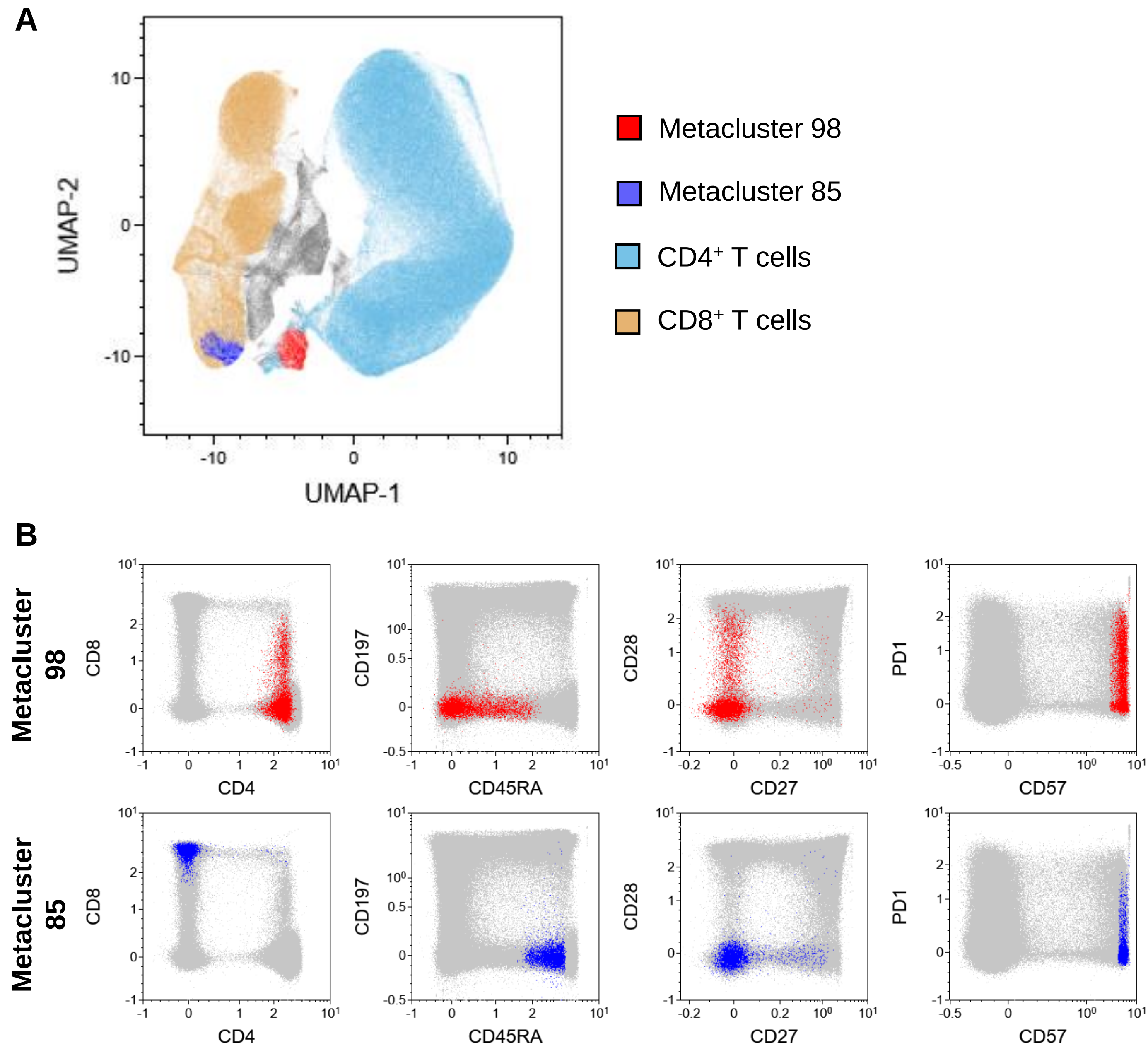

Supplementary Figure 14 *Phenotypic characterization of important CMV serostatus metaclusters 98 and 85*

(A) UMAP projection of the T cell compartment, with cells colored by population: metacluster 98 (red), metacluster 85 (blue), $CD4^+$ T cells (light blue), and $CD8^+$ T cells (orange).(B) Biaxial dot plots showing expression of markers for metacluster 98 (top row, red) and metacluster 85 (bottom row, blue), overlaid on all T cells (grey). Both metaclusters reveal an expansion of $CD57^{high}$ effector T cells.

Supplementary Figure 15

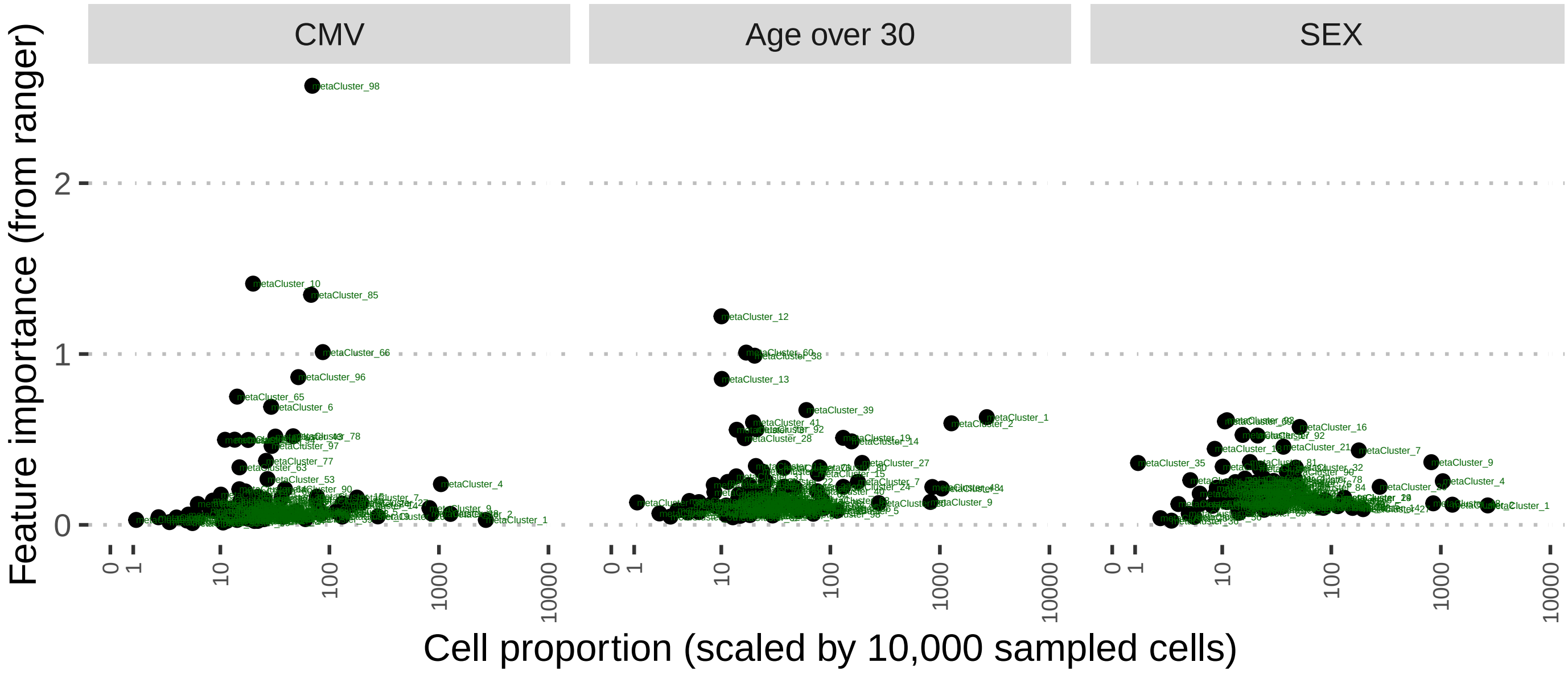

Supplementary Figure 15 *Feature importance vs. average cluster size*

Feature importance values from ranger (impurity) plotted against the mean abundance of all 98 metaclusters across all samples in R1 measured on the Navios after relativisation. Each point represents one cluster. The x-axis is on a pseudo-log10 scale also showing 0. For CMV serostatus, metaclusters 98, 10 and 85 were most important, for age over 30 metaclusters 12, 60 and 38. For sex, while anyways weak overall, metaclusters 69, 12 and 16 were most important.

Supplementary Figure 16

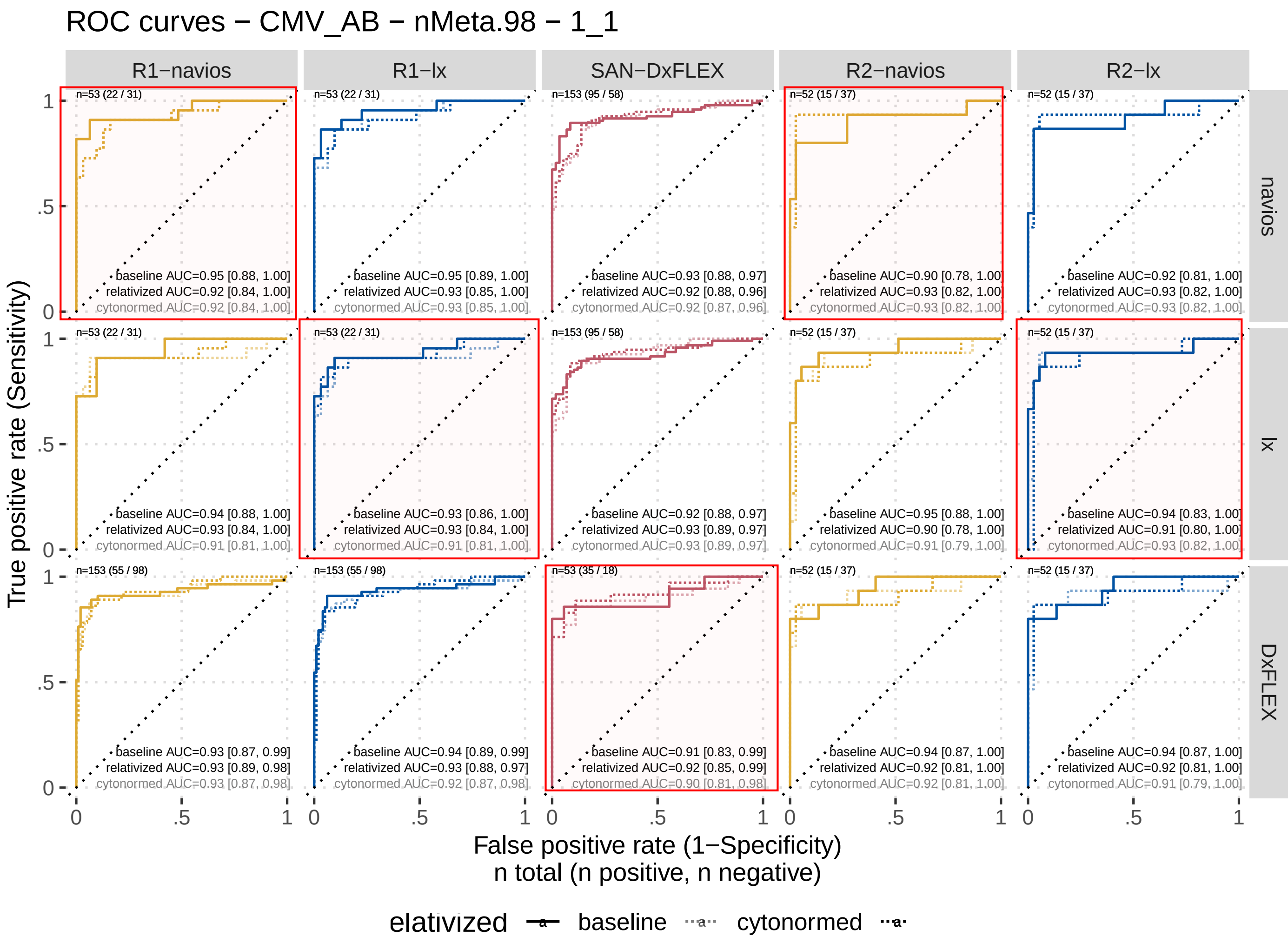

Supplementary Figure 16 *ROC curves for CMV-IgG seropositivity classification across devices after relativisation*

Receiver operating characteristic (ROC) curves for CMV-IgG$^+$ classification on first-presentation samples from each donor, comparing model performance across three pre-processing conditions: no normalisation, relativisation alone, and relativisation combined with CytoNorm. Solid lines indicate models trained and applied to non-relativised data; dotted lines show performance on relativised; dotted, shaded lines show performance on relativised then cytonormed data. Rows correspond to models trained on samples from the Navios, CytoFLEX LX, or DxFLEX cytometers. Columns indicate the applied test or prospective set, labeled by cohort and cytometer (e.g., R1-Navios). Random forest models were trained on R1 or SAN training samples per cytometer. For models trained on Navios or CytoFLEX LX data, we tested on R1 test samples, all of R2, and all of SAN. For models trained on DxFLEX data, we tested on SAN test samples, all of R1, and all of R2. The x-axis denotes the false positive rate; the y-axis denotes the true positive rate. The diagonal line represents random performance. Area under the curve (AUC) values with DeLong confidence intervals are shown in the lower-right corner of each plot. The ROC curves marked with red boxes have been shown in Figure 3A.

Supplementary Figure 17

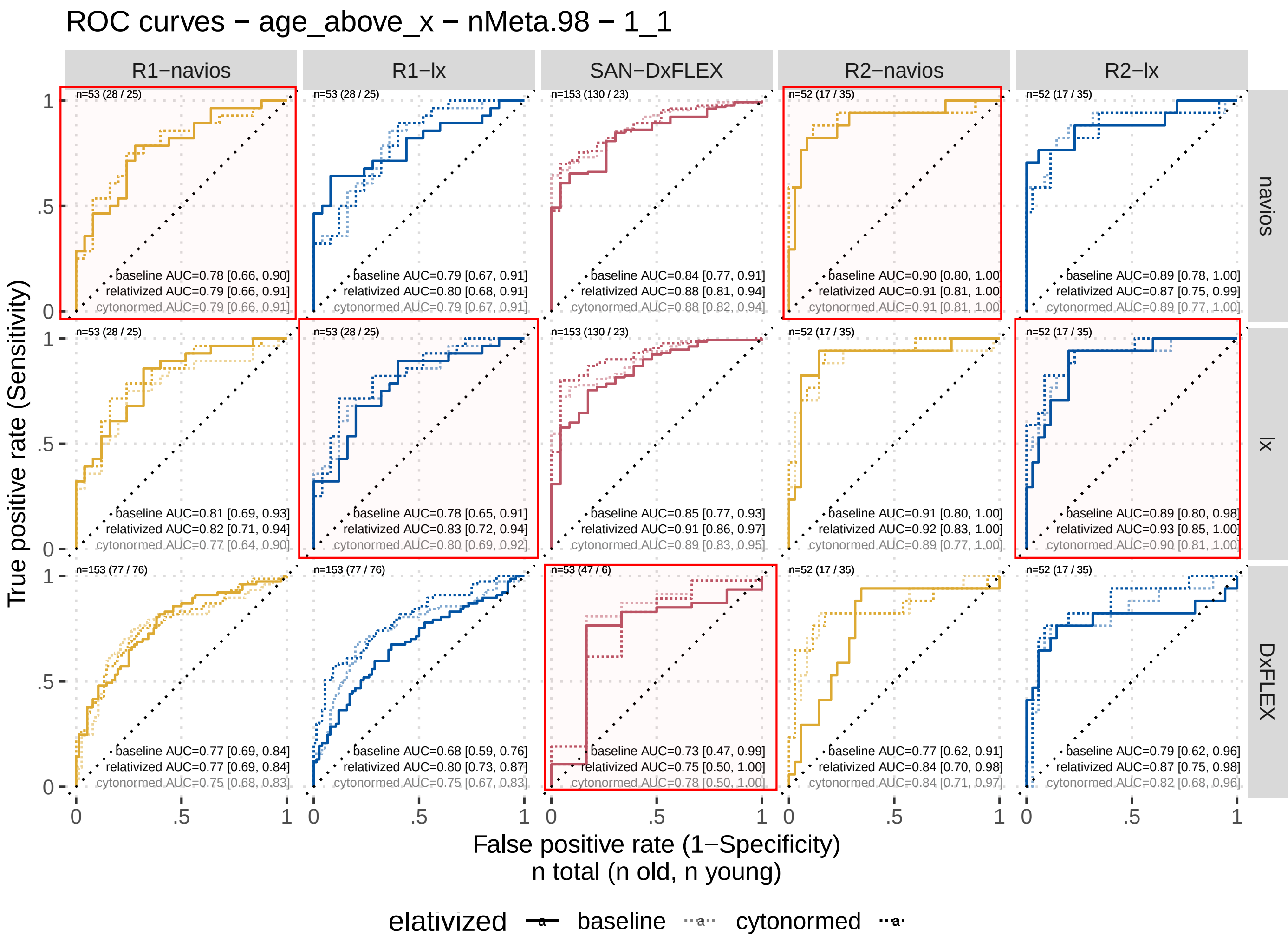

Supplementary Figure 17 *ROC curves for age across different devices post relativisation*

Receiver operating characteristic (ROC) curves for classification of age above 30 on first-presentation samples from each donor, comparing model performance across three preprocessing conditions: no normalisation, relativisation alone, and relativisation combined with CytoNorm. Solid lines indicate models trained and applied to non-relativised data; dotted lines show performance on relativised; dotted, shaded lines show performance on relativised then cytonormed data. Rows correspond to models trained on samples from the Navios, CytoFLEX LX, or DxFLEX cytometers. Columns indicate the applied test or prospective set, labeled by cohort and cytometer (e.g., R1-Navios). Random forest models were trained on R1 or SAN training samples per cytometer. For models trained on Navios or CytoFLEX LX data, we tested on R1 test samples, all of R2, and all of SAN. For models trained on DxFLEX data, we tested on SAN test samples, all of R1, and all of R2. The x-axis denotes the false positive rate; the y-axis denotes the true positive rate. The diagonal line represents random performance. Area under the curve (AUC) values with DeLong confidence intervals are shown in the lower-right corner of each plot. The ROC curves marked with red boxes have been shown in Figure 3A.

Supplementary Figure 18

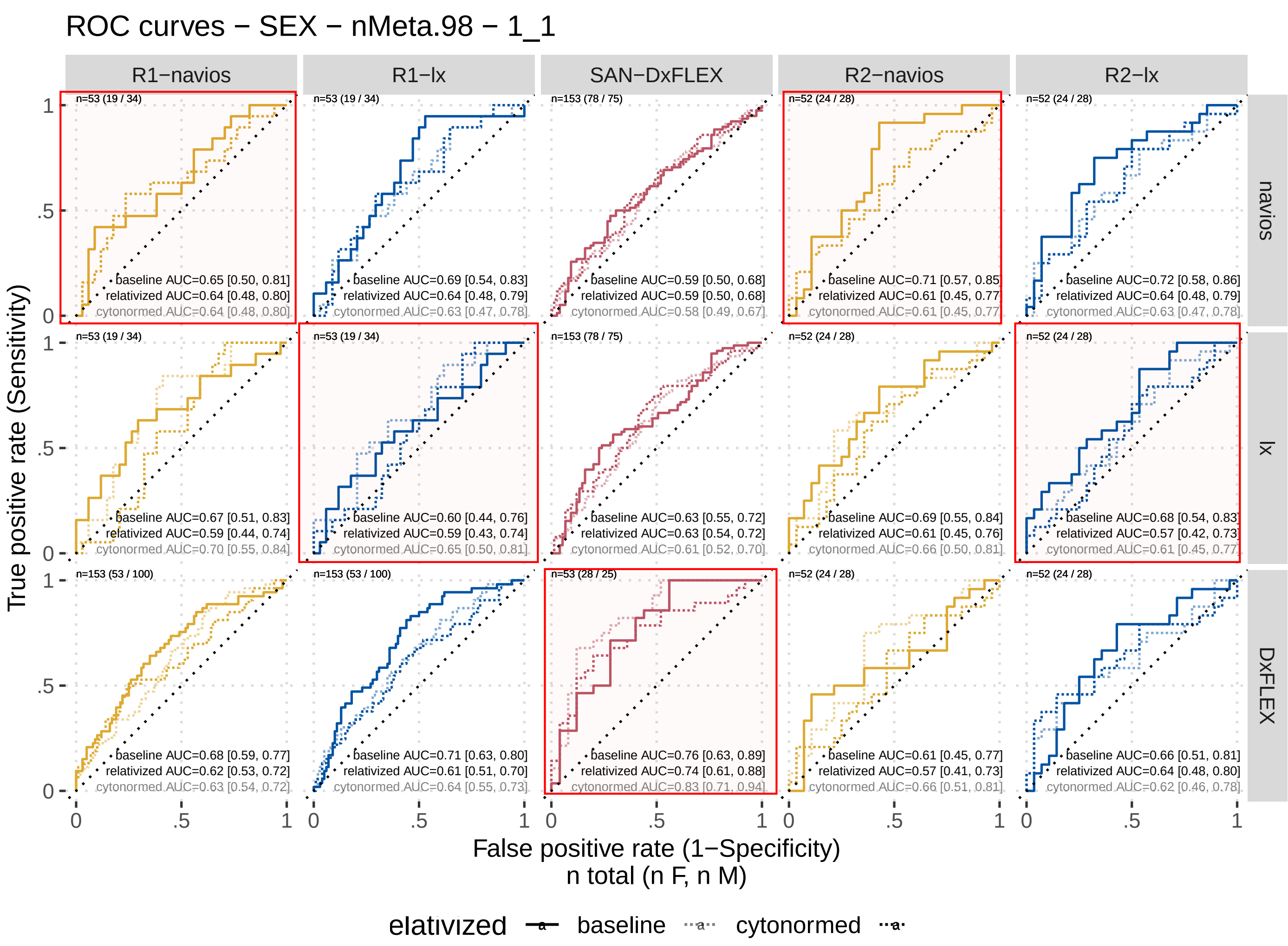

Supplementary Figure 18 *ROC curves for sex across different devices post relativisation*

Supplementary Figure 18 *ROC curves for sex across different devices post relativisation*

Receiver operating characteristic (ROC) curves for classification of sex on first-presentation samples from each donor, comparing model performance across three preprocessing conditions: no normalisation, relativisation alone, and relativisation combined with CytoNorm. Solid lines indicate models trained and applied to non-relativised data; dotted lines show performance on relativised; dotted, shaded lines show performance on relativised then cytonormed data. Rows correspond to models trained on samples from the Navios, CytoFLEX LX, or DxFLEX cytometers. Columns indicate the applied test or prospective set, labeled by cohort and cytometer (e.g., R1-Navios). Random forest models were trained on R1 or SAN training samples per cytometer. For models trained on Navios or CytoFLEX LX data, we tested on R1 test samples, all of R2, and all of SAN. For models trained on DxFLEX data, we tested on SAN test samples, all of R1, and all of R2. The x-axis denotes the false positive rate; the y-axis denotes the true positive rate. The diagonal line represents random performance. Area under the curve (AUC) values with DeLong confidence intervals are shown in the lower-right corner of each plot. The ROC curves marked with red boxes have been shown in Figure 3A.

Supplementary Figure 19

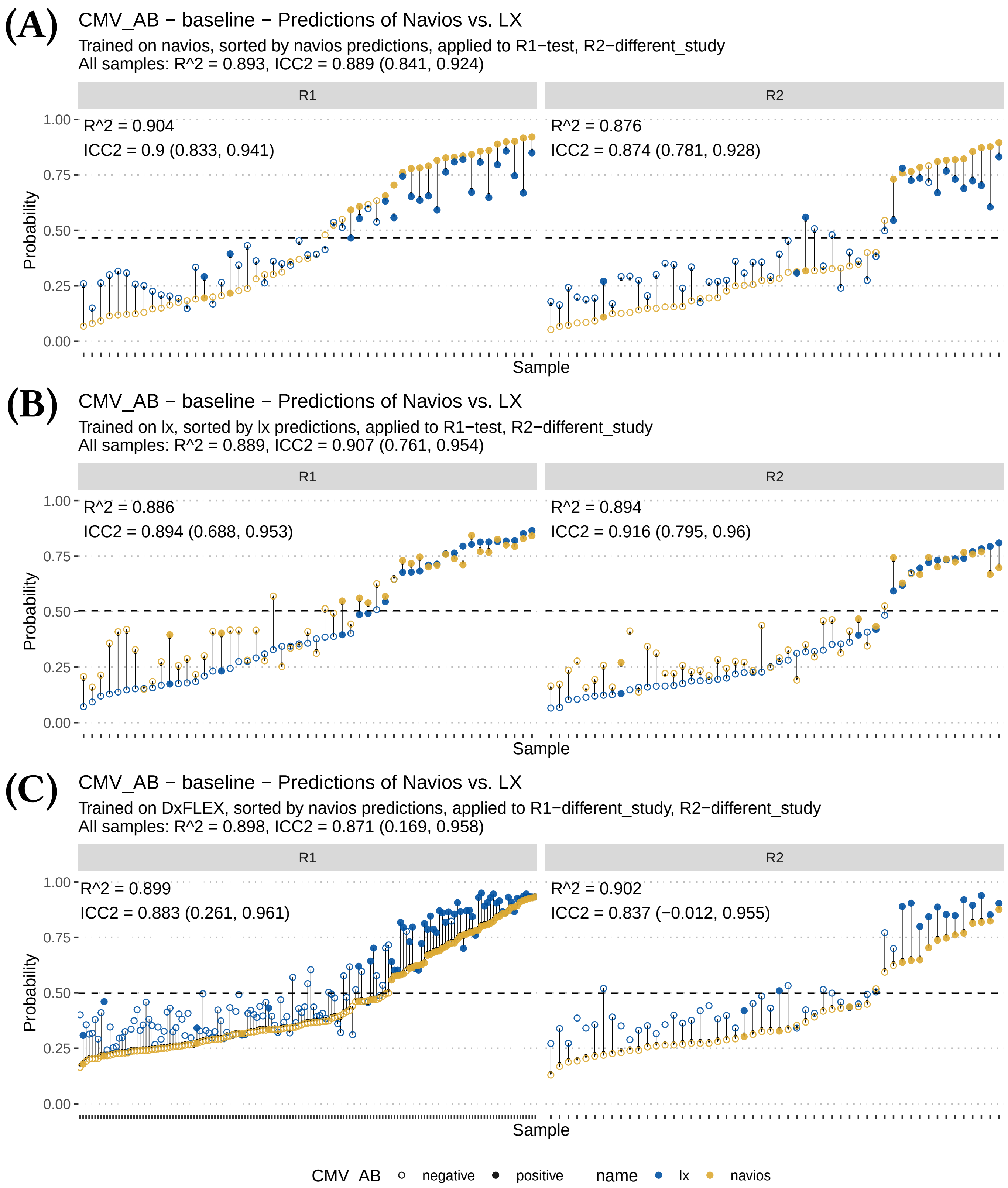

Supplementary Figure 19 *Predicted probabilities of CMV seropositivity across cytometers*

Supplementary Figure 19 *Predicted probabilities of CMV seropositivity across cytometers*

Predicted probabilities of CMV-IgG^+ based on the proportions of cells in each of 98 FlowSOM metaclusters. Models were trained on samples measured with the Navios **(A)**, LX **(B)**, or DxFLEX **(C)** cytometer and applied to samples measured on both Navios and LX. Each point represents one sample; color indicates the cytometer. Filled circles correspond to CMV-IgG^+ samples, open circles to CMV-IgG^- samples. Samples are ordered by predicted probability from the training-cytometer data predictions. The dashed line indicates the classification threshold, defined as the point on the ROC curve closest to the top-left corner using validation data.

Supplementary Figure 20

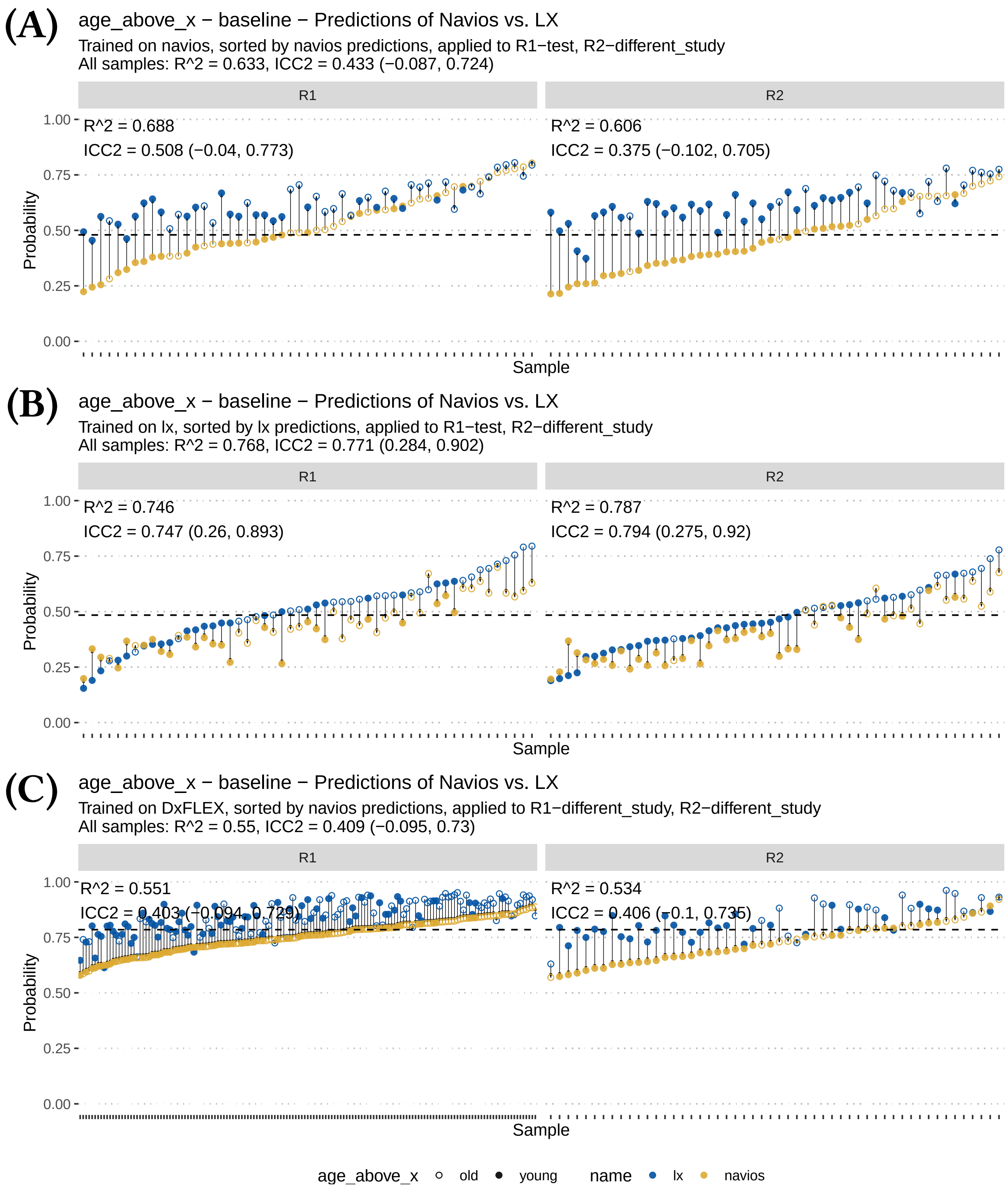

Supplementary Figure 20 *Predicted probabilities of age above 30 based on metacluster cell proportions*

Supplementary Figure 20 *Predicted probabilities of age above 30 based on metacluster cell proportions* Predicted probabilities of age above 30 based on the proportions of cells in each of 98 FlowSOM metaclusters. Models were trained on samples measured with the Navios **(A)**, LX **(B)**, or DxFLEX **(C)** cytometer and applied to samples measured on both Navios and LX. Each point represents one sample; color indicates the cytometer. Filled circles correspond to age up to 30 samples, open circles to above 30 samples. Samples are ordered by predicted probability from the training-cytometer data predictions. The dashed line indicates the classification threshold, defined as the point on the ROC curve closest to the top-left corner using validation data.

Supplementary Figure 21

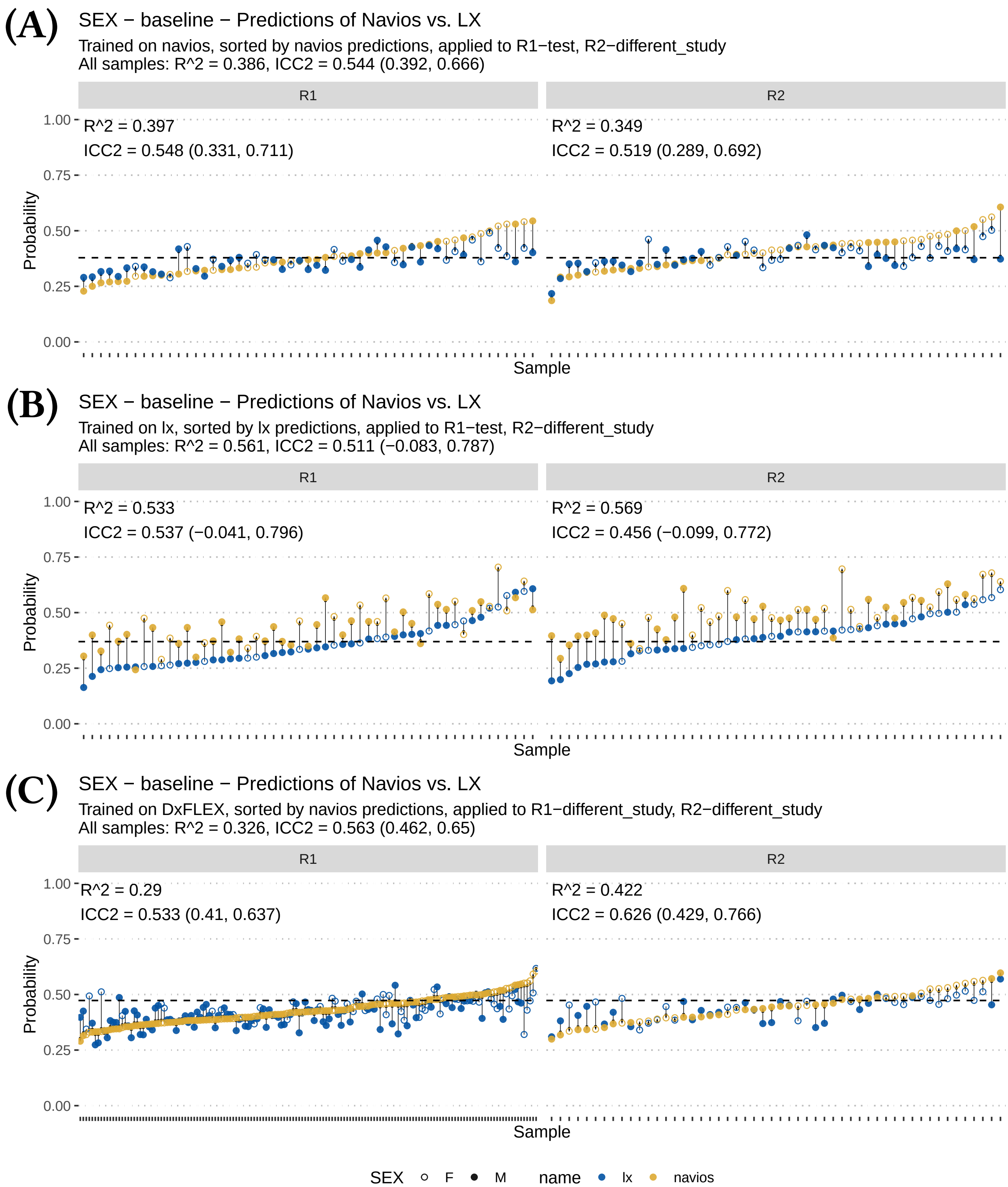

Supplementary Figure 21 *Predicted probabilities of sex based on metacluster cell proportions*

Supplementary Figure 21 *Predicted probabilities of sex based on metacluster cell proportions*

Predicted probabilities of sex based on the proportions of cells in each of 98 FlowSOM metaclusters. Models were trained on samples measured with the Navios **(A)**, LX **(B)**, or DxFLEX **(C)** cytometer and applied to samples measured on both Navios and LX. Each point represents one sample; color indicates the cytometer. Filled circles denote male samples; open circles denote female samples. Samples are ordered by predicted probability from the training-cytometer data predictions. The dashed line indicates the classification threshold, defined as the point on the ROC curve closest to the top-left corner using validation data.

Supplementary Figure 22

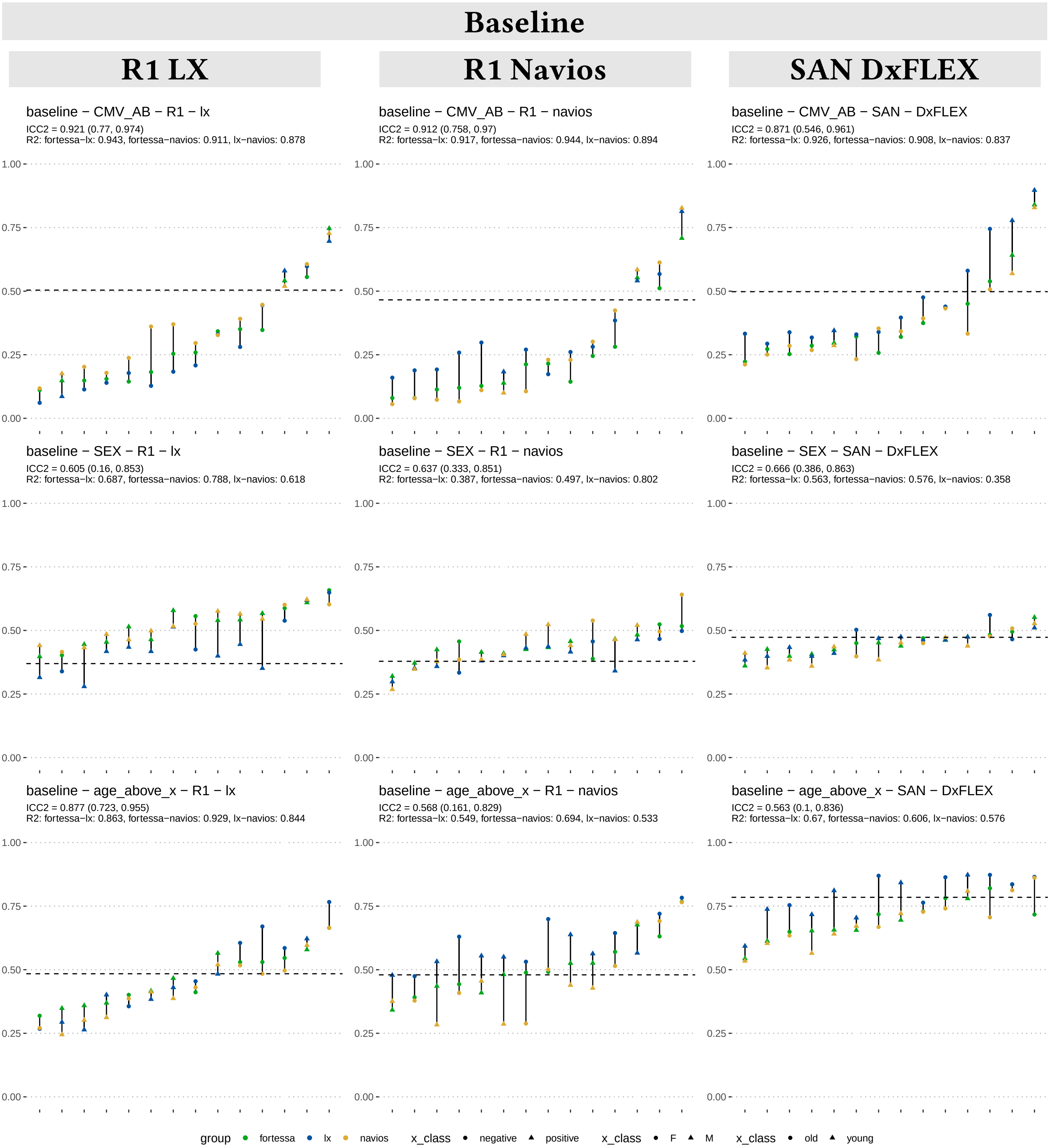

Supplementary Figure 22 *Predicted probabilities of age, sex and CMV-IgG seropositivity in the FORT cohort*

Supplementary Figure 22 *Predicted probabilities of age, sex and CMV-IgG seropositivity in the FORT cohort*

Predicted probabilities of CMV-IgG$^+$, sex and age above 30 based on the proportion of cells in each of 98 FlowSOM metaclusters. Models were trained on samples measured on either the Navios, LX, or DxFLEX cytometer and applied to FORT cohort samples measured on Fortessa, Navios LX cytometers. Each point represents a sample, with color indicating the measurement device. Triangles denote the respective positive class. Samples are ordered by the average probability across measurement-cytometers. The dashed line indicates the classification threshold derived from the ROC curve (minimum distance to the top-left corner) on the training cytometers' validation data. Titles indicate processing, outcome, training cohort and device. Subtitles report the intraclass correlation coefficient (ICC2) and the pairwise R2 values.

Supplementary Figure 23

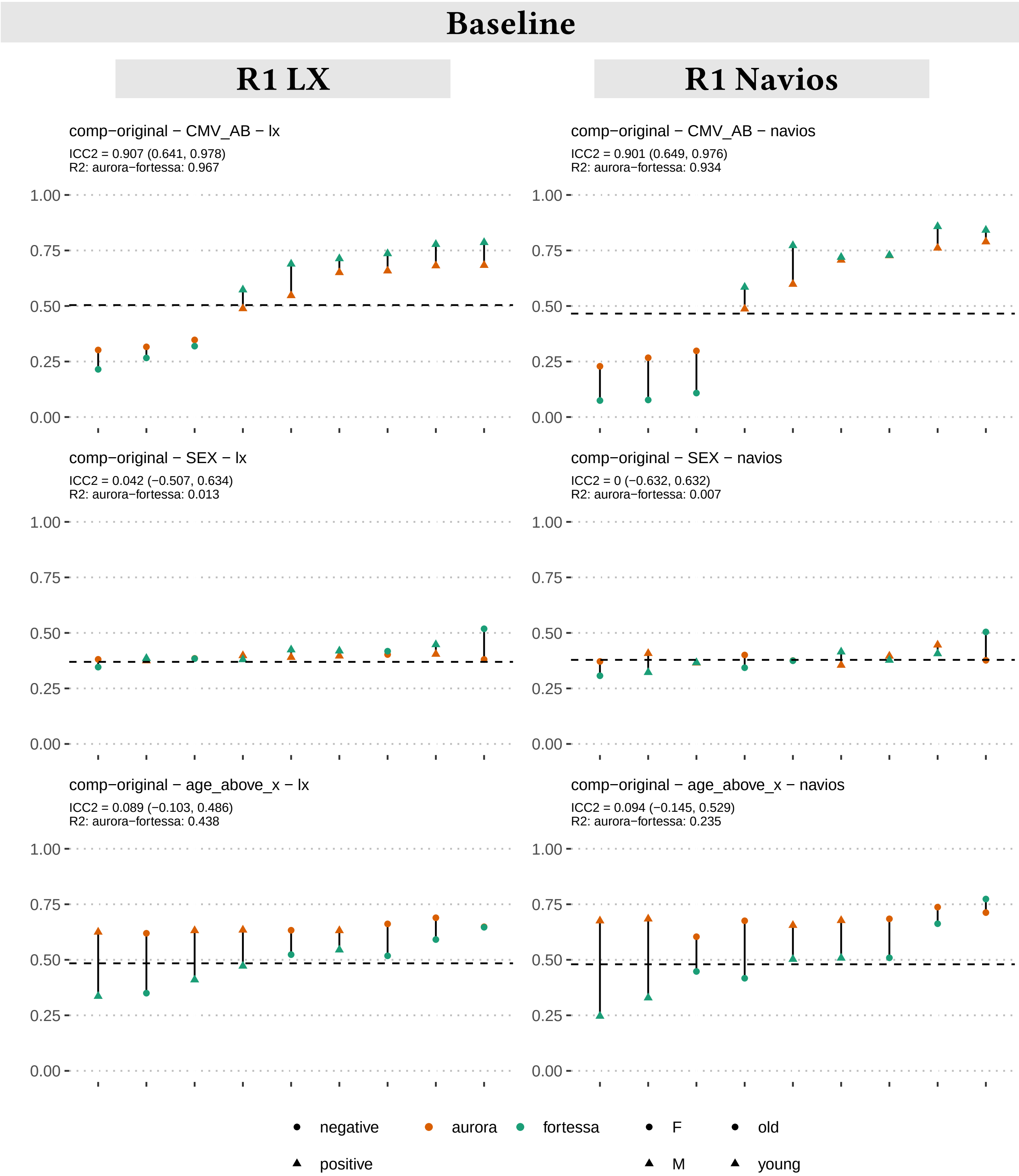

Supplementary Figure 23 *Predicted probabilities of age, sex and CMV-IgG seropositivity in the BAD cohort*

Supplementary Figure 23 *Predicted probabilities of age, sex and CMV-IgG seropositivity in the BAD cohort*

Predicted probabilities of CMV-IgG$^+$, sex and age above 30 based on the proportion of cells in each of 98 FlowSOM metaclusters. Models were trained on samples measured on either the Navios or LX cytometer and applied to SPECTRAL cohort samples measured on Fortessa and Aurora cytometers. Each point represents a sample, with color indicating the measurement device. Triangles denote the respective positive class. Samples are ordered by the average probability across measurement-cytometers. The dashed line indicates the classification threshold derived from the ROC curve (minimum distance to the top-left corner) on the training cytometers' validation data. Titles indicate processing, outcome, training cohort and device. Subtitles report the intraclass correlation coefficient (ICC2) and the pairwise R2 values.

Supplementary Figure 24

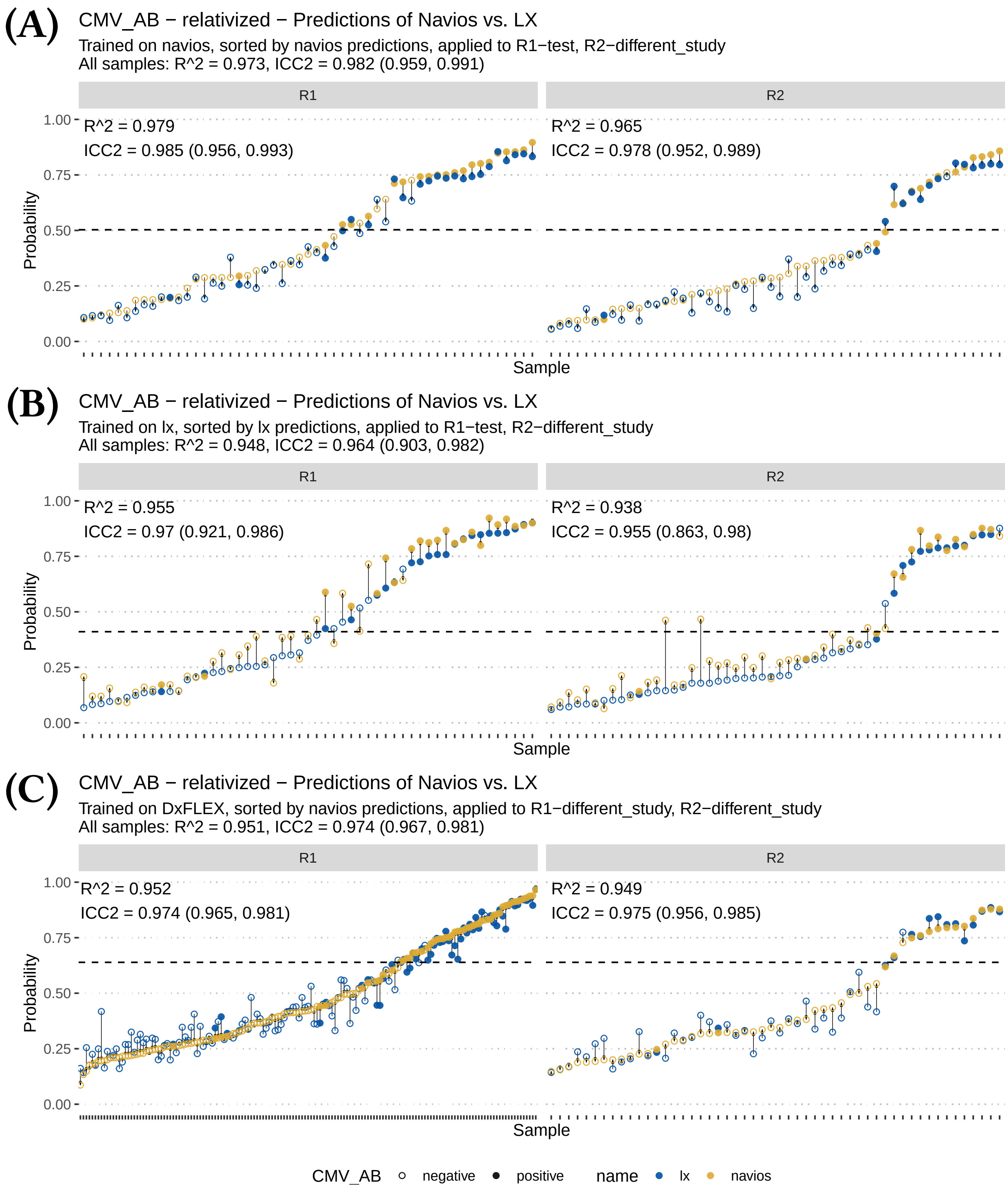

Supplementary Figure 24 *Predicted probabilities of CMV-IgG seropositivity after relativisation*

Supplementary Figure 24 *Predicted probabilities of CMV-IgG seropositivity after relativisation*

Predicted probabilities of CMV-IgG$^+$ based on the proportions of cells in each of 98 FlowSOM metaclusters after relativisation. Models were trained on samples measured with the Navios **(A)**, LX **(B)**, or DxFLEX **(C)** cytometer and applied to samples measured on both Navios and LX. Each point represents one sample; color indicates the cytometer. Filled circles correspond to CMV-IgG$^+$ samples, open circles to CMV-IgG$^-$ samples. Samples are ordered by predicted probability from the training-cytometer data predictions. The dashed line indicates the classification threshold, defined as the point on the ROC curve closest to the top-left corner using validation data. **Comparing to Supplementary Figure 19, we see that relativisation typically improves consistency in predicted probabilities across devices, supporting its utility in multi-instrument settings.**

Supplementary Figure 25

(A)

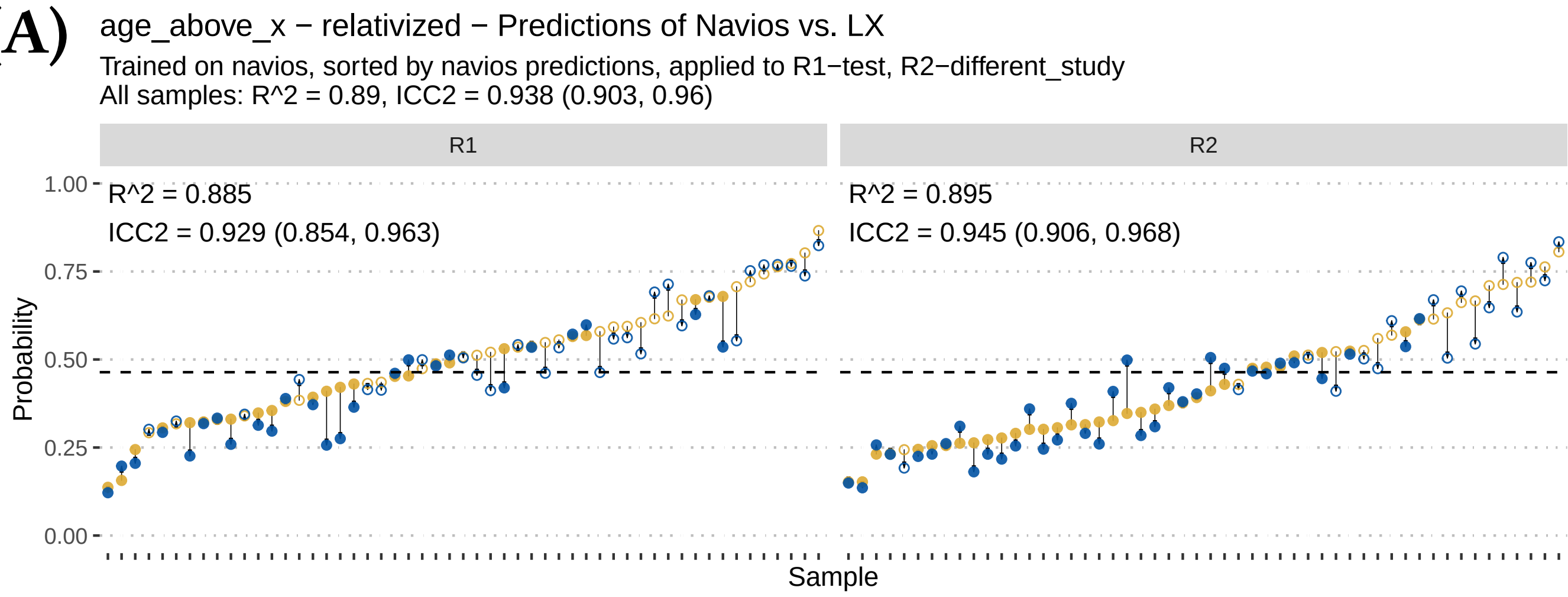

(B)

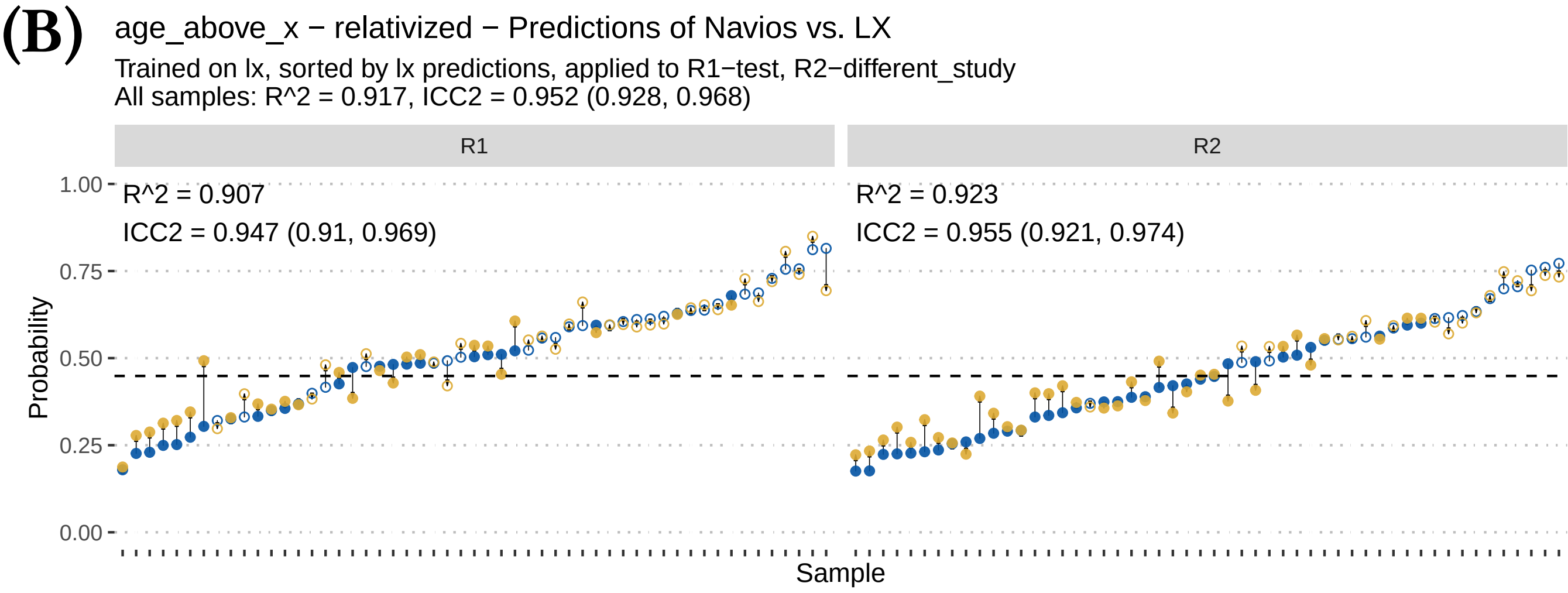

(C)

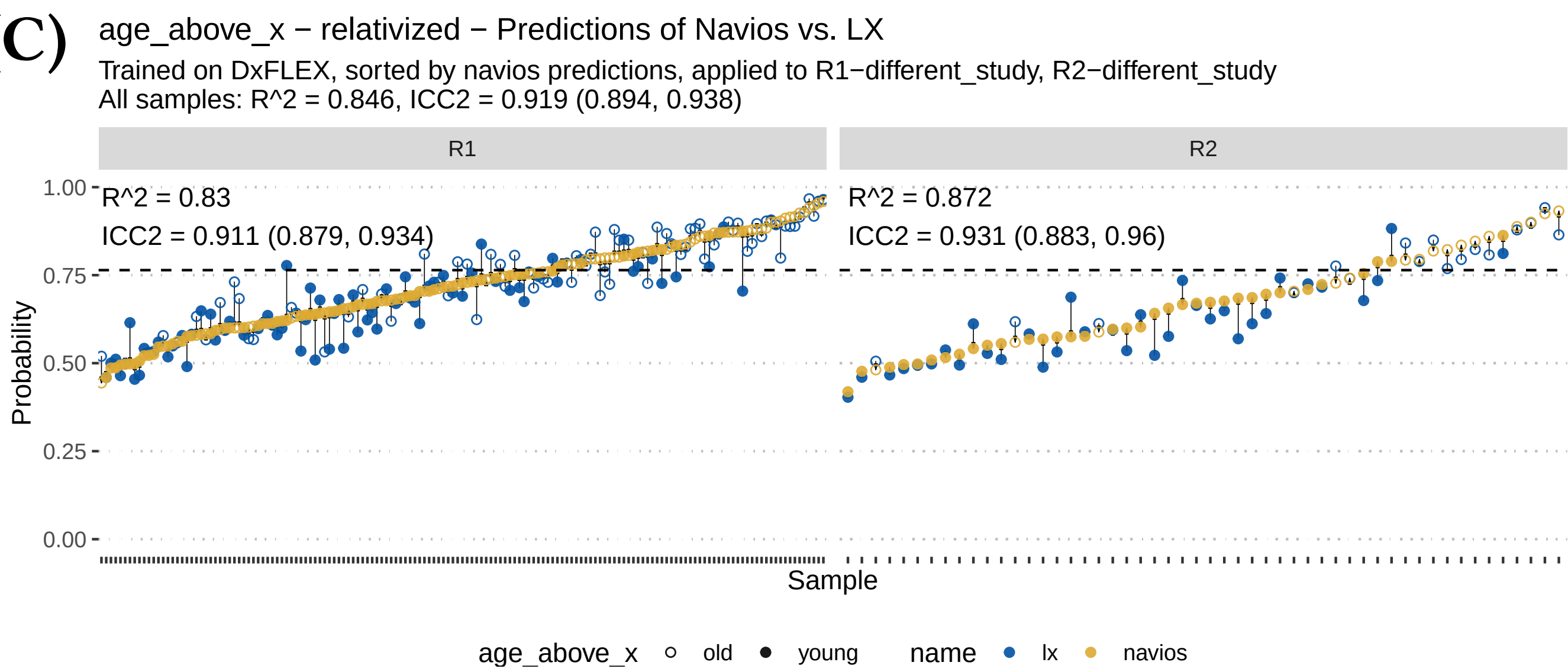

Supplementary Figure 25 *Predicted probabilities of age above 30 after relativisation*

Supplementary Figure 25 *Predicted probabilities of age above 30 after relativisation*

Predicted probabilities of age above 30 based on the proportions of cells in each of 98 FlowSOM metaclusters after relativisation. Models were trained on samples measured with the Navios **(A)**, LX **(B)**, or DxFLEX **(C)** cytometer and applied to samples measured on both Navios and LX. Each point represents one sample; color indicates the cytometer. Filled circles correspond to age up to 30 samples, open circles to above 30 samples. Samples are ordered by predicted probability from the training-cytometer data predictions. The dashed line indicates the classification threshold, defined as the point on the ROC curve closest to the top-left corner using validation data. **Comparing to Supplementary Figure 20, we see that relativisation typically improves consistency in predicted probabilities across devices, supporting its utility in multi-instrument settings.**

Supplementary Figure 26

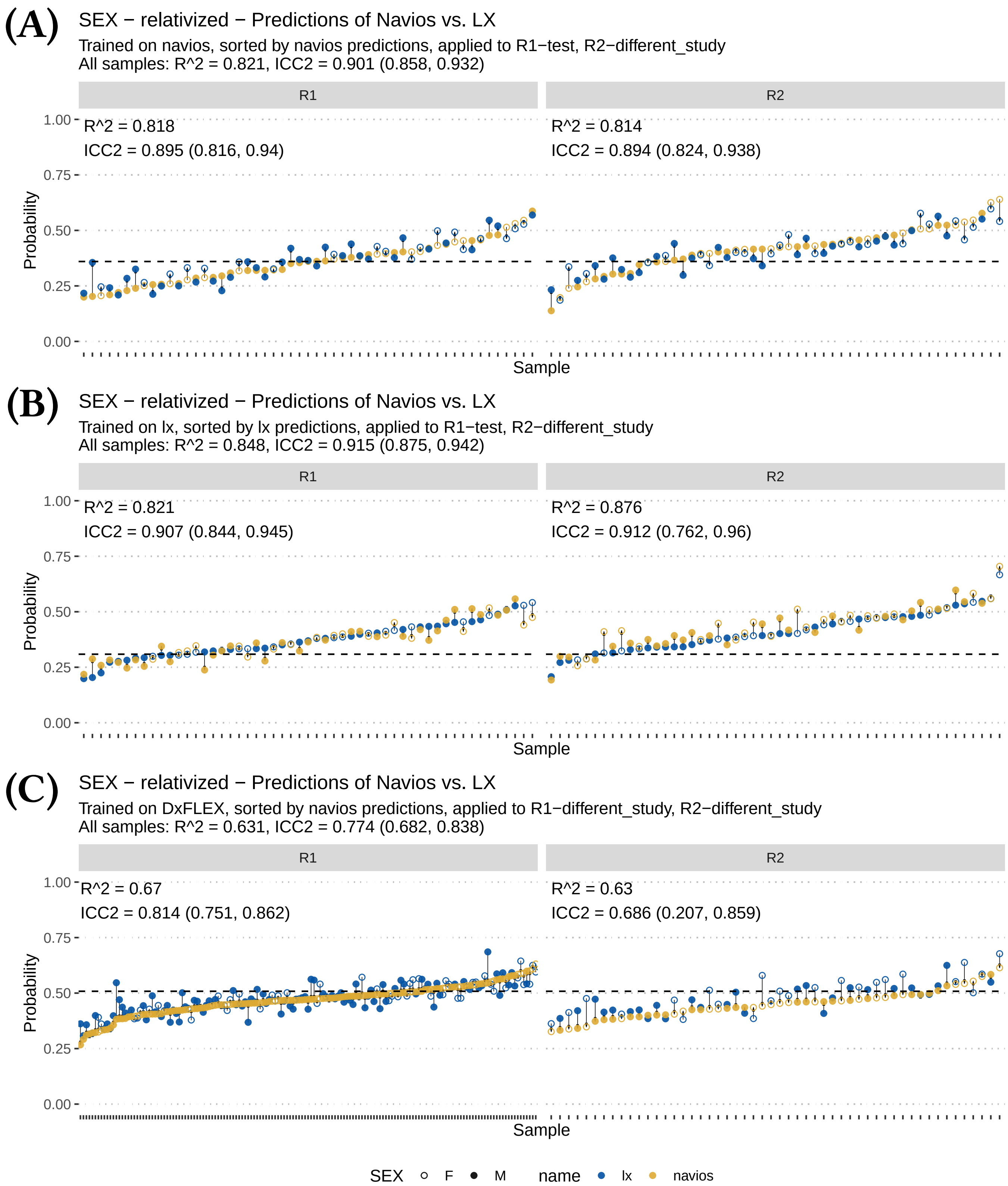

Supplementary Figure 26 *Predicted probabilities of sex after relativisation*

Supplementary Figure 26 *Predicted probabilities of sex after relativisation*

Predicted probabilities of sex based on the proportions of cells in each of 98 FlowSOM metaclusters after relativisation. Models were trained on samples measured with the Navios **(A)**, LX **(B)**, or DxFLEX **(C)** cytometer and applied to samples measured on both Navios and LX. Each point represents one sample; color indicates the cytometer. Filled circles denote male samples; open circles denote female samples. Samples are ordered by predicted probability from the training-cytometer data predictions. The dashed line indicates the classification threshold, defined as the point on the ROC curve closest to the top-left corner using validation data. **Comparing to Supplementary Figure 21, we see that, although overall model performance is poor, predictions are at least consistent across devices - an improvement over the unadjusted setting.**

Supplementary Figure 27

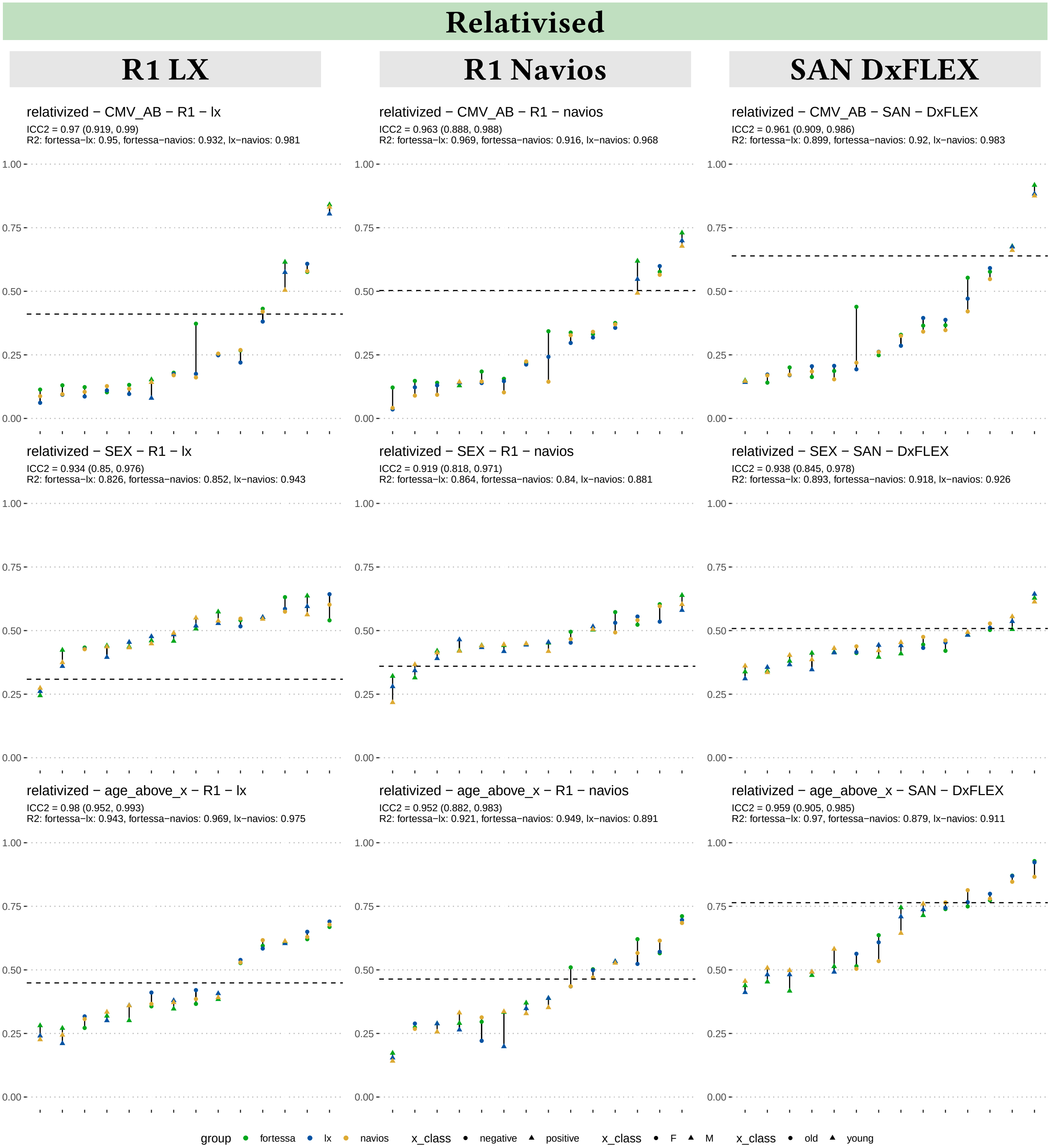

Supplementary Figure 27 *Predicted probabilities of age, sex and CMV-IgG serostatus on FORT cohort after relativisation.*

Supplementary Figure 27 *Predicted probabilities of age, sex and CMV-IgG serostatus on FORT cohort after relativisation.*

Predicted probabilities of positive CMV-IgG serostatus, sex and age above 30 based on the proportion of cells in each of 98 FlowSOM metaclusters following relativisation. Models were trained on samples measured on either the Navios, LX, or DxFLEX cytometer and applied to FORT cohort samples measured on Fortessa, Navios LX cytometers. Each point represents a sample, with color indicating the measurement device. Triangles denote the respective positive class. Samples are ordered by the average probability across measurement-cytometers. The dashed line indicates the classification threshold derived from the ROC curve (minimum distance to the top-left corner) on the training cytometers' validation data. Titles indicate processing, outcome, training cohort and device. Subtitles report the intraclass correlation coefficient (ICC2) and the pairwise R2 values. **Comparing to Supplementary Figure 22, relativisation improves consistency in predicted probabilities across devices, supporting its utility in multi-instrument settings.**

Supplementary Figure 28

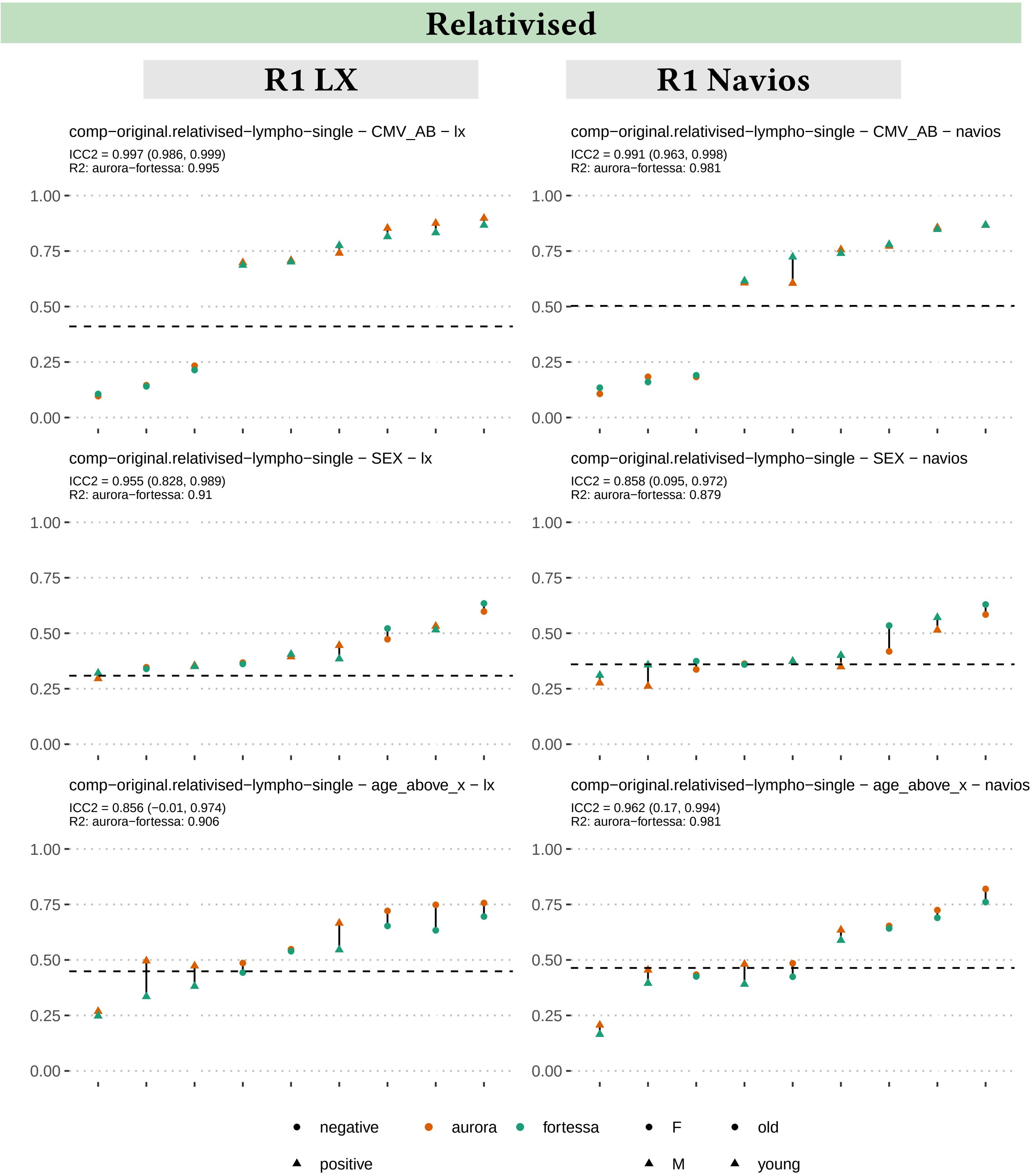

Supplementary Figure 28 *Predicted probabilities of age, sex and CMV-IgG seropositivity in the BAD cohort after relativisation.*

Supplementary Figure 28 *Predicted probabilities of age, sex and CMV-IgG seropositivity in the BAD cohort after relativisation.*

Predicted probabilities of positive CMV-IgG serostatus, sex and age above 30 based on the proportion of cells in each of 98 FlowSOM metaclusters following relativisation. Models were trained on samples measured on either the Navios or LX cytometer and applied to SPECTRAL cohort samples measured on Fortessa and Aurora cytometers.Each point represents a sample, with color indicating the measurement device. Triangles denote the respective positive class. Samples are ordered by the average probability across measurement-cytometers. The dashed line indicates the classification threshold derived from the ROC curve (minimum distance to the top-left corner) on the training cytometers' validation data. Titles indicate processing, outcome, training cohort and device. Subtitles report the intraclass correlation coefficient (ICC2) and the pairwise R2 values. **Comparing to Supplementary Figure 23, relativisation improves consistency in predicted probabilities across devices, supporting its utility in multi-instrument settings.**

Supplementary Figure 29

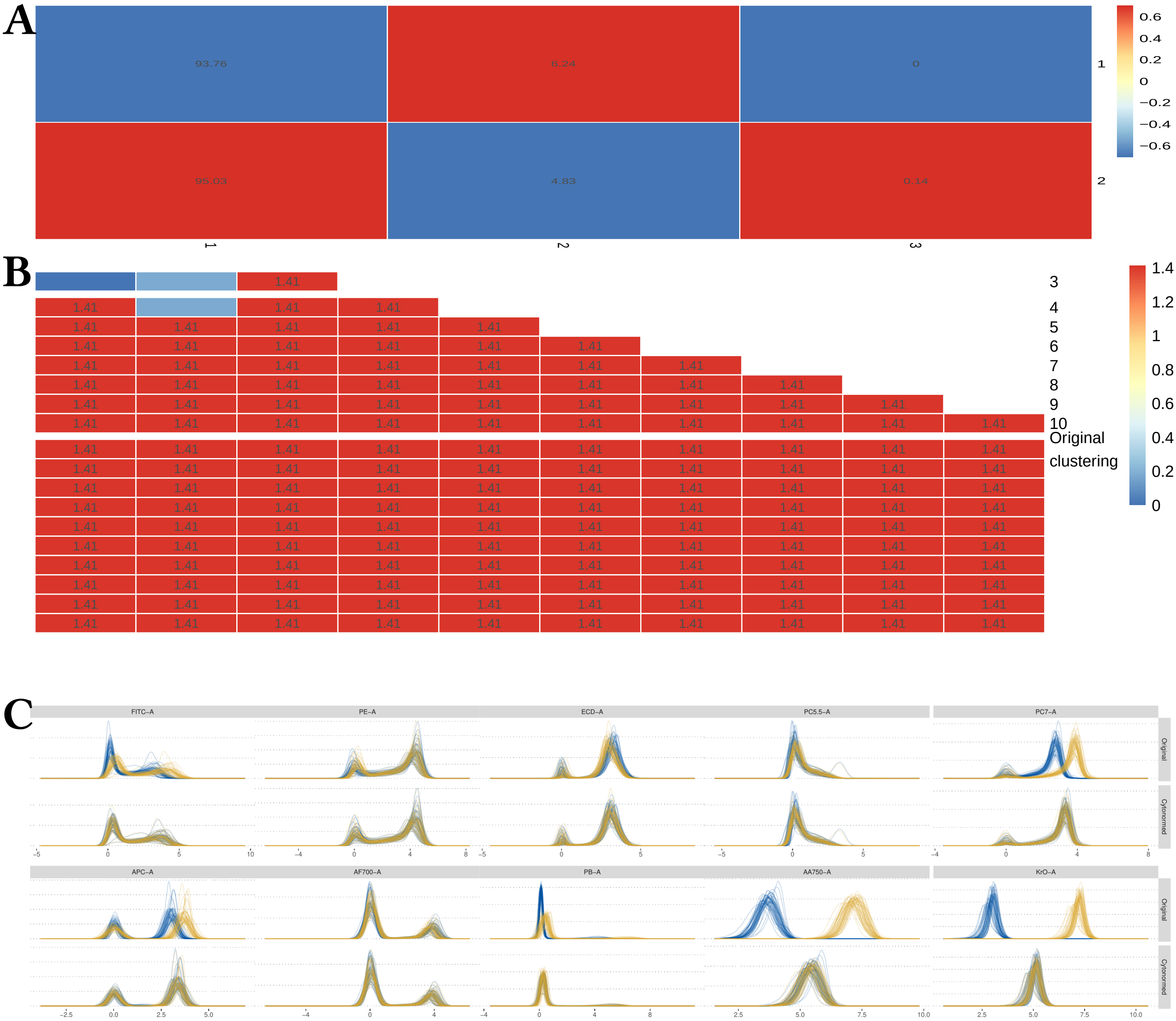

Supplementary Figure 29 *Performance of CytoNorm v2.0 under strong batch effects.*

Supplementary Figure 29 *Performance of CytoNorm v2.0 under strong batch effects.*
CytoNorm was applied to compensated, arcsinh-transformed training samples measured on both the Navios and LX cytometers. Although all samples were available from both instruments, we modeled the more realistic unpaired scenario: training samples from each cytometer were aggregated separately, and normalization was performed across devices using these cytometer-specific aggregates. Clustering within CytoNorm was performed using 100 clusters and 3 metaclusters. Higher numbers of metacluster resulted in failed alignment due to the absence of shared cellular populations across cytometers - only one or no metacluster contained overlapping cells. Given the pronounced batch effects observed in the PCA analysis (Figure 2, Supplementary Figure 30), it is not surprising that metaclustering shown here violated CytoNorm's core assumption of batch-invariant metaclusters. **(A)** Distribution of cells across the three metaclusters in the aggregated Navios and LX training samples. Only metaclusters '1' and '2' contains cells from both cytometers, most cells are in metacluster '1'. **(B)** Coefficient of variation (CV) in metacluster cell proportions between aggregates, plotted per metacluster (x-axis) and across varying metacluster resolutions (y-axis). A CV of $\sqrt{2}$ indicates complete absence from one aggregate. The lower panel, shows CVs for the original 400 clusters. **(C)** Density distributions of arcsinh-transformed $CD3^+$ cells per training sample, before and after normalization, for Navios and LX. Alignment is driven almost exclusively by the single shared metacluster '1', effectively collapsing CytoNorm into global quantile normalization. This compromises CytoNorm's core strength, aligning samples within biologically meaningful metaclusters and risks distorting biological structure.

Supplementary Figure 30

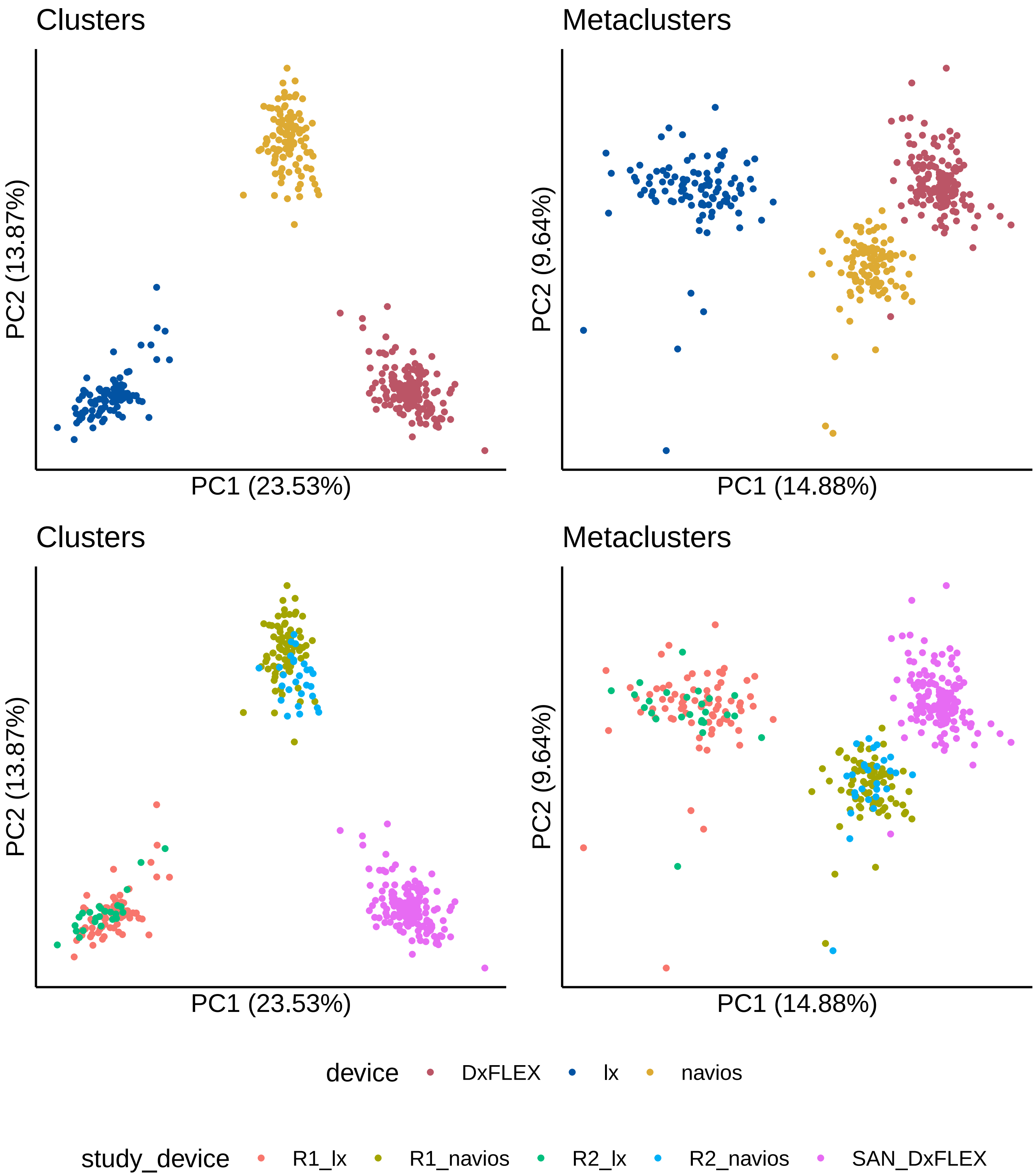

Supplementary Figure 30 *PCA plots coloured by R1-Navios, R1-LX, R2-Navios, R2-LX*

Supplementary Figure 30 *PCA plots coloured by R1-Navios, R1-LX, R2-Navios, R2-LX*

Principal component analysis (PCA) of the cell proportion matrix reveals cytometer-specific patterns. Shown are the first two principal components computed from the proportions of cells per cluster (left) and per metacluster (right). Each point represents a sample and is colored by cytometer (top row) or by cytometer and study (bottom row). The data demonstrate distinct separation between cytometers, while samples from studies R1 and R2 remain intermixed within each cytometer, suggesting that inter-cytometer variability exceeds inter-study variability.

Supplementary Figure 31

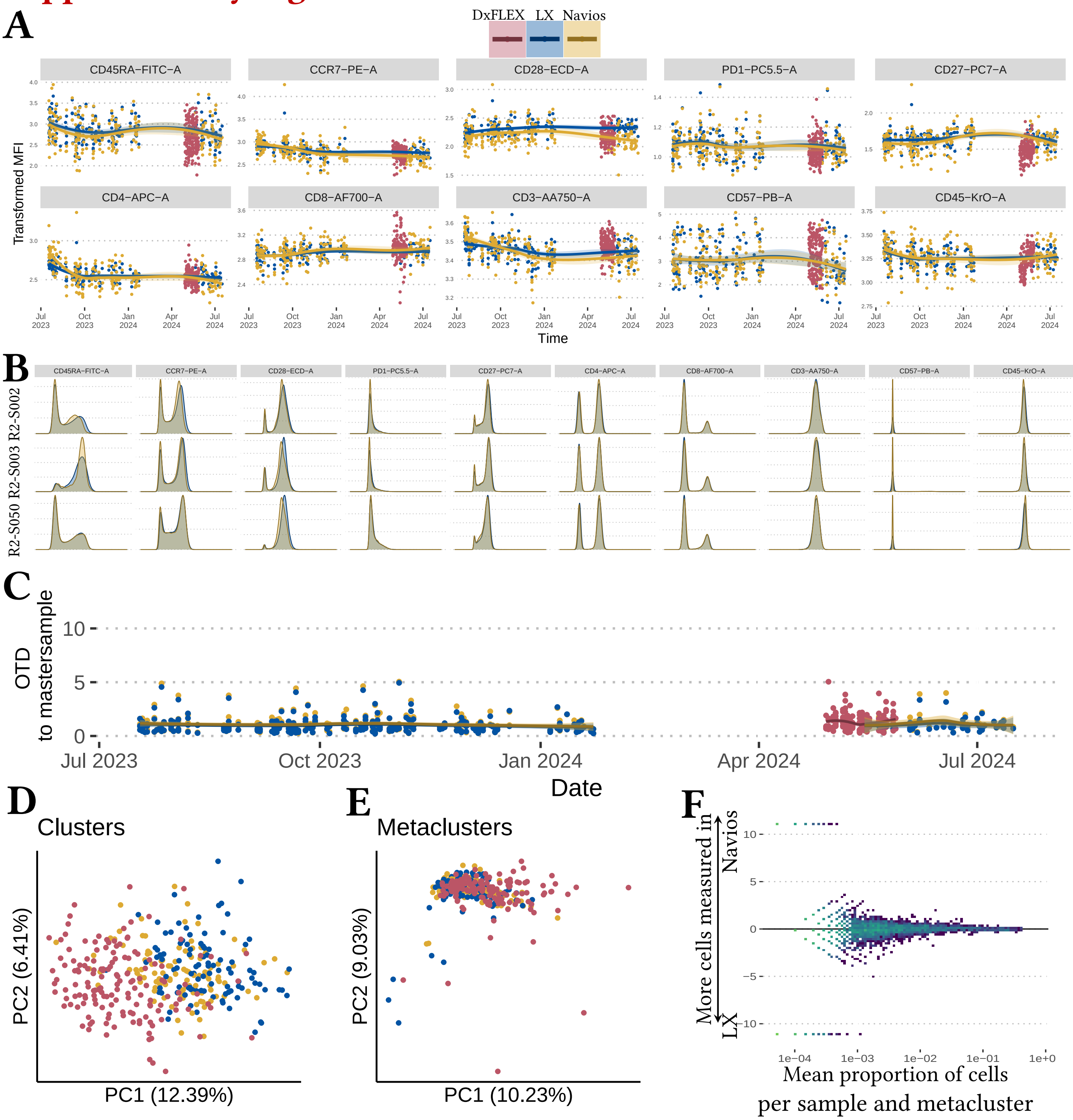

Supplementary Figure 31 *Cytonorm after CD3 relativisation does not improve alignment over relativisation alone.*

Supplementary Figure 31 *Cytonorm after CD3 relativisation does not improve alignment over relativisation alone.*

All plots have been shown previously in Figure 2 for the same samples but without relativisation. Here, each sample was first relativised according to its own single staining control as described in Figure 4. Then, CytoNorm (v2) was performed with an aggregated sample of all training samples measured on the navios. This trained CytoNorm was then applied sample-by-sample to all samples and the results are shown here. **(A)** Longitudinal flow cytometry analysis of surface marker expression on immune cell populations. Each subplot displays the mean fluorescence intensity of a specific marker over time, calculated on different manually gated cell subsets. The y-axis represents the fluorescence intensity after arcsinh transformation. Each point represents a single sample, colored by the device used for measurement. Solid lines and shaded areas are LOESS estimates and confidence intervals. **(B)** Density plots for three samples of the R2 cohort. Each subplot displays the density of the arcsinh transformed fluorescence intensity of a specific marker. The x-axis represents the fluorescence intensity, and the y-axis represents the density. Color represents the measurement device. **(C)** We used OTD to summarize the differences across all markers. The plot shows the OTD to a mastersample of 10,000 cells randomly subsampled from all training samples of R1 (each sample either on Navios™ or CytoFLEX LX™) and SAN cohorts (DxFLEX™). The y-axis represents the OTD value which is larger for more dissimilar samples. Solid lines and shaded areas are LOESS estimates and confidence intervals. **(D)** PCA of number of cells in each cluster of first presentations of all cohorts after FlowSOM built on training samples only. Each point represents a sample colored by the measurement device. **(E)** PCA of number of cells in each metacluster of first presentations of all cohorts after FlowSOM built on training samples only. Each point represents a sample colored by the measurement device. **(F)** Bland-Altman plot of the number of cells in each metacluster comparing every sample measured on Navios™ and CytoFLEX LX™ cytometers. First presentation samples of R1 and R2. FlowSOM was generated on training samples only and applied to all samples. The x-axis represents the average number of cells in each metacluster, and the y-axis represents the log2-ratio between the number of cells in each metacluster. The color represents the amount of sample-metacluster values at that position.

Supplementary Figure 32

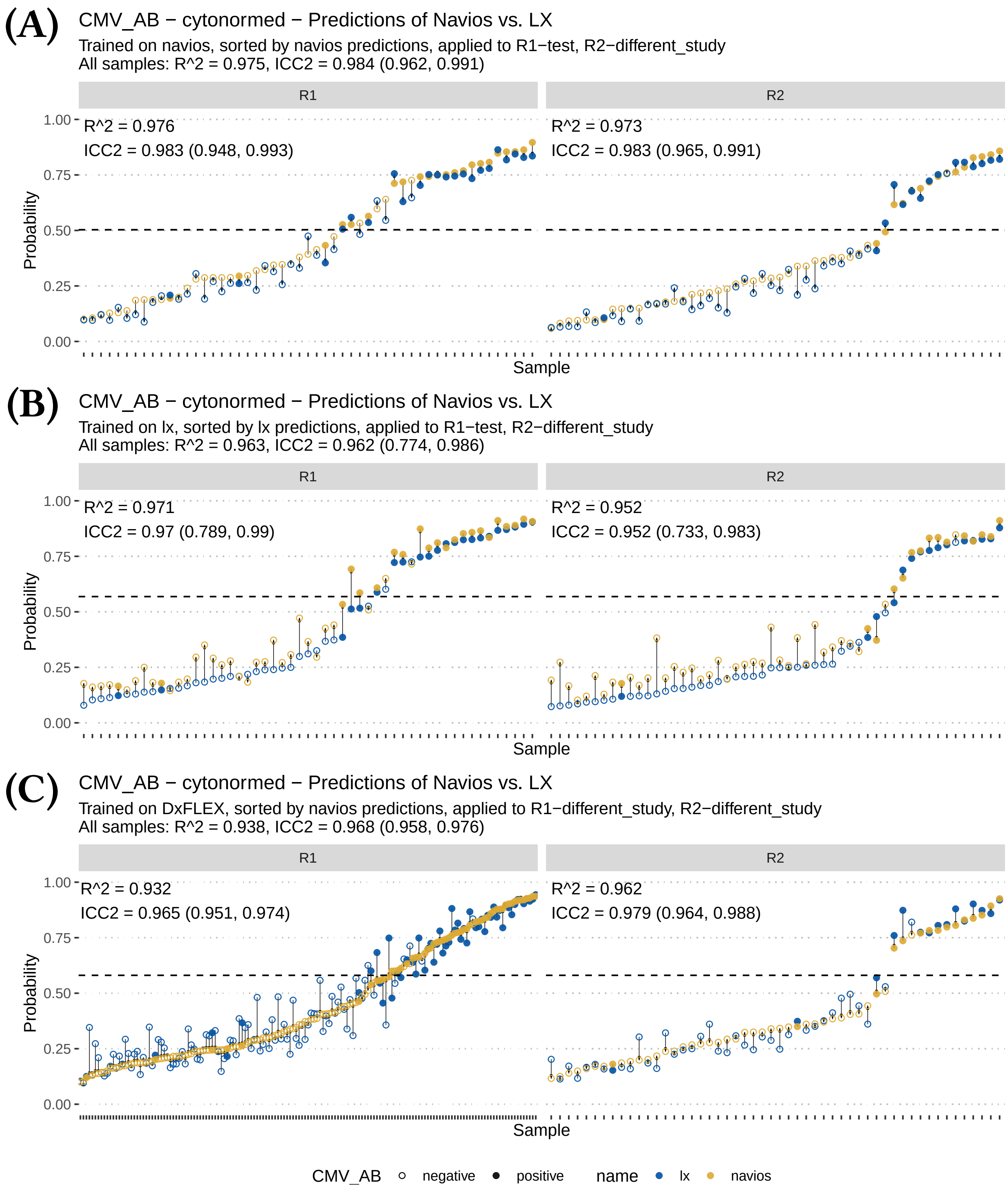

Supplementary Figure 32 *Predicted probabilities of CMV-IgG seropositivity after relativisation and CytoNorm*

Supplementary Figure 32 *Predicted probabilities of CMV-IgG seropositivity after relativisation and CytoNorm*

Predicted probabilities of CMV-IgG$^+$ based on the proportions of cells in each of 98 FlowSOM metaclusters after relativisation plus CytoNorm. Models were trained on samples measured with the Navios **(A)**, LX **(B)**, or DxFLEX **(C)** cytometer and applied to samples measured on both Navios and LX. Each point represents one sample; color indicates the cytometer. Filled circles correspond to CMV-IgG$^+$ samples, open circles to CMV-IgG$^-$ samples. Samples are ordered by predicted probability from the training-cytometer data predictions. The dashed line indicates the classification threshold, defined as the point on the ROC curve closest to the top-left corner using validation data. **Comparing to Supplementary Figure 24, overall ICC2 is on par to relativisation alone.**

Supplementary Figure 33

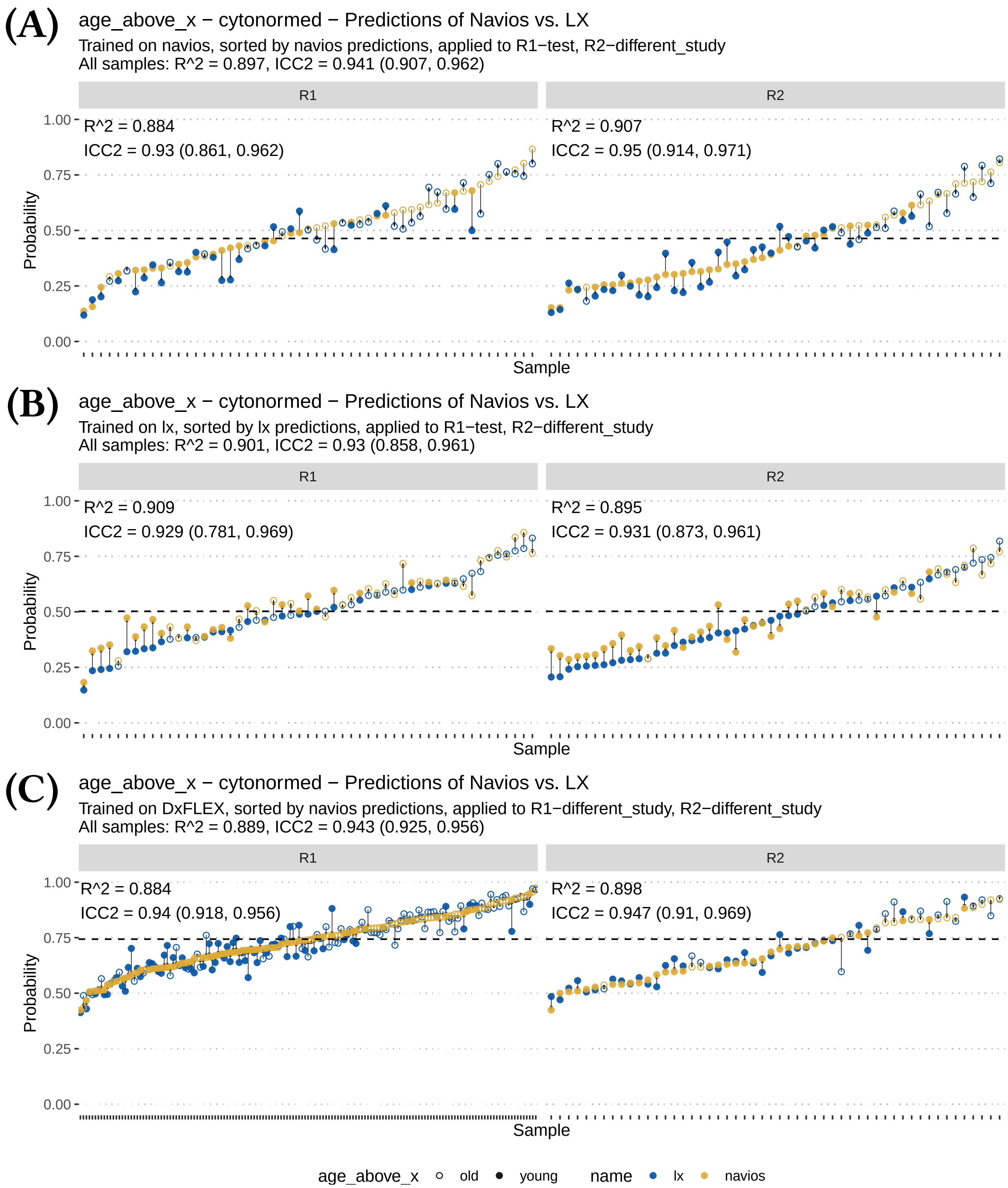

Supplementary Figure 33 *Predicted probabilities of age above 30 after relativisation and CytoNorm*

Supplementary Figure 33 *Predicted probabilities of age above 30 after relativisation and CytoNorm*

Predicted probabilities of age above 30 based on the proportions of cells in each of 98 FlowSOM metaclusters after relativisation. Models were trained on samples measured with the Navios **(A)**, LX **(B)**, or DxFLEX **(C)** cytometer and applied to samples measured on both Navios and LX. Each point represents one sample; color indicates the cytometer. Filled circles correspond to age up to 30 samples, open circles to above 30 samples. Samples are ordered by predicted probability from the training-cytometer data predictions. The dashed line indicates the classification threshold, defined as the point on the ROC curve closest to the top-left corner using validation data. **Comparing to Supplementary Figure 25, we see that there is no improvement in ICC2 after relativisation alone.**

Supplementary Figure 34

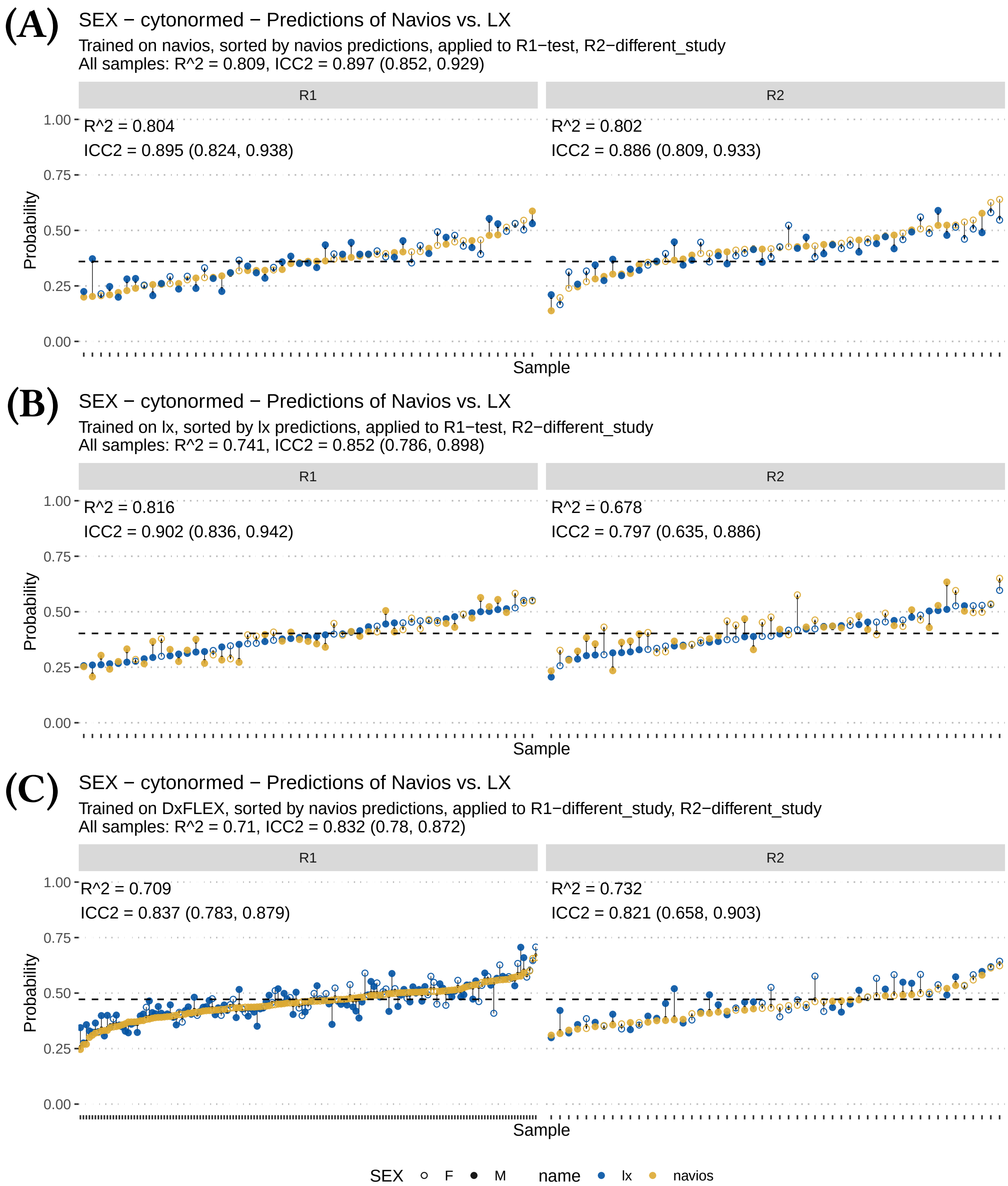

Supplementary Figure 34 *Predicted probabilities of sex after relativisation and CytoNorm*

Supplementary Figure 34 *Predicted probabilities of sex after relativisation and CytoNorm*

Predicted probabilities of sex based on the proportions of cells in each of 98 FlowSOM metaclusters after relativisation. Models were trained on samples measured with the Navios **(A)**, LX **(B)**, or DxFLEX **(C)** cytometer and applied to samples measured on both Navios and LX. Each point represents one sample; color indicates the cytometer. Filled circles denote male samples; open circles denote female samples. Samples are ordered by predicted probability from the training-cytometer data predictions. The dashed line indicates the classification threshold, defined as the point on the ROC curve closest to the top-left corner using validation data. **Comparing to Supplementary Figure 26, we see that ICC2 decreased compared to relativisation alone.**

Supplementary Figure 35

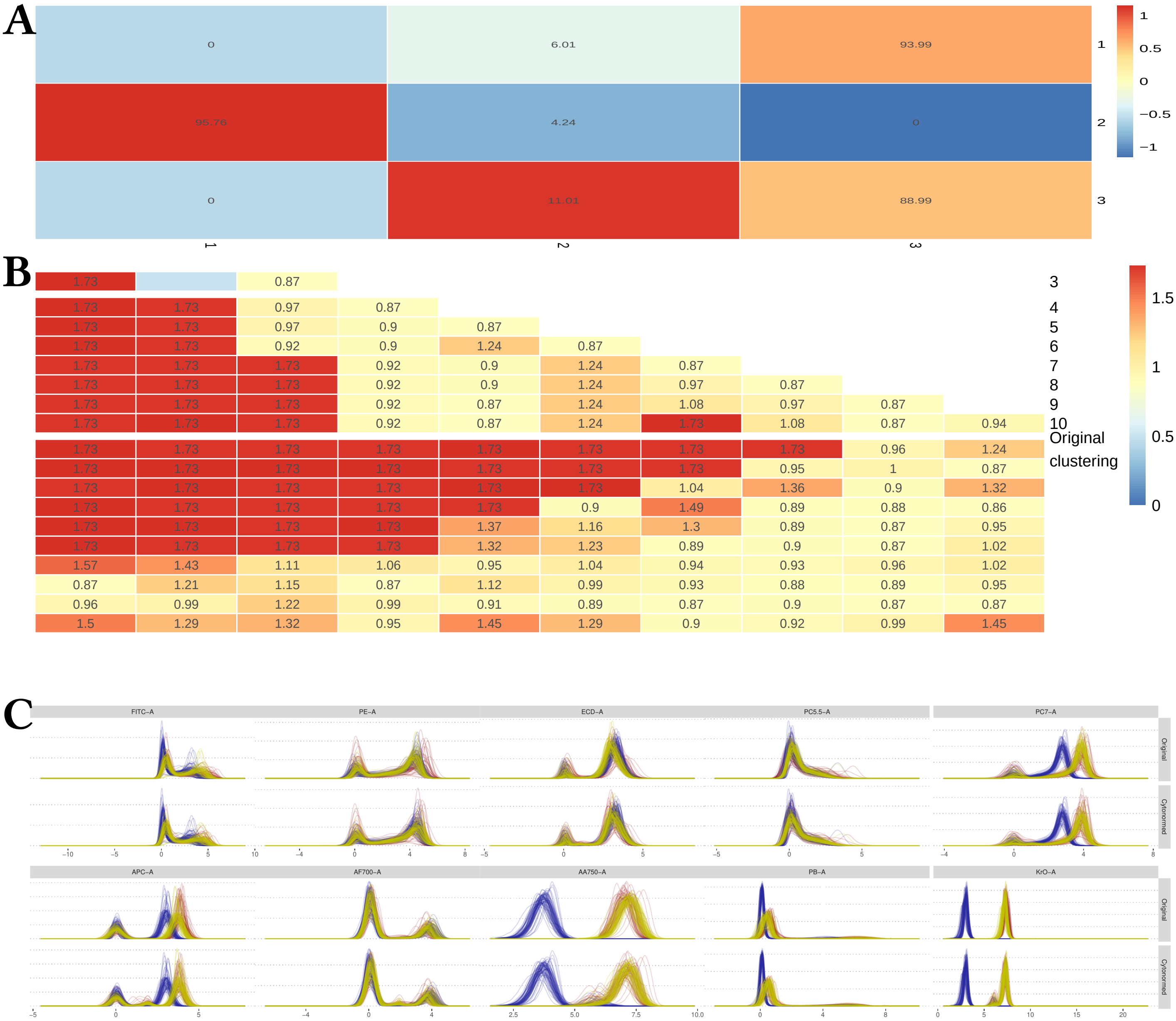

Supplementary Figure 35 *Performance of CytoNorm v2.0 under strong batch effects with the ICU cohort.*

Supplementary Figure 35 *Performance of CytoNorm v2.0 under strong batch effects with the ICU cohort.* CytoNorm was applied to compensated, arcsinh-transformed training samples measured on both the Navios and LX cytometers. Although all samples were available from both instruments, we modeled the more realistic unpaired scenario: training samples from each cytometer were aggregated separately, and normalization was performed across devices using these cytometer-specific aggregates. For the ICU cohort, 60% of the samples were used as training samples and modelled as its own batch even if they were also measured on the Navios with identical settings. Clustering within CytoNorm was performed using 100 clusters and 3 metaclusters. Even with this extremely low number of metaclusters, the alignment failed due to severely different compositions between the Navios and LX cytometers - the LX and Navios had almost no overlapping cells in the metaclusters. **(A)** Distribution of cells across the three metaclusters (x axis) in the aggregated Navios, ICU-Navios and LX training samples. Only metacluster '2' contains cells from both cytometers (4.24 - 11.01%), but most cells 95% are unique to either Navios or LX. **(B)** Coefficient of variation (CV) in metacluster cell proportions between aggregates, plotted per metacluster (x-axis) and across varying metacluster resolutions (y-axis). A CV of $\sqrt{3}$ (3 cytometers) indicates complete absence from two of three aggregates. The lower panel, shows CVs for the original 100 clusters. **(C)** Density distributions of arcsinh-transformed $CD3^+$ cells per training sample, before and after normalization, for Navios (yellow), LX (blue) and ICU-Navios (red). Only 10% of the cells are shared across all 3 clusters, prohibiting successfull alignment by CytoNorm.

Supplementary Figure 36

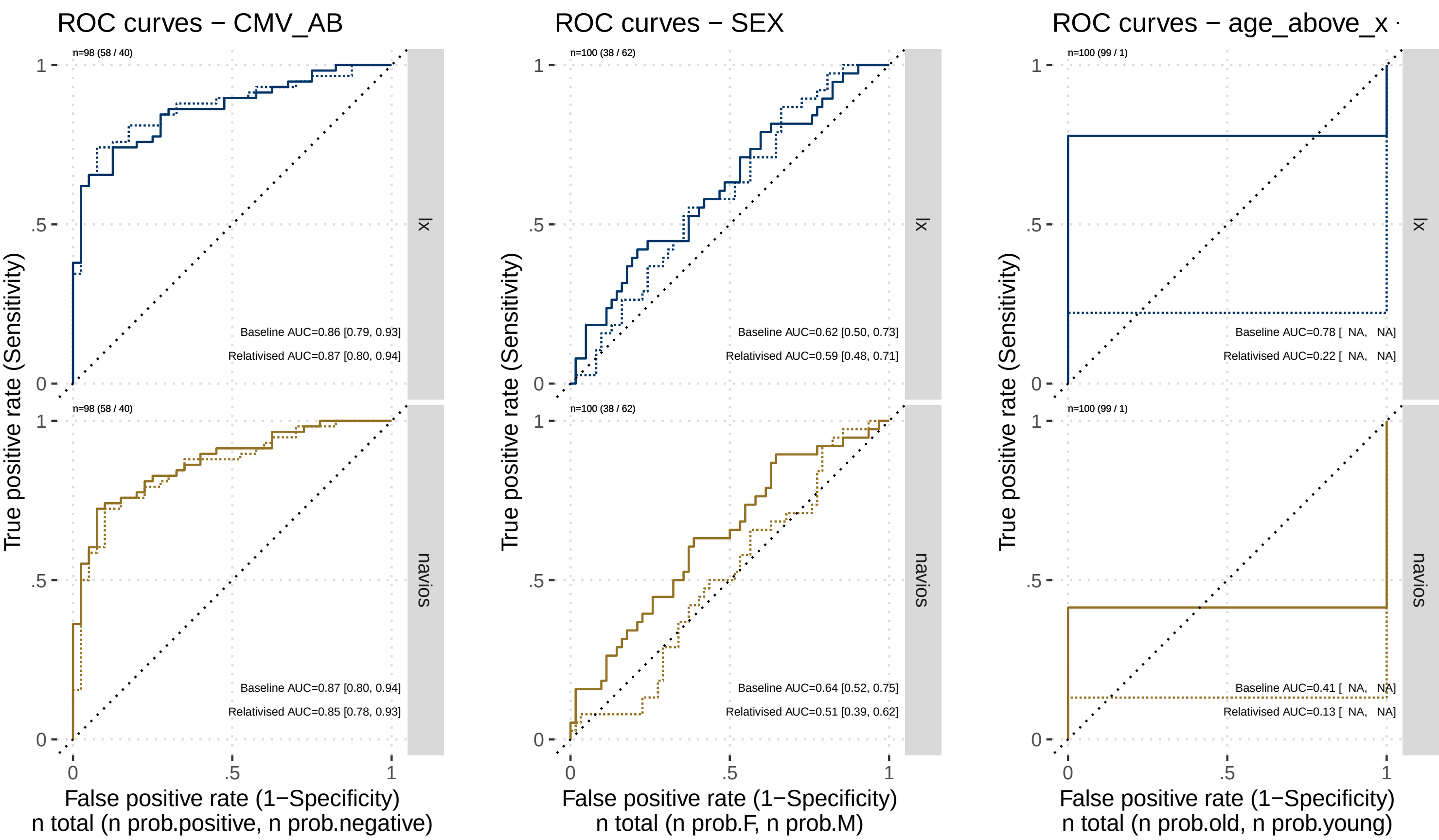

Supplementary Figure 36 *AUROC curves for age, sex and CMV-IgG seropositivity in the ICU cohort*

ROC curves for CMV-IgG^{+}, sex and age above 30 based on the proportion of cells in each of 98 FlowSOM metaclusters. Models were trained on samples measured on either the Navios or LX cytometer and applied to ICU cohort samples measured on Navios cytometer. Colour denotes the device where the model was trained on, dotted lines indicate relativised, solid lines baseline processing. Titles indicate outcome. AUROCs for age are unreliable after only a single patient was below 30 years of age in this ICU cohort.

Supplementary Figure 37

Baseline

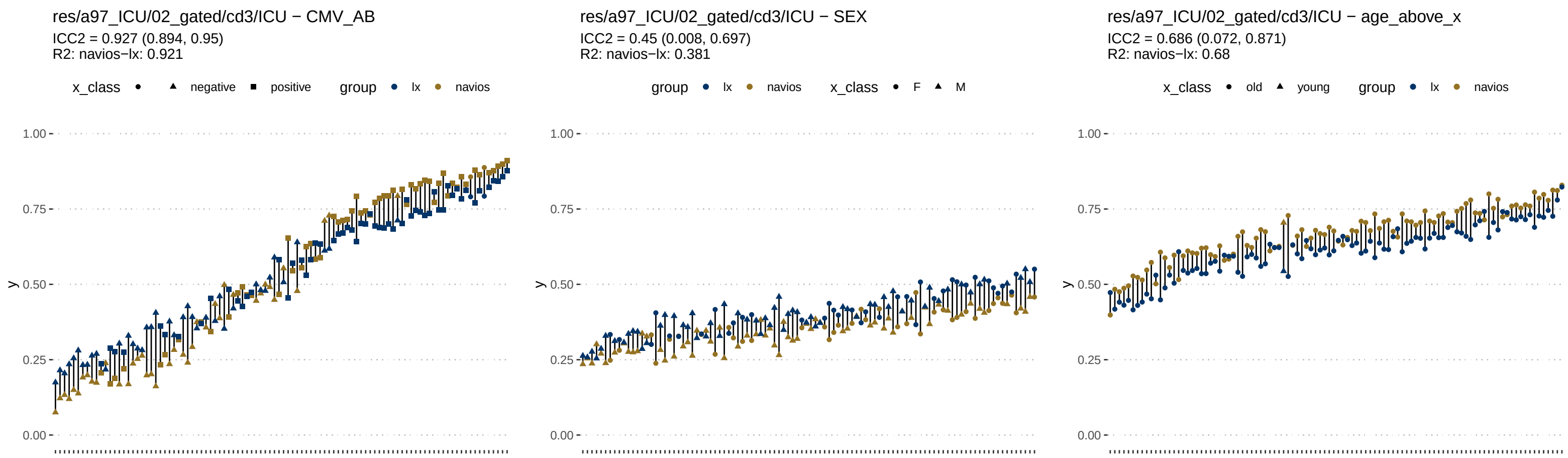

Relativised

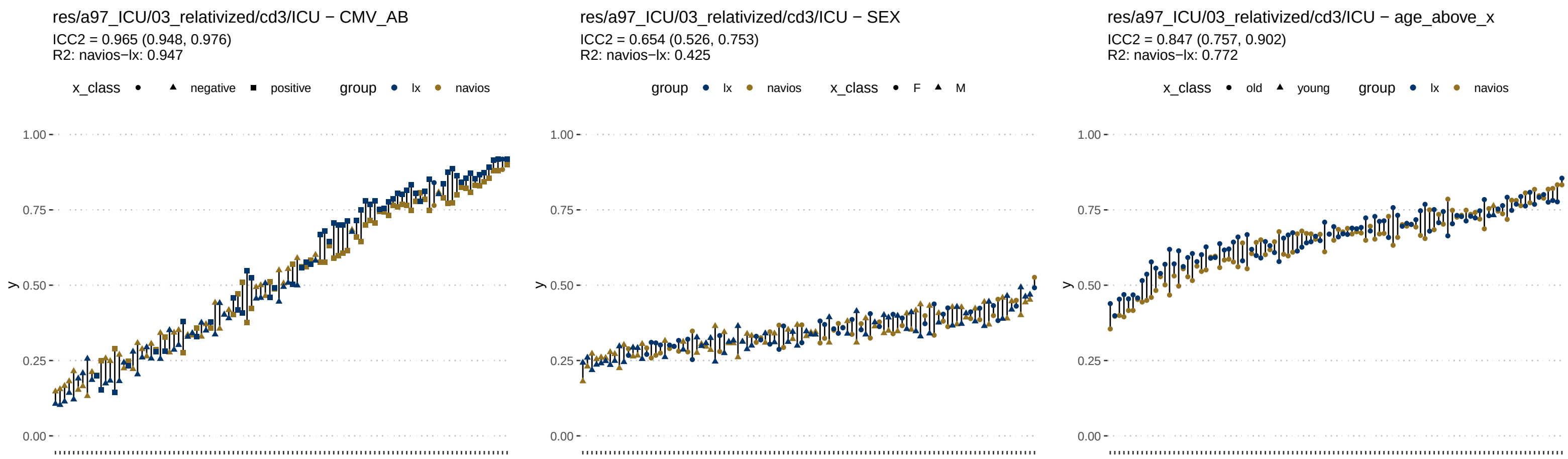

Supplementary Figure 37 *Predicted probabilities of age, sex and CMV-IgG seropositivity in the ICU cohort*

Predicted probabilities of CMV-IgG$^+$, sex and age above 30 based on the proportion of cells in each of 98 FlowSOM metaclusters. Models were trained on samples measured on either the Navios or LX cytometer and applied to ICU cohort samples measured on Navios cytometer. Each point represents a sample, with color indicating the device where the model was trained on. Samples are ordered by the average probability across training-cytometers. Titles indicate processing and outcome. Subtitles report the intraclass correlation coefficient (ICC2) and the pairwise R2 values comparing predictions from Navios- or LX- trained models.

Supplementary Table 1

	488 nm					633 nm		405 nm		
	FL-1<br>FITC	FL-2<br>PE	FL-3<br>ECD	FL-4<br>PC5.5	FL-5<br>PC7	FL-6<br>APC	FL-7<br>AF700	FL-8<br>AA750	FL-9<br>PB	FL-10<br>KrO
**ANTIGEN**	CD45RA	CCR7 (CD197)	CD28	PD1 (CD279)	CD27	CD4	CD8	CD3	CD57	CD45

Supplementary Table 1 DuraClone T cell subsets Tube

Supplementary Table 2

	488 nm					633 nm		405 nm		
	FL-1 FITC	FL-2 PE	FL-3 ECD	FL-4 PC5.5	FL-5 PC7	FL-6 APC	FL-7 AF700	FL-8 AA750	FL-9 PB	FL-10 KrO
**ANTIGEN**	CD3	CD3	CD3	CD3	CD3	CD3	CD3	CD3	CD3	CD3
**Clone name**	UCHT1	UCHT1	UCHT1	UCHT1	UCHT1	UCHT1	UCHT1	UCHT1	UCHT1	UCHT1
**Isotype**	mIgG1	mIgG1	mIgG1	mIgG1	mIgG1	mIgG1	mIgG1	mIgG1	mIgG1	mIgG1
**Amount (µl)**	14	20	10	4	5	9	3	5	9	8
**Supplier**	BC	BC	BC	BC	BC	BC	BioLegend	BC	BC	BC
**Cat. #**	A07746	A07747	A07748	B49203	737657	IM2467	300424	A94680	B49204	B00068
**Status**	IVD	IVD	IVD	IVD	IVD	IVD	RUO	IVD	IVD	ASR
**µg/ml**	25	3	20	100	50	12.5	500	100	100	100

Supplementary Table 2 Single stain tube concentrations.

Supplementary Table 3

Supplementary Table 3 Cofactors before and after rescaling for all cytometers. Cofactors were determined on panel samples by a blinded, experienced cytometrist.

Channel	Navios	DxFLEX	LX
FITC-A	1000	4000	5000
PE-A	2000	5000	10 000
ECD-A	2000	5500	10 000
PC5.5-A	3000	7500	15 000
PC7-A	2500	6000	10 000
APC-A	2000	10 000	9000
AF700-A	4000	10 000	9000
AA750-A	100	1000	2000
PB-A	500	4000	2000
KrO-A	50	2000	2000

Asinh cofactors used before rescaling per cytometer.

Channel	Panel
FITC-A	300
PE-A	500
ECD-A	300
PC5.5-A	250
PC7-A	1000
APC-A	500
AF700-A	2500
AA750-A	500
PB-A	1500
KrO-A	5000

Asinh cofactors used after rescaling.

$$\text{arcsinh}(x, cofactor_X) := \text{arsinh}(\frac{x}{cofactor_X}) := \text{arsinh}(y) = ln(y + \sqrt{y^2 + 1})$$

Supplementary Tables 4 and 5

All FCS files follow a naming structure as the following example:
`R2_p052_s054_raw_ungated_none_Inf_navios_01-CD3-FITC.fcs`

**Field**	**Example**	**Definition**
Cohort	R2	Regensburg cohort 2
Donor	p052	Donor 52
Sample	s054	Sample 54
Processing	raw	Raw data
Applied gating	ungated	No gating applied
Subsample method	none	No subsampling applied
Subsample size	Inf	All measured cells were subsampled (Infinity)
Device	navios	Navios cytometer
sampleNR-Antigen-fluorochrome	01-CD3-FITC	First cytometry-sample; CD3 stained with FITC

Supplementary Table 4 Definitions of fields in file naming structure.

**sampleNR-Antigen-fluorochrome**	**Definition**
01-CD3-FITC	Single-stained CD3 with FITC
02-CD3-PE	Single-stained CD3 with PE
03-CD3-ECD	Single-stained CD3 with ECD
04-CD3-PC5.5	Single-stained CD3 with PC5.5
05-CD3-PC7	Single-stained CD3 with PC7
06-CD3-APC	Single-stained CD3 with APC
07-CD3-AF700	Single-stained CD3 with AF700
08-CD3-AA750	Single-stained CD3 with AA750
09-CD3-PB	Single-stained CD3 with PB
10-CD3-KrO	Single-stained CD3 with KrO
11-none	No staining
12-panel	DURAClone IM T Cell Subsets Tube
13-01..11-SingleNone	All single-stain controls and the unstained control concatenated in one file

Supplementary Table 5 Sample stainings and their definitions.

Supplementary Table 6

Supplementary Table 6 Label descriptions of the data.

ShortName	LongName
DATE	DATE
AGE	AGE
SEX	SEX
ABO	ABO status
RHD	Rhesus (D) status
Kell	Kell status
IABS	Irregular blood group antibody screen
HCV_PCR	Hepatitis C Virus (HCV) PCR
HIV_PCR	Human Immunodeficiency Virus (HIV)-1/2 PCR
HBV_PCR	Hepatitis B Virus (HBV) PCR
HEV_PCR	Hepatitis E Virus (HEV) PCR
WNV_PCR	West Nile Virus (WNV) PCR
A_AB	Anti-A
B_AB	Anti-B
WBC	Leucocytes (c/nl)
RBC	Erythrocytes (c/pl)
HB	Haemaglobin (g/dl)
HCT	Haematocrit (%)
MCV	Mean corpuscular volume (fl)
MCH	Mean corpuscular hemoglobin (pg)
MCHC	Mean corpuscular hemoglobin concentration (g/dl)
PLT	Thrombocytes (c/nl)
RDWSD	Red cell distribution width standard deviation (fl)
RDWCV	Red cell distribution width coefficient of variation (%)
PDW	Thrombocyte distribution width (fl)
MPV	Mean thrombocyte volume (fl)
NEUT_p	Neutrophils (%)
LYMPH_p	Lymphocytes (%)
MONO_p	Monocytes (%)
EOS_p	Eosinophils (%)
BASO_p	Basophils (%)
IGS_p	Immature granulocytes (%)
NRBC_p	Erythroblasts (c/100 WBC)
RETI_p	Reticulocytes (c/100 RBC)
NEUT_c	Neutrophils (c/nl)
LYMPH_c	Lymphocytes (c/nl)
MONO_c	Monocytes (c/nl)
EOS_c	Eosinophils (c/nl)
BASO_c	Basophils (c/nl)
IGS_c	Immature granulocytes (c/nl)
NRBC_c	Erythroblasts (c/nl)

ShortName	LongName
APH_WBC	Apheresate leucocytes (c/nl)
APH_RBC	Apheresate erythrocytes (c/nl)
APH_HB	Apheresate haemaglobin (g/dl)
APH_HCT	Apheresate haematocrit (%)
APH_MCV	Apheresate mean corpuscular volume (fl)
APH_MCH	Apheresate mean corpuscular hemoglobin (pg)
APH_MCHC	Apheresate mean corpuscular hemoglobin concentration (g/dl)
APH_PLTO	Apheresate thrombocytes optical (c/nl)
APH_PLTI	Apheresate thrombocytes impedance (c/nl)
APH_RETI	Apheresate reticulocytes (c/nl)
TP_AB	anti-Treponema pallidum
HBSAG	Hepatitis B Virus (HBV) surface antigen
HBC_AB	anti-Hepatitis B Virus (HBV) core antigen
HIV_AB	anti-Human Immunodeficiency Virus-1/2 (HIV) and p24-Ag
HCV_AB	anti-Hepatitis C Virus (HCV)
CMV_AB	anti-Cytomegalovirus (CMV) IgG

Supplementary Table 7

Supplementary Table 7 Demographic information for R1 training, validation and test sets.

**Characteristic**	**Overall**, N = 153[1]	**R1_train**, N = 50[1]	**R1_validation**, N = 50[1]	**R1_test**, N = 53[1]	**p-value**[2]
DATE	2023-07-18 to 2024-01-23	2023-07-18 to 2024-01-23	2023-07-18 to 2024-01-17	2023-07-18 to 2024-01-23	0.2
AGE	30 (25, 40)	29 (25, 41)	30 (26, 35)	31 (24, 43)	0.6
age_above_x					0.9
old	77 (50%)	24 (48%)	25 (50%)	28 (53%)	
young	76 (50%)	26 (52%)	25 (50%)	25 (47%)	
SEX					0.7
F	53 (35%)	19 (38%)	15 (30%)	19 (36%)	
M	100 (65%)	31 (62%)	35 (70%)	34 (64%)	
CMV_AB					0.6
negative	98 (64%)	33 (66%)	34 (68%)	31 (58%)	
positive	55 (36%)	17 (34%)	16 (32%)	22 (42%)	
device					
Navios + CytoFLEX LX	153 (100%)	50 (100%)	50 (100%)	53 (100%)	
ABO					0.3
0	64 (42%)	17 (34%)	23 (46%)	24 (45%)	
A	68 (44%)	29 (58%)	18 (36%)	21 (40%)	
AB	5 (3.3%)	1 (2.0%)	3 (6.0%)	1 (1.9%)	
B	16 (10%)	3 (6.0%)	6 (12%)	7 (13%)	
RHD					0.6
-	18 (12%)	4 (8.0%)	7 (14%)	7 (13%)	
+	135 (88%)	46 (92%)	43 (86%)	46 (87%)	
Kell					> 0.9
	145 (95%)	48 (96%)	47 (94%)	50 (94%)	
-	8 (5.2%)	2 (4.0%)	3 (6.0%)	3 (5.7%)	
IABS					
-	153 (100%)	50 (100%)	50 (100%)	53 (100%)	
HCV_PCR					
negative	153 (100%)	50 (100%)	50 (100%)	53 (100%)	
HIV_PCR					
negative	153 (100%)	50 (100%)	50 (100%)	53 (100%)	
HBV_PCR					
negative	153 (100%)	50 (100%)	50 (100%)	53 (100%)	
HEV_PCR					
negative	153 (100%)	50 (100%)	50 (100%)	53 (100%)	
WNV_PCR					
negative	153 (100%)	50 (100%)	50 (100%)	53 (100%)	
A_AB					0.2
1:1	42 (27%)	9 (18%)	14 (28%)	19 (36%)	
1:2	19 (12%)	7 (14%)	5 (10%)	7 (13%)	
1:4	5 (3.3%)	0 (0%)	3 (6.0%)	2 (3.8%)	
negative	87 (57%)	34 (68%)	28 (56%)	25 (47%)	
B_AB					0.7
1:1	55 (36%)	21 (42%)	14 (28%)	20 (38%)	
1:2	31 (20%)	9 (18%)	10 (20%)	12 (23%)	
1:4	2 (1.3%)	0 (0%)	1 (2.0%)	1 (1.9%)	
negative	65 (42%)	20 (40%)	25 (50%)	20 (38%)	
WBC	5.86 (5.04, 6.76)	5.69 (5.04, 6.80)	5.88 (5.10, 6.40)	5.92 (4.86, 6.86)	> 0.9
RBC	4.78 (4.50, 5.01)	4.80 (4.40, 5.06)	4.76 (4.52, 5.06)	4.81 (4.52, 4.98)	> 0.9
HB	14.40 (13.50, 15.00)	14.50 (13.40, 15.20)	14.20 (13.60, 14.80)	14.40 (13.50, 15.00)	0.8
HCT	41.80 (39.60, 43.50)	41.75 (38.60, 43.70)	41.85 (39.90, 43.10)	41.70 (40.10, 43.60)	> 0.9
MCV	87.4 (85.3, 89.4)	87.3 (85.3, 89.5)	87.7 (86.1, 88.9)	87.1 (85.0, 89.7)	0.8
MCH	30.20 (29.30, 31.00)	30.20 (29.50, 31.20)	30.20 (29.50, 30.80)	30.00 (28.90, 31.20)	0.4
MCHC	34.40 (33.70, 35.00)	34.55 (33.90, 35.20)	34.30 (33.60, 34.90)	34.10 (33.50, 34.90)	0.13
PLT	270 (240, 300)	271 (253, 298)	275 (227, 300)	267 (242, 309)	> 0.9
RDWSD	40.20 (38.80, 42.20)	40.05 (38.50, 42.00)	40.65 (38.80, 41.90)	39.90 (38.80, 42.40)	0.8
RDWCV	12.40 (11.90, 12.90)	12.40 (11.90, 12.70)	12.45 (12.00, 13.00)	12.40 (12.00, 12.90)	0.6
PDW	10.60 (9.60, 11.60)	10.00 (9.30, 11.20)	10.85 (10.20, 11.70)	10.70 (9.60, 11.80)	0.087
MPV	9.70 (9.00, 10.20)	9.25 (8.90, 10.10)	9.80 (9.30, 10.20)	9.70 (9.00, 10.40)	0.089
NEUT_p	58 (51, 62)	57 (52, 62)	59 (51, 62)	58 (50, 61)	0.8
LYMPH_p	30 (25, 36)	30 (25, 35)	30 (25, 36)	30 (26, 35)	> 0.9
MONO_p	9.30 (7.80, 10.80)	9.20 (7.30, 10.90)	9.25 (7.70, 10.80)	9.40 (8.00, 10.50)	> 0.9
EOS_p	2.30 (1.50, 3.60)	2.55 (1.50, 3.80)	2.20 (1.50, 3.00)	2.40 (1.30, 4.50)	0.5
BASO_p	0.50 (0.40, 0.70)	0.55 (0.40, 0.80)	0.50 (0.40, 0.60)	0.50 (0.40, 0.80)	0.5
IGS_p	0.50 (0.40, 0.60)	0.50 (0.40, 0.60)	0.50 (0.30, 0.60)	0.50 (0.40, 0.60)	0.5
NRBC_p	0.10 (0.00, 0.20)	0.10 (0.00, 0.20)	0.10 (0.00, 0.20)	0.20 (0.00, 0.20)	0.3
RETI_p	11.9 (10.1, 14.8)	11.8 (10.1, 14.6)	11.6 (9.7, 14.2)	12.7 (10.8, 15.4)	0.3
NEUT_c	3.29 (2.74, 3.93)	3.35 (2.74, 3.98)	3.36 (2.79, 3.75)	3.21 (2.58, 3.99)	> 0.9
LYMPH_c	1.71 (1.45, 2.07)	1.77 (1.44, 2.19)	1.75 (1.51, 2.06)	1.70 (1.45, 2.01)	> 0.9
MONO_c	0.54 (0.45, 0.64)	0.55 (0.45, 0.67)	0.54 (0.45, 0.61)	0.54 (0.47, 0.63)	0.9
EOS_c	0.13 (0.10, 0.21)	0.14 (0.11, 0.21)	0.12 (0.09, 0.18)	0.14 (0.08, 0.21)	0.3
BASO_c	0.030 (0.020, 0.040)	0.030 (0.020, 0.040)	0.030 (0.020, 0.040)	0.030 (0.020, 0.040)	0.5
IGS_c	0.030 (0.020, 0.040)	0.030 (0.020, 0.040)	0.030 (0.020, 0.040)	0.030 (0.020, 0.040)	0.6
NRBC_c	0.010 (0.000, 0.010)	0.010 (0.000, 0.010)	0.010 (0.000, 0.010)	0.010 (0.000, 0.010)	0.2
APH_WBC	0.010 (0.000, 0.010)	0.010 (0.000, 0.010)	0.010 (0.000, 0.010)	0.010 (0.000, 0.010)	> 0.9
APH_RBC	0.030 (0.020, 0.040)	0.020 (0.020, 0.040)	0.030 (0.020, 0.050)	0.030 (0.020, 0.040)	0.063
APH_HB	0.0000 (0.0000, 0.0000)	0.0000 (0.0000, 0.0000)	0.0000 (0.0000, 0.0000)	0.0000 (0.0000, 0.0000)	0.2
APH_HCT	0.10 (0.10, 0.20)	0.10 (0.10, 0.10)	0.10 (0.10, 0.20)	0.10 (0.10, 0.10)	0.2
APH_MCV	33 (29, 50)	40 (29, 50)	33 (33, 50)	33 (25, 40)	0.080
APH_MCH	0.0000 (0.0000, 0.0000)	0.0000 (0.0000, 0.0000)	0.0000 (0.0000, 0.0000)	0.0000 (0.0000, 0.0000)	0.2
APH_MCHC	0 (0, 0)	0 (0, 0)	0 (0, 0)	0 (0, 0)	0.3

APH_PLTO	1,183 (1,106, 1,244)	1,190 (1,097, 1,256)	1,209 (1,125, 1,243)	1,153 (1,106, 1,233)	0.4
APH_PLTI	1,000 (932, 1,069)	995 (914, 1,069)	1,023 (965, 1,089)	981 (934, 1,052)	0.14
APH_RETI					0.2
—-	151 (99%)	50 (100%)	48 (96%)	53 (100%)	
++++	2 (1.3%)	0 (0%)	2 (4.0%)	0 (0%)	
TP_AB					
negative	153 (100%)	50 (100%)	50 (100%)	53 (100%)	
HBSAG					
negative	153 (100%)	50 (100%)	50 (100%)	53 (100%)	
HBC_AB					
negative	153 (100%)	50 (100%)	50 (100%)	53 (100%)	
HIV_AB					
negative	153 (100%)	50 (100%)	50 (100%)	53 (100%)	
HCV_AB					
negative	153 (100%)	50 (100%)	50 (100%)	53 (100%)	

[1] Min to Max; Median (Q1, Q3); n (%)

[2] Kruskal-Wallis rank sum test; Pearson's Chi-squared test; NA; Fisher's exact test

Supplementary Table 8

Supplementary Table 8 Demographic information for SAN, R2 and R1 (training, validation and test). No haemogram was performed for SAN samples.

**Characteristic**	**Overall**, N = 358[1]	**R1_train**, N = 50[1]	**R1_validation**, N = 50[1]	**R1_test**, N = 53[1]	**R2_prospective**, N = 52[1]	**SAN_prospective**, N = 153[1]	**p-value**[2]
DATE	2023-07-18 to 2024-07-16	2023-07-18 to 2024-01-23	2023-07-18 to 2024-01-17	2023-07-18 to 2024-01-23	2024-05-15 to 2024-07-16	2024-04-29 to 2024-05-28	< 0.001
AGE	38 (26, 50)	29 (25, 41)	30 (26, 35)	31 (24, 43)	25 (23, 33)	48 (40, 58)	< 0.001
age_above_x							< 0.001
old	224 (63%)	24 (48%)	25 (50%)	28 (53%)	17 (33%)	130 (85%)	
young	134 (37%)	26 (52%)	25 (50%)	25 (47%)	35 (67%)	23 (15%)	
SEX							0.056
F	155 (43%)	19 (38%)	15 (30%)	19 (36%)	24 (46%)	78 (51%)	
M	203 (57%)	31 (62%)	35 (70%)	34 (64%)	28 (54%)	75 (49%)	
CMV_AB							< 0.001
negative	193 (54%)	33 (66%)	34 (68%)	31 (58%)	37 (71%)	58 (38%)	
positive	165 (46%)	17 (34%)	16 (32%)	22 (42%)	15 (29%)	95 (62%)	
device							< 0.001
DxFLEX	153 (43%)	0 (0%)	0 (0%)	0 (0%)	0 (0%)	153 (100%)	
Navios + CytoFLEX LX	205 (57%)	50 (100%)	50 (100%)	53 (100%)	52 (100%)	0 (0%)	
ABO							
	153 (43%)	0 (0%)	0 (0%)	0 (0%)	0 (0%)	153 (100%)	
0	89 (25%)	17 (34%)	23 (46%)	24 (45%)	25 (48%)	0 (0%)	
A	87 (24%)	29 (58%)	18 (36%)	21 (40%)	19 (37%)	0 (0%)	
AB	6 (1.7%)	1 (2.0%)	3 (6.0%)	1 (1.9%)	1 (1.9%)	0 (0%)	
B	23 (6.4%)	3 (6.0%)	6 (12%)	7 (13%)	7 (13%)	0 (0%)	
RHD							
	153 (43%)	0 (0%)	0 (0%)	0 (0%)	0 (0%)	153 (100%)	
-	24 (6.7%)	4 (8.0%)	7 (14%)	7 (13%)	6 (12%)	0 (0%)	
+	181 (51%)	46 (92%)	43 (86%)	46 (87%)	46 (88%)	0 (0%)	
Kell							0.003
	346 (97%)	48 (96%)	47 (94%)	50 (94%)	48 (92%)	153 (100%)	
-	12 (3.4%)	2 (4.0%)	3 (6.0%)	3 (5.7%)	4 (7.7%)	0 (0%)	
IABS							< 0.001
	153 (43%)	0 (0%)	0 (0%)	0 (0%)	0 (0%)	153 (100%)	
-	205 (57%)	50 (100%)	50 (100%)	53 (100%)	52 (100%)	0 (0%)	
HCV_PCR							< 0.001
	153 (43%)	0 (0%)	0 (0%)	0 (0%)	0 (0%)	153 (100%)	
negative	205 (57%)	50 (100%)	50 (100%)	53 (100%)	52 (100%)	0 (0%)	
HIV_PCR							< 0.001
	153 (43%)	0 (0%)	0 (0%)	0 (0%)	0 (0%)	153 (100%)	
negative	205 (57%)	50 (100%)	50 (100%)	53 (100%)	52 (100%)	0 (0%)	
HBV_PCR							< 0.001
	153 (43%)	0 (0%)	0 (0%)	0 (0%)	0 (0%)	153 (100%)	
negative	205 (57%)	50 (100%)	50 (100%)	53 (100%)	52 (100%)	0 (0%)	
HEV_PCR							< 0.001
	153 (43%)	0 (0%)	0 (0%)	0 (0%)	0 (0%)	153 (100%)	
negative	205 (57%)	50 (100%)	50 (100%)	53 (100%)	52 (100%)	0 (0%)	
WNV_PCR							< 0.001
	153 (43%)	0 (0%)	0 (0%)	0 (0%)	0 (0%)	153 (100%)	
negative	205 (57%)	50 (100%)	50 (100%)	53 (100%)	52 (100%)	0 (0%)	
A_AB							
	153 (43%)	0 (0%)	0 (0%)	0 (0%)	0 (0%)	153 (100%)	
1:1	57 (16%)	9 (18%)	14 (28%)	19 (36%)	15 (29%)	0 (0%)	
1:2	30 (8.4%)	7 (14%)	5 (10%)	7 (13%)	11 (21%)	0 (0%)	
1:4	5 (1.4%)	0 (0%)	3 (6.0%)	2 (3.8%)	0 (0%)	0 (0%)	
negative	113 (32%)	34 (68%)	28 (56%)	25 (47%)	26 (50%)	0 (0%)	
B_AB							
	153 (43%)	0 (0%)	0 (0%)	0 (0%)	0 (0%)	153 (100%)	
1:1	79 (22%)	21 (42%)	14 (28%)	20 (38%)	24 (46%)	0 (0%)	
1:2	40 (11%)	9 (18%)	10 (20%)	12 (23%)	9 (17%)	0 (0%)	
1:4	4 (1.1%)	0 (0%)	1 (2.0%)	1 (1.9%)	2 (3.8%)	0 (0%)	
negative	82 (23%)	20 (40%)	25 (50%)	20 (38%)	17 (33%)	0 (0%)	
APH_RETI							< 0.001
	153 (43%)	0 (0%)	0 (0%)	0 (0%)	0 (0%)	153 (100%)	
—-	202 (56%)	50 (100%)	48 (96%)	53 (100%)	51 (98%)	0 (0%)	
++++	3 (0.8%)	0 (0%)	2 (4.0%)	0 (0%)	1 (1.9%)	0 (0%)	
TP_AB							< 0.001
	153 (43%)	0 (0%)	0 (0%)	0 (0%)	0 (0%)	153 (100%)	
negative	205 (57%)	50 (100%)	50 (100%)	53 (100%)	52 (100%)	0 (0%)	
HBSAG							< 0.001
	153 (43%)	0 (0%)	0 (0%)	0 (0%)	0 (0%)	153 (100%)	
negative	205 (57%)	50 (100%)	50 (100%)	53 (100%)	52 (100%)	0 (0%)	
HBC_AB							< 0.001
	153 (43%)	0 (0%)	0 (0%)	0 (0%)	0 (0%)	153 (100%)	
negative	205 (57%)	50 (100%)	50 (100%)	53 (100%)	52 (100%)	0 (0%)	
HIV_AB							< 0.001
	153 (43%)	0 (0%)	0 (0%)	0 (0%)	0 (0%)	153 (100%)	
negative	205 (57%)	50 (100%)	50 (100%)	53 (100%)	52 (100%)	0 (0%)	
HCV_AB							< 0.001
	153 (43%)	0 (0%)	0 (0%)	0 (0%)	0 (0%)	153 (100%)	
negative	205 (57%)	50 (100%)	50 (100%)	53 (100%)	52 (100%)	0 (0%)	

[1]Min to Max; Median (Q1, Q3); n (%)
[2]Kruskal-Wallis rank sum test; Pearson's Chi-squared test; NA; Fisher's exact test

Supplementary Tables 9 and 10

Supplementary Table 9 Demographic information for SAN split into training, validation and test sets.

**Characteristic**	**Overall**, N = 153[1]	**SAN_train**, N = 50[1]	**SAN_validation**, N = 50[1]	**SAN_test**, N = 53[1]	**p-value**[2]
DATE	2024-04-29 to 2024-05-28	2024-04-29 to 2024-05-28	2024-04-29 to 2024-05-28	2024-04-29 to 2024-05-27	0.14
AGE	48 (40, 58)	51 (40, 59)	46 (39, 55)	48 (44, 57)	0.7
age_above_x					0.5
old	130 (85%)	43 (86%)	40 (80%)	47 (89%)	
young	23 (15%)	7 (14%)	10 (20%)	6 (11%)	
SEX					0.7
F	78 (51%)	23 (46%)	27 (54%)	28 (53%)	
M	75 (49%)	27 (54%)	23 (46%)	25 (47%)	
CMV_AB					0.4
negative	58 (38%)	23 (46%)	17 (34%)	18 (34%)	
positive	95 (62%)	27 (54%)	33 (66%)	35 (66%)	
device					
DxFLEX	153 (100%)	50 (100%)	50 (100%)	53 (100%)	

[1]Min to Max; Median (Q1, Q3); n (%)
[2]Kruskal-Wallis rank sum test; Pearson's Chi-squared test; NA

Supplementary Table 10 Demographic information for FORT and spectral (BAD) cohort cohort.

**Characteristic**	**FORT_prospective**, N = 14[1]
DATE	2025-01-14 to 2025-01-24
AGE	30 (25, 42)
age_above_x	
old	7 (50%)
young	7 (50%)
SEX	
F	4 (29%)
M	10 (71%)
CMV_AB	
negative	11 (79%)
positive	3 (21%)

[1]Min to Max; Median (Q1, Q3); n (%)

**Characteristic**	**BAD_prospective**, N = 9[1]
DATE	2025-03-18 to 2025-03-19
AGE	44 (29, 59)
age_above_x	
old	5 (56%)
young	4 (44%)
SEX	
F	4 (44%)
M	5 (56%)
CMV_AB	
negative	3 (33%)
positive	6 (67%)

[1]Min to Max; Median (Q1, Q3); n (%)

Supplementary Table 11

Please see the supplemented ModelPerformances.xlsx file for the full table. Important columns are explained here. Rows are ordered by processing, training cohort, training cytometer, applied cohort, applied cytometer and training-validation-test(-prospective) part of the data.

Column	Description
n_metacluster	Number of metaclusters used
baseline_relativized	Processing type; Baseline, Relativised or Cytonormed (=Cytonorm afterc relativisation)
train	Training cohort: R1 or SAN
applied_to	Cohort to which the model was applied
train_device	Cytometer used to generate training data
applied_device	Cytometer used to generate applied data
tvt	Train/validation/test/prospective part of the applied data
subsample_id	Subsampling 1 of subsample of cells 1 from a sample
outcome	Predicted outcome
auc	Area Under the ROC Curve (AUC)
auc_ci.low	Lower bound of the AUC 95% confidence interval
auc_ci.high	Upper bound of the AUC 95% confidence interval
threshold	Threshold used to compute binary metrics, determined on the validation split of the training cohort and device.
specificity	True Negative Rate (specificity)
sensitivity	True Positive Rate (sensitivity)
...	Further performance measures

Supplementary Table 11 Performances of all tested combinations of classifiers and test sets.

Supplementary Table 12

Supplementary Table 12 Demographic information for 95 unique patients of the ICU cohort. 5 patients were presented twice resulting into 100 total samples measured. For 2 samples no serum was conserved, therefore CMV-IgG serostatus (CMV_AB) is missing.

**Characteristic**	**N = 95**[1]
DATE	2024-10-01 to 2025-05-09
AGE	65 (57, 74)
age_above_x	
old	94 (99%)
young	1 (1.1%)
SEX	
F	36 (38%)
M	59 (62%)
CMV_AB	
	2 (2.1%)
negative	38 (40%)
positive	55 (58%)
device	
Navios	95 (100%)

[1]Min to Max; Median (Q1, Q3); n (%)
